# Supplementary material for: A new method for identifying a fault in T-connected lines based on multiscale S-transform energy entropy and an extreme learning machine
Source: PLoS One. 2019 Aug 15;14(8):e0220870. doi: 10.1371/journal.pone.0220870 (PMC6695217; doi:10.1371/journal.pone.0220870)
Supplement: S1 Table — (DOCX) [file pone.0220870.s002.docx]

**S1 Table． Simulation results of different fault type test sets.**

| **Fault branch** | | **Fault type** | | | **Fault initial angle/degree** | | **Fault distance O point / km** | | | **Transitional resistance / Ω** | | **identification result** | |
| --- | --- | --- | --- | --- | --- | --- | --- | --- | --- | --- | --- | --- | --- |
| AO | | ACG | | | 25 | | 140 | | | 200 | | AO | |
| Multiscale S-Transform Energy Entropy | | | | | | | | | | | | | |
| the traveling wave protection units | Corresponding energy entropy at each S-transformation frequency | | | | | | | | | | | | |
|  | 5/KHz | | 10/KHz | 15/KHz | | 20/KHz | | 25/KHz | 30/KHz | | 35/KHz | | 40/KHz |
| TR_1_ | 2.849659157 | | 2.651764014 | 2.466871971 | | 2.3384649 | | 2.242778026 | 2.167528693 | | 2.105706557 | | 2.052928642 |
| TR_2_ | 1.304096018 | | 1.160318917 | 1.031771072 | | 0.929551956 | | 0.843835577 | 0.769288636 | | 0.702378767 | | 0.640636447 |
| TR_3_ | 1.354217375 | | 1.214091719 | 1.10892125 | | 1.035065236 | | 0.974982648 | 0.92234996 | | 0.87489722 | | 0.831793985 |

| **Fault branch** | | **Fault type** | | | **Fault initial angle/degree** | | **Fault distance O point / km** | | | **Transitional resistance / Ω** | | **identification result** | |
| --- | --- | --- | --- | --- | --- | --- | --- | --- | --- | --- | --- | --- | --- |
| BO | | AG | | | 5 | | 110 | | | 0 | | BO | |
| Multiscale S-Transform Energy Entropy | | | | | | | | | | | | | |
| the traveling wave protection units | Corresponding energy entropy at each S-transformation frequency | | | | | | | | | | | | |
|  | 5/KHz | | 10/KHz | 15/KHz | | 20/KHz | | 25/KHz | 30/KHz | | 35/KHz | | 40/KHz |
| TR_1_ | 1.221175273 | | 1.046367688 | 0.896615742 | | 0.784716409 | | 0.697477088 | 0.626145055 | | 0.565195292 | | 0.511393751 |
| TR_2_ | 2.918832721 | | 2.739910456 | 2.571706225 | | 2.449856478 | | 2.35231503 | 2.270049131 | | 2.198642425 | | 2.135351237 |
| TR_3_ | 1.351460004 | | 1.212748256 | 1.099434333 | | 1.018110543 | | 0.953583815 | 0.899376728 | | 0.85212821 | | 0.809805684 |

| **Fault branch** | | **Fault type** | | | **Fault initial angle/degree** | | **Fault distance O point / km** | | | **Transitional resistance / Ω** | | **identification result** | |
| --- | --- | --- | --- | --- | --- | --- | --- | --- | --- | --- | --- | --- | --- |
| CO | | CG | | | 60 | | 100 | | | 300 | | CO | |
| Multiscale S-Transform Energy Entropy | | | | | | | | | | | | | |
| the traveling wave protection units | Corresponding energy entropy at each S-transformation frequency | | | | | | | | | | | | |
|  | 5/KHz | | 10/KHz | 15/KHz | | 20/KHz | | 25/KHz | 30/KHz | | 35/KHz | | 40/KHz |
| TR_1_ | 1.216996704 | | 1.031485813 | 0.880489132 | | 0.769212154 | | 0.6821365 | 0.610211805 | | 0.548266667 | | 0.4934551 |
| TR_2_ | 1.306622043 | | 1.12605943 | 0.999937324 | | 0.914975025 | | 0.84782449 | 0.78962417 | | 0.737027745 | | 0.688771889 |
| TR_3_ | 2.957944637 | | 2.817471999 | 2.65524098 | | 2.531572583 | | 2.432062473 | 2.34924041 | | 2.278211325 | | 2.215389394 |

| **Fault branch** | | **Fault type** | | | **Fault initial angle/degree** | | **Fault distance O point / km** | | | **Transitional resistance / Ω** | | **identification result** | |
| --- | --- | --- | --- | --- | --- | --- | --- | --- | --- | --- | --- | --- | --- |
| AD | | AG | | | 45 | | 400 | | | 100 | | AD | |
| Multiscale S-Transform Energy Entropy | | | | | | | | | | | | | |
| the traveling wave protection units | Corresponding energy entropy at each S-transformation frequency | | | | | | | | | | | | |
|  | 5/KHz | | 10/KHz | 15/KHz | | 20/KHz | | 25/KHz | 30/KHz | | 35/KHz | | 40/KHz |
| TR_1_ | 6.38E-05 | | 5.93E-05 | 6.34E-05 | | 7.08E-05 | | 8.03E-05 | 9.19E-05 | | 0.000105718 | | 0.000122384 |
| TR_2_ | 2.569452247 | | 2.307144505 | 2.089874581 | | 1.940642995 | | 1.824674309 | 1.727956237 | | 1.643622035 | | 1.567958964 |
| TR_3_ | 2.644677015 | | 2.442400168 | 2.248900104 | | 2.110121127 | | 2.003019075 | 1.917542544 | | 1.847579904 | | 1.789048488 |

| **Fault branch** | | **Fault type** | | | **Fault initial angle/degree** | | **Fault distance O point / km** | | | **Transitional resistance / Ω** | | **identification result** | |
| --- | --- | --- | --- | --- | --- | --- | --- | --- | --- | --- | --- | --- | --- |
| BE | | BG | | | 45 | | 250 | | | 400 | | BE | |
| Multiscale S-Transform Energy Entropy | | | | | | | | | | | | | |
| the traveling wave protection units | Corresponding energy entropy at each S-transformation frequency | | | | | | | | | | | | |
|  | 5/KHz | | 10/KHz | 15/KHz | | 20/KHz | | 25/KHz | 30/KHz | | 35/KHz | | 40/KHz |
| TR_1_ | 2.477341117 | | 2.207647314 | 1.966781403 | | 1.788275481 | | 1.649526096 | 1.536397756 | | 1.439932455 | | 1.35454492 |
| TR_2_ | 5.79E-05 | | 5.00E-05 | 5.13E-05 | | 5.58E-05 | | 6.22E-05 | 7.05E-05 | | 8.08E-05 | | 9.34E-05 |
| TR_3_ | 2.729363859 | | 2.53186493 | 2.366261133 | | 2.252945483 | | 2.164016364 | 2.089797667 | | 2.026328052 | | 1.971304179 |

| **Fault branch** | | **Fault type** | | | **Fault initial angle/degree** | | **Fault distance O point / km** | | | **Transitional resistance / Ω** | | **identification result** | |
| --- | --- | --- | --- | --- | --- | --- | --- | --- | --- | --- | --- | --- | --- |
| CF | | CG | | | 25 | | 210 | | | 200 | | CF | |
| Multiscale S-Transform Energy Entropy | | | | | | | | | | | | | |
| the traveling wave protection units | Corresponding energy entropy at each S-transformation frequency | | | | | | | | | | | | |
|  | 5/KHz | | 10/KHz | 15/KHz | | 20/KHz | | 25/KHz | 30/KHz | | 35/KHz | | 40/KHz |
| TR_1_ | 2.515435073 | | 2.278486355 | 2.051632378 | | 1.880880349 | | 1.750002625 | 1.647389034 | | 1.564983444 | | 1.497574156 |
| TR_2_ | 2.691607971 | | 2.463889935 | 2.286427526 | | 2.16760684 | | 2.073581765 | 1.992536874 | | 1.919952232 | | 1.853524158 |
| TR_3_ | 6.22E-05 | | 5.57E-05 | 5.79E-05 | | 6.29E-05 | | 6.94E-05 | 7.75E-05 | | 8.73E-05 | | 9.93E-05 |
