# Supplementary material for: A new method for identifying a fault in T-connected lines based on multiscale S-transform energy entropy and an extreme learning machine
Source: PLoS One. 2019 Aug 15;14(8):e0220870. doi: 10.1371/journal.pone.0220870 (PMC6695217; doi:10.1371/journal.pone.0220870)
Supplement: S2 Table — (DOCX) [file pone.0220870.s003.docx]

**S2 Table. Simulation results of different transitional resistance fault test sets.**

| **Fault branch** | | **Fault type** | | | **Fault initial angle/degree** | | **Fault distance O point / km** | | | **Transitional resistance / Ω** | | **identification result** | |
| --- | --- | --- | --- | --- | --- | --- | --- | --- | --- | --- | --- | --- | --- |
| AO | | AG | | | 60 | | 130 | | | 50 | | AO | |
| Multiscale S-Transform Energy Entropy | | | | | | | | | | | | | |
| the traveling wave protection units | Corresponding energy entropy at each S-transformation frequency | | | | | | | | | | | | |
|  | 5/KHz | | 10/KHz | 15/KHz | | 20/KHz | | 25/KHz | 30/KHz | | 35/KHz | | 40/KHz |
| TR_1_ | 2.837139508 | | 2.645154295 | 2.469351326 | | 2.349015805 | | 2.261067231 | 2.194026054 | | 2.140764663 | | 2.096341568 |
| TR_2_ | 1.314364036 | | 1.17482073 | 1.050510928 | | 0.955387223 | | 0.878983588 | 0.815119917 | | 0.760079788 | | 0.711676468 |
| TR_3_ | 1.359338551 | | 1.208402614 | 1.087299327 | | 0.995311692 | | 0.914543474 | 0.837992189 | | 0.76330468 | | 0.689971296 |

| **Fault branch** | | **Fault type** | | | **Fault initial angle/degree** | | **Fault distance O point / km** | | | **Transitional resistance / Ω** | | **identification result** | |
| --- | --- | --- | --- | --- | --- | --- | --- | --- | --- | --- | --- | --- | --- |
| BO | | ABG | | | 45 | | 130 | | | 0 | | BO | |
| Multiscale S-Transform Energy Entropy | | | | | | | | | | | | | |
| the traveling wave protection units | Corresponding energy entropy at each S-transformation frequency | | | | | | | | | | | | |
|  | 5/KHz | | 10/KHz | 15/KHz | | 20/KHz | | 25/KHz | 30/KHz | | 35/KHz | | 40/KHz |
| TR_1_ | 1.202392647 | | 1.025197702 | 0.879161428 | | 0.769627499 | | 0.683723859 | 0.613253464 | | 0.553491059 | | 0.502006847 |
| TR_2_ | 2.953657891 | | 2.785021608 | 2.618466761 | | 2.502211377 | | 2.413056874 | 2.340574879 | | 2.278824622 | | 2.223787049 |
| TR_3_ | 1.326167691 | | 1.174346002 | 1.051625542 | | 0.956707731 | | 0.87485778 | 0.800229461 | | 0.730491226 | | 0.664805721 |

| **Fault branch** | | **Fault type** | | | **Fault initial angle/degree** | | **Fault distance O point / km** | | | **Transitional resistance / Ω** | | **identification result** | |
| --- | --- | --- | --- | --- | --- | --- | --- | --- | --- | --- | --- | --- | --- |
| CO | | ACG | | | 25 | | 80 | | | 50 | | CO | |
| Multiscale S-Transform Energy Entropy | | | | | | | | | | | | | |
| the traveling wave protection units | Corresponding energy entropy at each S-transformation frequency | | | | | | | | | | | | |
|  | 5/KHz | | 10/KHz | 15/KHz | | 20/KHz | | 25/KHz | 30/KHz | | 35/KHz | | 40/KHz |
| TR_1_ | 1.209291334 | | 1.061361571 | 0.910649612 | | 0.793074999 | | 0.698403715 | 0.618959913 | | 0.549622205 | | 0.487338252 |
| TR_2_ | 1.295511064 | | 1.151428961 | 1.020712102 | | 0.922204312 | | 0.837785945 | 0.760345724 | | 0.686897571 | | 0.61613989 |
| TR_3_ | 2.971342945 | | 2.775781337 | 2.618973165 | | 2.510562147 | | 2.428005132 | 2.361460611 | | 2.305583467 | | 2.256765805 |

| **Fault branch** | | **Fault type** | | | **Fault initial angle/degree** | | **Fault distance O point / km** | | | **Transitional resistance / Ω** | | **identification result** | |
| --- | --- | --- | --- | --- | --- | --- | --- | --- | --- | --- | --- | --- | --- |
| AD | | ABG | | | 25 | | 430 | | | 0 | | AD | |
| Multiscale S-Transform Energy Entropy | | | | | | | | | | | | | |
| the traveling wave protection units | Corresponding energy entropy at each S-transformation frequency | | | | | | | | | | | | |
|  | 5/KHz | | 10/KHz | 15/KHz | | 20/KHz | | 25/KHz | 30/KHz | | 35/KHz | | 40/KHz |
| TR_1_ | 6.32E-05 | | 5.75E-05 | 6.14E-05 | | 6.88E-05 | | 7.85E-05 | 9.06E-05 | | 0.000105301 | | 0.000123379 |
| TR_2_ | 2.567804432 | | 2.290557723 | 2.093280685 | | 1.941380607 | | 1.822384387 | 1.721692002 | | 1.631834751 | | 1.548629746 |
| TR_3_ | 2.642226289 | | 2.427690827 | 2.257212763 | | 2.118632206 | | 2.01288621 | 1.929806335 | | 1.863375853 | | 1.809925524 |

| **Fault branch** | | **Fault type** | | | **Fault initial angle/degree** | | **Fault distance O point / km** | | | **Transitional resistance / Ω** | | **identification result** | |
| --- | --- | --- | --- | --- | --- | --- | --- | --- | --- | --- | --- | --- | --- |
| BE | | ABG | | | 60 | | 270 | | | 20 | | BE | |
| Multiscale S-Transform Energy Entropy | | | | | | | | | | | | | |
| the traveling wave protection units | Corresponding energy entropy at each S-transformation frequency | | | | | | | | | | | | |
|  | 5/KHz | | 10/KHz | 15/KHz | | 20/KHz | | 25/KHz | 30/KHz | | 35/KHz | | 40/KHz |
| TR_1_ | 2.479077749 | | 2.205946982 | 1.963014626 | | 1.78197152 | | 1.640135227 | 1.523269651 | | 1.422270497 | | 1.331352841 |
| TR_2_ | 5.97E-05 | | 5.32E-05 | 5.53E-05 | | 6.04E-05 | | 6.75E-05 | 7.65E-05 | | 8.77E-05 | | 0.00010154 |
| TR_3_ | 2.72946404 | | 2.533045023 | 2.369110934 | | 2.258115537 | | 2.172076585 | 2.101228998 | | 2.041609849 | | 1.990990155 |

| **Fault branch** | | **Fault type** | | | **Fault initial angle/degree** | | **Fault distance O point / km** | | | **Transitional resistance / Ω** | | **identification result** | |
| --- | --- | --- | --- | --- | --- | --- | --- | --- | --- | --- | --- | --- | --- |
| CF | | ABG | | | 45 | | 240 | | | 50 | | CF | |
| Multiscale S-Transform Energy Entropy | | | | | | | | | | | | | |
| the traveling wave protection units | Corresponding energy entropy at each S-transformation frequency | | | | | | | | | | | | |
|  | 5/KHz | | 10/KHz | 15/KHz | | 20/KHz | | 25/KHz | 30/KHz | | 35/KHz | | 40/KHz |
| TR_1_ | 2.516089777 | | 2.275748355 | 2.046561873 | | 1.872876161 | | 1.738022423 | 1.630107714 | | 1.540775013 | | 1.464407707 |
| TR_2_ | 2.692139683 | | 2.466046408 | 2.291059037 | | 2.175349697 | | 2.085082925 | 2.008599233 | | 1.941652888 | | 1.882308003 |
| TR_3_ | 5.96E-05 | | 5.09E-05 | 5.14E-05 | | 5.45E-05 | | 5.86E-05 | 6.33E-05 | | 6.85E-05 | | 7.41E-05 |
