# Supplementary material for: A new method for identifying a fault in T-connected lines based on multiscale S-transform energy entropy and an extreme learning machine
Source: PLoS One. 2019 Aug 15;14(8):e0220870. doi: 10.1371/journal.pone.0220870 (PMC6695217; doi:10.1371/journal.pone.0220870)
Supplement: S3 Table — (DOCX) [file pone.0220870.s004.docx]

**S3 Table. simulation results of different fault distance test sets.**

| **Fault branch** | | **Fault type** | | | **Fault initial angle/degree** | | **Fault distance O point / km** | | | **Transitional resistance / Ω** | | **identification result** | |
| --- | --- | --- | --- | --- | --- | --- | --- | --- | --- | --- | --- | --- | --- |
| AO | | ABG | | | 45 | | 270 | | | 300 | | AO | |
| Multiscale S-Transform Energy Entropy | | | | | | | | | | | | | |
| the traveling wave protection units | Corresponding energy entropy at each S-transformation frequency | | | | | | | | | | | | |
|  | 5/KHz | | 10/KHz | 15/KHz | | 20/KHz | | 25/KHz | 30/KHz | | 35/KHz | | 40/KHz |
| TR_1_ | 3.052334733 | | 2.910266111 | 2.754390996 | | 2.639120003 | | 2.543746103 | 2.460121191 | | 2.384569486 | | 2.314766938 |
| TR_2_ | 1.180510864 | | 0.996348922 | 0.84361129 | | 0.728395925 | | 0.63906237 | 0.567931496 | | 0.50970809 | | 0.461095108 |
| TR_3_ | 1.223581961 | | 1.035676433 | 0.893626724 | | 0.790418948 | | 0.708683893 | 0.640524186 | | 0.581864751 | | 0.530690743 |

| **Fault branch** | | **Fault type** | | | **Fault initial angle/degree** | | **Fault distance O point / km** | | | **Transitional resistance / Ω** | | **identification result** | |
| --- | --- | --- | --- | --- | --- | --- | --- | --- | --- | --- | --- | --- | --- |
| BO | | BCG | | | 45 | | 170 | | | 50 | | BO | |
| Multiscale S-Transform Energy Entropy | | | | | | | | | | | | | |
| the traveling wave protection units | Corresponding energy entropy at each S-transformation frequency | | | | | | | | | | | | |
|  | 5/KHz | | 10/KHz | 15/KHz | | 20/KHz | | 25/KHz | 30/KHz | | 35/KHz | | 40/KHz |
| TR_1_ | 1.451006542 | | 1.268870278 | 0.961249923 | | 0.926709583 | | 0.810059179 | 0.669042138 | | 0.6277741 | | 0.551294989 |
| TR_2_ | 2.518151894 | | 2.371563733 | 2.555997954 | | 2.376732237 | | 2.308029993 | 2.504363915 | | 2.441077131 | | 2.351884352 |
| TR_3_ | 1.597336711 | | 1.464107142 | 1.166254502 | | 1.17042989 | | 1.082886836 | 0.933419878 | | 0.900476875 | | 0.826487666 |

| **Fault branch** | | **Fault type** | | | **Fault initial angle/degree** | | **Fault distance O point / km** | | | **Transitional resistance / Ω** | | **identification result** | |
| --- | --- | --- | --- | --- | --- | --- | --- | --- | --- | --- | --- | --- | --- |
| CO | | BG | | | 5 | | 140 | | | 100 | | CO | |
| Multiscale S-Transform Energy Entropy | | | | | | | | | | | | | |
| the traveling wave protection units | Corresponding energy entropy at each S-transformation frequency | | | | | | | | | | | | |
|  | 5/KHz | | 10/KHz | 15/KHz | | 20/KHz | | 25/KHz | 30/KHz | | 35/KHz | | 40/KHz |
| TR_1_ | 1.179297723 | | 0.985809716 | 0.821838248 | | 0.696674516 | | 0.597334369 | 0.515181384 | | 0.444991431 | | 0.383886349 |
| TR_2_ | 1.264327586 | | 1.06854105 | 0.919516358 | | 0.80776825 | | 0.713525279 | 0.629126784 | | 0.551619731 | | 0.479972824 |
| TR_3_ | 3.021415105 | | 2.893812812 | 2.750983045 | | 2.647151842 | | 2.563848982 | 2.492880203 | | 2.429552896 | | 2.370690957 |

| **Fault branch** | | **Fault type** | | | **Fault initial angle/degree** | | **Fault distance O point / km** | | | **Transitional resistance / Ω** | | **identification result** | |
| --- | --- | --- | --- | --- | --- | --- | --- | --- | --- | --- | --- | --- | --- |
| AD | | BG | | | 60 | | 430 | | | 200 | | AD | |
| Multiscale S-Transform Energy Entropy | | | | | | | | | | | | | |
| the traveling wave protection units | Corresponding energy entropy at each S-transformation frequency | | | | | | | | | | | | |
|  | 5/KHz | | 10/KHz | 15/KHz | | 20/KHz | | 25/KHz | 30/KHz | | 35/KHz | | 40/KHz |
| TR_1_ | 6.17E-05 | | 5.70E-05 | 6.09E-05 | | 6.85E-05 | | 7.81E-05 | 9.01E-05 | | 0.000104713 | | 0.000122685 |
| TR_2_ | 2.576115312 | | 2.410498386 | 2.180255711 | | 2.033556555 | | 1.906485499 | 1.795605243 | | 1.695651274 | | 1.602920669 |
| TR_3_ | 2.649342345 | | 2.544689837 | 2.335834565 | | 2.20083625 | | 2.084487206 | 1.989501811 | | 1.912043408 | | 1.848752773 |

| **Fault branch** | | **Fault type** | | | **Fault initial angle/degree** | | **Fault distance O point / km** | | | **Transitional resistance / Ω** | | **identification result** | |
| --- | --- | --- | --- | --- | --- | --- | --- | --- | --- | --- | --- | --- | --- |
| BE | | CG | | | 25 | | 320 | | | 100 | | BE | |
| Multiscale S-Transform Energy Entropy | | | | | | | | | | | | | |
| the traveling wave protection units | Corresponding energy entropy at each S-transformation frequency | | | | | | | | | | | | |
|  | 5/KHz | | 10/KHz | 15/KHz | | 20/KHz | | 25/KHz | 30/KHz | | 35/KHz | | 40/KHz |
| TR_1_ | 2.44308247 | | 2.248890959 | 2.106678515 | | 1.981448713 | | 1.868538542 | 1.772948799 | | 1.692838678 | | 1.625265131 |
| TR_2_ | 6.31E-05 | | 5.46E-05 | 5.69E-05 | | 6.15E-05 | | 6.69E-05 | 7.30E-05 | | 7.98E-05 | | 8.72E-05 |
| TR_3_ | 2.675438671 | | 2.571931269 | 2.526194636 | | 2.486812445 | | 2.432368277 | 2.373707998 | | 2.315779178 | | 2.258175565 |

| **Fault branch** | | **Fault type** | | | **Fault initial angle/degree** | | **Fault distance O point / km** | | | **Transitional resistance / Ω** | | **identification result** | |
| --- | --- | --- | --- | --- | --- | --- | --- | --- | --- | --- | --- | --- | --- |
| CF | | ACG | | | 60 | | 290 | | | 50 | | CF | |
| Multiscale S-Transform Energy Entropy | | | | | | | | | | | | | |
| the traveling wave protection units | Corresponding energy entropy at each S-transformation frequency | | | | | | | | | | | | |
|  | 5/KHz | | 10/KHz | 15/KHz | | 20/KHz | | 25/KHz | 30/KHz | | 35/KHz | | 40/KHz |
| TR_1_ | 2.515698727 | | 2.281413574 | 2.044431786 | | 1.869867983 | | 1.732994147 | 1.622729103 | | 1.530815841 | | 1.451626233 |
| TR_2_ | 2.692421542 | | 2.474694494 | 2.29337699 | | 2.180043562 | | 2.091169812 | 2.016055725 | | 1.950352653 | | 1.89184929 |
| TR_3_ | 5.80E-05 | | 4.90E-05 | 5.01E-05 | | 5.45E-05 | | 6.11E-05 | 6.96E-05 | | 8.02E-05 | | 9.32E-05 |
