# Supplementary material for: A new method for identifying a fault in T-connected lines based on multiscale S-transform energy entropy and an extreme learning machine
Source: PLoS One. 2019 Aug 15;14(8):e0220870. doi: 10.1371/journal.pone.0220870 (PMC6695217; doi:10.1371/journal.pone.0220870)
Supplement: S4 Table — (DOCX) [file pone.0220870.s005.docx]

**S4 Table. simulation results of different initial angle test sets.**

| **Fault branch** | | **Fault type** | | | **Fault initial angle/degree** | | **Fault distance O point / km** | | | **Transitional resistance / Ω** | | **identification result** | |
| --- | --- | --- | --- | --- | --- | --- | --- | --- | --- | --- | --- | --- | --- |
| AO | | BG | | | 5 | | 160 | | | 350 | | AO | |
| Multiscale S-Transform Energy Entropy | | | | | | | | | | | | | |
| the traveling wave protection units | Corresponding energy entropy at each S-transformation frequency | | | | | | | | | | | | |
|  | 5/KHz | | 10/KHz | 15/KHz | | 20/KHz | | 25/KHz | 30/KHz | | 35/KHz | | 40/KHz |
| TR_1_ | 2.880794431 | | 2.691366253 | 2.51656065 | | 2.390882967 | | 2.289496788 | 2.202262187 | | 2.124678548 | | 2.05444619 |
| TR_2_ | 1.287357208 | | 1.142217277 | 1.012442995 | | 0.915231159 | | 0.841778806 | 0.785842928 | | 0.742752602 | | 0.709136952 |
| TR_3_ | 1.332716018 | | 1.181901707 | 1.062180575 | | 0.977108475 | | 0.910924444 | 0.856495513 | | 0.810021192 | | 0.769128289 |

| **Fault branch** | | **Fault type** | | | **Fault initial angle/degree** | | **Fault distance O point / km** | | | **Transitional resistance / Ω** | | **identification result** | |
| --- | --- | --- | --- | --- | --- | --- | --- | --- | --- | --- | --- | --- | --- |
| BO | | BG | | | 5 | | 100 | | | 100 | | BO | |
| Multiscale S-Transform Energy Entropy | | | | | | | | | | | | | |
| the traveling wave protection units | Corresponding energy entropy at each S-transformation frequency | | | | | | | | | | | | |
|  | 5/KHz | | 10/KHz | 15/KHz | | 20/KHz | | 25/KHz | 30/KHz | | 35/KHz | | 40/KHz |
| TR_1_ | 1.228594784 | | 1.048493617 | 0.894139065 | | 0.774966727 | | 0.678024964 | 0.595311875 | | 0.522065903 | | 0.455638403 |
| TR_2_ | 2.907189781 | | 2.737203653 | 2.575378525 | | 2.463298991 | | 2.378160732 | 2.309486351 | | 2.251528888 | | 2.200592678 |
| TR_3_ | 1.357782178 | | 1.21379738 | 1.096187157 | | 1.007673657 | | 0.932863301 | 0.865925437 | | 0.804340874 | | 0.746828584 |

| **Fault branch** | | **Fault type** | | | **Fault initial angle/degree** | | **Fault distance O point / km** | | | **Transitional resistance / Ω** | | **identification result** | |
| --- | --- | --- | --- | --- | --- | --- | --- | --- | --- | --- | --- | --- | --- |
| CO | | ABG | | | 5 | | 50 | | | 250 | | CO | |
| Multiscale S-Transform Energy Entropy | | | | | | | | | | | | | |
| the traveling wave protection units | Corresponding energy entropy at each S-transformation frequency | | | | | | | | | | | | |
|  | 5/KHz | | 10/KHz | 15/KHz | | 20/KHz | | 25/KHz | 30/KHz | | 35/KHz | | 40/KHz |
| TR_1_ | 1.169528044 | | 1.119503463 | 0.970049025 | | 0.855373807 | | 0.766738747 | 0.694604021 | | 0.633775169 | | 0.581193736 |
| TR_2_ | 1.253583921 | | 1.211120709 | 1.083663262 | | 0.989408577 | | 0.913155624 | 0.845415277 | | 0.783105518 | | 0.724951135 |
| TR_3_ | 3.02556025 | | 2.698423217 | 2.536560114 | | 2.42356044 | | 2.336124843 | 2.264545697 | | 2.203377479 | | 2.149213336 |

| **Fault branch** | | **Fault type** | | | **Fault initial angle/degree** | | **Fault distance O point / km** | | | **Transitional resistance / Ω** | | **identification result** | |
| --- | --- | --- | --- | --- | --- | --- | --- | --- | --- | --- | --- | --- | --- |
| AD | | BCG | | | 5 | | 380 | | | 0 | | AD | |
| Multiscale S-Transform Energy Entropy | | | | | | | | | | | | | |
| the traveling wave protection units | Corresponding energy entropy at each S-transformation frequency | | | | | | | | | | | | |
|  | 5/KHz | | 10/KHz | 15/KHz | | 20/KHz | | 25/KHz | 30/KHz | | 35/KHz | | 40/KHz |
| TR_1_ | 6.35E-05 | | 5.77E-05 | 6.06E-05 | | 6.63E-05 | | 7.32E-05 | 8.08E-05 | | 8.91E-05 | | 9.78E-05 |
| TR_2_ | 2.569646529 | | 2.308457592 | 2.09961877 | | 1.956615655 | | 1.848724081 | 1.762333026 | | 1.691074131 | | 1.631904084 |
| TR_3_ | 2.643663484 | | 2.434240386 | 2.239324569 | | 2.094203088 | | 1.979189958 | 1.883941399 | | 1.801871031 | | 1.728266867 |

| **Fault branch** | | **Fault type** | | | **Fault initial angle/degree** | | **Fault distance O point / km** | | | **Transitional resistance / Ω** | | **identification result** | |
| --- | --- | --- | --- | --- | --- | --- | --- | --- | --- | --- | --- | --- | --- |
| BE | | ACG | | | 5 | | 275 | | | 200 | | BE | |
| Multiscale S-Transform Energy Entropy | | | | | | | | | | | | | |
| the traveling wave protection units | Corresponding energy entropy at each S-transformation frequency | | | | | | | | | | | | |
|  | 5/KHz | | 10/KHz | 15/KHz | | 20/KHz | | 25/KHz | 30/KHz | | 35/KHz | | 40/KHz |
| TR_1_ | 2.472111648 | | 2.207294342 | 1.964757031 | | 1.784535713 | | 1.643866523 | 1.528818428 | | 1.430555566 | | 1.343559417 |
| TR_2_ | 6.14E-05 | | 5.54E-05 | 5.80E-05 | | 6.34E-05 | | 7.07E-05 | 8.00E-05 | | 9.16E-05 | | 0.000105932 |
| TR_3_ | 2.721450206 | | 2.532590633 | 2.36771775 | | 2.255870179 | | 2.168678172 | 2.096350413 | | 2.034825158 | | 1.981774199 |

| **Fault branch** | | **Fault type** | | | **Fault initial angle/degree** | | **Fault distance O point / km** | | | **Transitional resistance / Ω** | | **identification result** | |
| --- | --- | --- | --- | --- | --- | --- | --- | --- | --- | --- | --- | --- | --- |
| CF | | BCG | | | 5 | | 250 | | | 100 | | CF | |
| Multiscale S-Transform Energy Entropy | | | | | | | | | | | | | |
| the traveling wave protection units | Corresponding energy entropy at each S-transformation frequency | | | | | | | | | | | | |
|  | 5/KHz | | 10/KHz | 15/KHz | | 20/KHz | | 25/KHz | 30/KHz | | 35/KHz | | 40/KHz |
| TR_1_ | 2.511511377 | | 2.274508058 | 2.044015266 | | 1.868873936 | | 1.732381145 | 1.622697227 | | 1.531538925 | | 1.453397682 |
| TR_2_ | 2.687790072 | | 2.466890341 | 2.292820115 | | 2.178447056 | | 2.089631383 | 2.014501876 | | 1.948581771 | | 1.889646401 |
| TR_3_ | 5.86E-05 | | 4.94E-05 | 5.06E-05 | | 5.50E-05 | | 6.13E-05 | 6.94E-05 | | 7.95E-05 | | 9.18E-05 |
