# Supplementary material for: A new method for identifying a fault in T-connected lines based on multiscale S-transform energy entropy and an extreme learning machine
Source: PLoS One. 2019 Aug 15;14(8):e0220870. doi: 10.1371/journal.pone.0220870 (PMC6695217; doi:10.1371/journal.pone.0220870)
Supplement: S5 Table — (DOCX) [file pone.0220870.s006.docx]

**S5 Table. Simulation results of a test set in which the T-connection line near the O point fails.**

| **Fault branch** | | **Fault type** | | | **Fault initial angle/degree** | | **Fault distance O point / km** | | | **Transitional resistance / Ω** | | **identification result** | |
| --- | --- | --- | --- | --- | --- | --- | --- | --- | --- | --- | --- | --- | --- |
| AO | | AC | | | 60 | | 2.5 | | | 100 | | AO | |
| Multiscale S-Transform Energy Entropy | | | | | | | | | | | | | |
| the traveling wave protection units | Corresponding energy entropy at each S-transformation frequency | | | | | | | | | | | | |
|  | 5/KHz | | 10/KHz | 15/KHz | | 20/KHz | | 25/KHz | 30/KHz | | 35/KHz | | 40/KHz |
| TR_1_ | 3.019635768 | | 2.881251096 | 2.728117942 | | 2.615499935 | | 2.524662454 | 2.446727034 | | 2.376876307 | | 2.312328425 |
| TR_2_ | 1.202011278 | | 1.015139947 | 0.863364201 | | 0.750730795 | | 0.662343723 | 0.590053601 | | 0.529218146 | | 0.477001709 |
| TR_3_ | 1.244092653 | | 1.056926783 | 0.9126734 | | 0.805464884 | | 0.717449267 | 0.641641618 | | 0.574995982 | | 0.515805752 |

| **Fault branch** | | **Fault type** | | | **Fault initial angle/degree** | | **Fault distance O point / km** | | | **Transitional resistance / Ω** | | **identification result** | |
| --- | --- | --- | --- | --- | --- | --- | --- | --- | --- | --- | --- | --- | --- |
| BO | | ABG | | | 45 | | 2.5 | | | 200 | | BO | |
| Multiscale S-Transform Energy Entropy | | | | | | | | | | | | | |
| the traveling wave protection units | Corresponding energy entropy at each S-transformation frequency | | | | | | | | | | | | |
|  | 5/KHz | | 10/KHz | 15/KHz | | 20/KHz | | 25/KHz | 30/KHz | | 35/KHz | | 40/KHz |
| TR_1_ | 1.68333622 | | 1.336994209 | 1.057684854 | | 0.86768567 | | 0.743233862 | 0.672113406 | | 0.646840114 | | 0.66273332 |
| TR_2_ | 2.081713884 | | 2.257721763 | 2.362860164 | | 2.391952449 | | 2.348219319 | 2.248296909 | | 2.104775542 | | 1.92106012 |
| TR_3_ | 1.844438659 | | 1.513539766 | 1.216043129 | | 1.01687059 | | 0.911626588 | 0.884807256 | | 0.919072856 | | 1.002867115 |

| **Fault branch** | | **Fault type** | | | **Fault initial angle/degree** | | **Fault distance O point / km** | | | **Transitional resistance / Ω** | | **identification result** | |
| --- | --- | --- | --- | --- | --- | --- | --- | --- | --- | --- | --- | --- | --- |
| CO | | ACG | | | 5 | | 2.5 | | | 200 | | CO | |
| Multiscale S-Transform Energy Entropy | | | | | | | | | | | | | |
| the traveling wave protection units | Corresponding energy entropy at each S-transformation frequency | | | | | | | | | | | | |
|  | 5/KHz | | 10/KHz | 15/KHz | | 20/KHz | | 25/KHz | 30/KHz | | 35/KHz | | 40/KHz |
| TR_1_ | 1.671994193 | | 1.304109391 | 1.003712515 | | 0.805951171 | | 0.687947211 | 0.626820987 | | 0.608938865 | | 0.630924823 |
| TR_2_ | 1.782470675 | | 1.428862915 | 1.114645952 | | 0.898719064 | | 0.772871444 | 0.72224216 | | 0.729507855 | | 0.782876001 |
| TR_3_ | 2.151451978 | | 2.359293044 | 2.481533013 | | 2.514906233 | | 2.470465189 | 2.373568295 | | 2.241410922 | | 2.079460266 |
