# Supplementary material for: A new method for identifying a fault in T-connected lines based on multiscale S-transform energy entropy and an extreme learning machine
Source: PLoS One. 2019 Aug 15;14(8):e0220870. doi: 10.1371/journal.pone.0220870 (PMC6695217; doi:10.1371/journal.pone.0220870)
Supplement: S6 Table — (DOCX) [file pone.0220870.s007.docx]

**S6 Table. Simulation results of the test set under loss of AO and out-of-band branch road data in the zone.**

| **Fault branch** | **Peak data loss situation / one** | | **Fault type** | **Fault initial angle/degree** | | **Fault distance O point / km** | | | **Transitional resistance / Ω** | | **identification result** | |
| --- | --- | --- | --- | --- | --- | --- | --- | --- | --- | --- | --- | --- |
| AO | 10 | | BCG | 45 | | 150 | | | 100 | | AO | |
| Multiscale S-Transform Energy Entropy | | | | | | | | | | | | |
| the traveling wave protection units | | Corresponding energy entropy at each S-transformation frequency | | | | | | | | | | |
|  |  | 5/KHz | 10/KHz | 15/KHz | 20/KHz | | 25/KHz | 30/KHz | | 35/KHz | | 40/KHz |
| TR_1_ | | 3.033510029 | 2.827590419 | 2.522879705 | 2.437392358 | | 2.311452421 | 2.23066732 | | 2.154713672 | | 2.088475826 |
| TR_2_ | | 1.191111228 | 1.113308768 | 0.995485366 | 0.87878814 | | 0.84836227 | 0.755485258 | | 0.715438352 | | 0.682675589 |
| TR_3_ | | 1.066067536 | 1.07693553 | 1.121386466 | 0.943314918 | | 0.910939664 | 0.87562002 | | 0.818060335 | | 0.770639995 |

| **Fault branch** | **Peak data loss situation / one** | | **Fault type** | **Fault initial angle/degree** | | **Fault distance O point / km** | | | **Transitional resistance / Ω** | | **identification result** | |
| --- | --- | --- | --- | --- | --- | --- | --- | --- | --- | --- | --- | --- |
| BE | 10 | | CG | 25 | | 280 | | | 200 | | BE | |
| Multiscale S-Transform Energy Entropy | | | | | | | | | | | | |
| the traveling wave protection units | | Corresponding energy entropy at each S-transformation frequency | | | | | | | | | | |
|  |  | 5/KHz | 10/KHz | 15/KHz | 20/KHz | | 25/KHz | 30/KHz | | 35/KHz | | 40/KHz |
| TR_1_ | | 2.898944209 | 2.296834714 | 1.973246277 | 1.802440461 | | 1.670866353 | 1.56414127 | | 1.474159777 | | 1.396697116 |
| TR_2_ | | 0.000034598 | 0.000046928 | 0.000048627 | 0.000053223 | | 0.000059766 | 0.000067824 | | 0.000077394 | | 0.000088635 |
| TR_3_ | | 1.775292586 | 2.498460445 | 2.360918595 | 2.23960113 | | 2.143900178 | 2.064353409 | | 1.996144546 | | 1.935811559 |
