# Supplementary material for: A new method for identifying a fault in T-connected lines based on multiscale S-transform energy entropy and an extreme learning machine
Source: PLoS One. 2019 Aug 15;14(8):e0220870. doi: 10.1371/journal.pone.0220870 (PMC6695217; doi:10.1371/journal.pone.0220870)
Supplement: S7 Table — (DOCX) [file pone.0220870.s008.docx]

**S7 Table. Simulation results of the test set under random loss of CF data in the branch road and the outer branch road in the zone.**

| **Fault branch** | **Peak data loss situation / one** | | **Fault type** | | | **Fault initial angle/degree** | | **Fault distance O point / km** | | | **Transitional resistance / Ω** | | **identification result** | |
| --- | --- | --- | --- | --- | --- | --- | --- | --- | --- | --- | --- | --- | --- | --- |
| BO | 10 | | AG | | | 5 | | 110 | | | 100 | | BO | |
| Multiscale S-Transform Energy Entropy | | | | | | | | | | | | | | |
| the traveling wave protection units | | Corresponding energy entropy at each S-transformation frequency | | | | | | | | | | | | |
|  |  | 5/KHz | | 10/KHz | 15/KHz | | 20/KHz | | 25/KHz | 30/KHz | | 35/KHz | | 40/KHz |
| TR_1_ | | 1.096544255 | | 0.918019832 | 0.795610124 | | 0.71519346 | | 0.652660171 | 0.597976789 | | 0.547182425 | | 0.498964324 |
| TR_2_ | | 2.977868776 | | 2.796147287 | 2.6106488 | | 2.473332826 | | 2.365470049 | 2.277259011 | | 2.202872938 | | 2.138334226 |
| TR_3_ | | 1.379860325 | | 1.238934583 | 1.117207754 | | 1.028678101 | | 0.959445673 | 0.902564904 | | 0.853987794 | | 0.811110678 |

| **Fault branch** | **Peak data loss situation / one** | | **Fault type** | | | **Fault initial angle/degree** | | **Fault distance O point / km** | | | **Transitional resistance / Ω** | | **identification result** | |
| --- | --- | --- | --- | --- | --- | --- | --- | --- | --- | --- | --- | --- | --- | --- |
| CF | 10 | | ACG | | | 45 | | 230 | | | 50 | | CF | |
| Multiscale S-Transform Energy Entropy | | | | | | | | | | | | | | |
| the traveling wave protection units | | Corresponding energy entropy at each S-transformation frequency | | | | | | | | | | | | |
|  |  | 5/KHz | | 10/KHz | 15/KHz | | 20/KHz | | 25/KHz | 30/KHz | | 35/KHz | | 40/KHz |
| TR_1_ | | 2.363844331 | | 2.116198281 | 1.923839521 | | 1.795246713 | | 1.693826131 | 1.604247161 | | 1.521618146 | | 1.44395897 |
| TR_2_ | | 2.792648555 | | 2.574098983 | 2.365446533 | | 2.213330694 | | 2.099185062 | 2.011874263 | | 1.943682625 | | 1.888725662 |
| TR_3_ | | 0.000061689 | | 0.000051167 | 0.000051780 | | 0.000056016 | | 0.000062304 | 0.000070293 | | 0.000079995 | | 0.0000915409 |
