# Supplementary material for: A new method for identifying a fault in T-connected lines based on multiscale S-transform energy entropy and an extreme learning machine
Source: PLoS One. 2019 Aug 15;14(8):e0220870. doi: 10.1371/journal.pone.0220870 (PMC6695217; doi:10.1371/journal.pone.0220870)
Supplement: S8 Table — (DOCX) [file pone.0220870.s009.docx]

**S8 Table. Simulation results of the test set when the branch BO of the T-connection transmission line zone exhibits CT saturation.**

| **Fault branch** | | | **Fault type** | **Fault initial angle/degree** | | | **Fault distance O point / km** | | | **Transitional resistance / Ω** | | **identification result** | |
| --- | --- | --- | --- | --- | --- | --- | --- | --- | --- | --- | --- | --- | --- |
| AO | | | ABG | 45 | | | 160 | | | 100 | | AO | |
| Multiscale S-Transform Energy Entropy | | | | | | | | | | | | | |
| the traveling wave protection units | Corresponding energy entropy at each S-transformation frequency | | | | | | | | | | | | |
|  | 5/KHz | 10/KHz | | | 15/KHz | 20/KHz | | 25/KHz | 30/KHz | | 35/KHz | | 40/KHz |
| TR_1_ | 2.881575598 | 2.712162824 | | | 2.544180964 | 2.423927251 | | 2.331504474 | 2.257136987 | | 2.257136987 | | 2.142510729 |
| TR_2_ | 1.286458419 | 1.110904181 | | | 0.976314194 | 0.879460838 | | 0.801389954 | 0.733604006 | | 0.671932401 | | 0.614136756 |
| TR_3_ | 1.332379277 | 1.18519168 | | | 1.05945775 | 0.96428453 | | 0.886582636 | 0.819833901 | | 0.760207201 | | 0.705201808 |

| **Fault branch** | | **Fault type** | | | **Fault initial angle/degree** | | **Fault distance O point / km** | | | **Transitional resistance / Ω** | | **identification result** | |
| --- | --- | --- | --- | --- | --- | --- | --- | --- | --- | --- | --- | --- | --- |
| BO | | BC | | | 60 | | 100 | | | 100 | | BO | |
| Multiscale S-Transform Energy Entropy | | | | | | | | | | | | | |
| the traveling wave protection units | Corresponding energy entropy at each S-transformation frequency | | | | | | | | | | | | |
|  | 5/KHz | | 10/KHz | 15/KHz | | 20/KHz | | 25/KHz | 30/KHz | | 35/KHz | | 40/KHz |
| TR_1_ | 1.26465302 | | 1.088568405 | 0.939612066 | | 0.827514215 | | 0.738996696 | 0.665707671 | | 0.602525068 | | 0.546454437 |
| TR_2_ | 2.847842116 | | 2.679471328 | 2.518911335 | | 2.407022254 | | 2.321796218 | 2.253366221 | | 2.196467815 | | 2.14774688 |
| TR_3_ | 1.394871692 | | 1.24842478 | 1.128580029 | | 1.037943534 | | 0.960652269 | 0.890480405 | | 0.82448599 | | 0.760938606 |

| **Fault branch** | | **Fault type** | | | **Fault initial angle/degree** | | **Fault distance O point / km** | | | **Transitional resistance / Ω** | | **identification result** | |
| --- | --- | --- | --- | --- | --- | --- | --- | --- | --- | --- | --- | --- | --- |
| CO | | CG | | | 60 | | 100 | | | 100 | | CO | |
| Multiscale S-Transform Energy Entropy | | | | | | | | | | | | | |
| the traveling wave protection units | Corresponding energy entropy at each S-transformation frequency | | | | | | | | | | | | |
|  | 5/KHz | | 10/KHz | 15/KHz | | 20/KHz | | 25/KHz | 30/KHz | | 35/KHz | | 40/KHz |
| TR_1_ | 1.218345996 | | 1.032485327 | 0.881226718 | | 0.769733492 | | 0.68245995 | 0.610345712 | | 0.548216588 | | 0.493227167 |
| TR_2_ | 1.301362645 | | 1.121670286 | 0.996336682 | | 0.91217475 | | 0.845917795 | 0.78874511 | | 0.737339596 | | 0.690461374 |
| TR_3_ | 2.961087992 | | 2.820044808 | 2.657295275 | | 2.533125419 | | 2.433084815 | 2.349687874 | | 2.278035249 | | 2.214542531 |

| **Fault branch** | | **Fault type** | | | **Fault initial angle/degree** | | **Fault distance O point / km** | | | **Transitional resistance / Ω** | | **identification result** | |
| --- | --- | --- | --- | --- | --- | --- | --- | --- | --- | --- | --- | --- | --- |
| AD | | BCG | | | 60 | | 370 | | | 100 | | AD | |
| Multiscale S-Transform Energy Entropy | | | | | | | | | | | | | |
| the traveling wave protection units | Corresponding energy entropy at each S-transformation frequency | | | | | | | | | | | | |
|  | 5/KHz | | 10/KHz | 15/KHz | | 20/KHz | | 25/KHz | 30/KHz | | 35/KHz | | 40/KHz |
| TR_1_ | 0.000034096 | | 0.000031578 | 0.000037628 | | 0.000046045 | | 0.000056338 | 0.000068906 | | 0.000084419 | | 0.000103681 |
| TR_2_ | 3.527017842 | | 3.149447444 | 2.735501496 | | 2.447664763 | | 2.230995058 | 2.055312029 | | 1.904468477 | | 1.769538235 |
| TR_3_ | 1.568445498 | | 1.499830157 | 1.538022949 | | 1.559682824 | | 1.568901555 | 1.574255611 | | 1.579906958 | | 1.58719625 |

| **Fault branch** | | **Fault type** | | | **Fault initial angle/degree** | | **Fault distance O point / km** | | | **Transitional resistance / Ω** | | **identification result** | |
| --- | --- | --- | --- | --- | --- | --- | --- | --- | --- | --- | --- | --- | --- |
| BE | | ACG | | | 5 | | 290 | | | 100 | | BE | |
| Multiscale S-Transform Energy Entropy | | | | | | | | | | | | | |
| the traveling wave protection units | Corresponding energy entropy at each S-transformation frequency | | | | | | | | | | | | |
|  | 5/KHz | | 10/KHz | 15/KHz | | 20/KHz | | 25/KHz | 30/KHz | | 35/KHz | | 40/KHz |
| TR_1_ | 2.484541883 | | 2.210554965 | 1.961214122 | | 1.777523681 | | 1.632019653 | 1.510245015 | | 1.40291613 | | 1.304036817 |
| TR_2_ | 0.002904353 | | 0.012310778 | 0.030192722 | | 0.056864007 | | 0.093250443 | 0.140983799 | | 0.201972871 | | 0.277959858 |
| TR_3_ | 2.73279071 | | 2.535282342 | 2.36106382 | | 2.242945589 | | 2.146822174 | 2.062065433 | | 1.983761823 | | 1.908571045 |

| **Fault branch** | | **Fault type** | | | **Fault initial angle/degree** | | **Fault distance O point / km** | | | **Transitional resistance / Ω** | | **identification result** | |
| --- | --- | --- | --- | --- | --- | --- | --- | --- | --- | --- | --- | --- | --- |
| CF | | AG | | | 5 | | 250 | | | 200 | | CF | |
| Multiscale S-Transform Energy Entropy | | | | | | | | | | | | | |
| the traveling wave protection units | Corresponding energy entropy at each S-transformation frequency | | | | | | | | | | | | |
|  | 5/KHz | | 10/KHz | 15/KHz | | 20/KHz | | 25/KHz | 30/KHz | | 35/KHz | | 40/KHz |
| TR_1_ | 2.53388706 | | 2.279285293 | 2.04950155 | | 1.87519023 | | 1.739850607 | 1.631635741 | | 1.542279704 | | 1.466304336 |
| TR_2_ | 2.693029069 | | 2.462141309 | 2.287807107 | | 2.172698155 | | 2.082935956 | 2.006654032 | | 1.939374645 | | 1.87887028 |
| TR_3_ | 0.000058202 | | 0.0000495602 | 0.000050725 | | 0.000055186 | | 0.000061641 | 0.000069936 | | 0.000080246 | | 0.000092868 |
