# Supplementary material for: A new method for identifying a fault in T-connected lines based on multiscale S-transform energy entropy and an extreme learning machine
Source: PLoS One. 2019 Aug 15;14(8):e0220870. doi: 10.1371/journal.pone.0220870 (PMC6695217; doi:10.1371/journal.pone.0220870)
Supplement: S9 Table — (DOCX) [file pone.0220870.s010.docx]

**S9 Table. Simulation results of test set for branch BO and out-of-band branch AD in T-connection transmission zone under different SNR faults.**

| **Fault branch** | **SNR/(dB)** | | **Fault type** | | | **Fault initial angle/degree** | | **Fault distance O point / km** | | | **Transitional resistance / Ω** | | **identification result** | |
| --- | --- | --- | --- | --- | --- | --- | --- | --- | --- | --- | --- | --- | --- | --- |
| BO | 30 | | ABG | | | 45 | | 130 | | | 50 | | BO | |
| Multiscale S-Transform Energy Entropy | | | | | | | | | | | | | | |
| the traveling wave protection units | | Corresponding energy entropy at each S-transformation frequency | | | | | | | | | | | | |
|  |  | 5/KHz | | 10/KHz | 15/KHz | | 20/KHz | | 25/KHz | 30/KHz | | 35/KHz | | 40/KHz |
| TR_1_ | | 1.198929472 | | 1.010334094 | 0.890975752 | | 0.766397381 | | 0.679916552 | 0.640842614 | | 0.611050294 | | 0.555564777 |
| TR_2_ | | 2.948178488 | | 2.791484041 | 2.587088589 | | 2.493161885 | | 2.436921861 | 2.391637325 | | 2.26628956 | | 2.184918385 |
| TR_3_ | | 1.336402859 | | 1.182078162 | 1.085156122 | | 0.986337416 | | 0.859940655 | 0.752083287 | | 0.74210221 | | 0.727977797 |

| **Fault branch** | **SNR/(dB)** | | **Fault type** | | | **Fault initial angle/degree** | | **Fault distance O point / km** | | | **Transitional resistance / Ω** | | **identification result** | |
| --- | --- | --- | --- | --- | --- | --- | --- | --- | --- | --- | --- | --- | --- | --- |
| AD | 30 | | ABG | | | 25 | | 430 | | | 50 | | AD | |
| Multiscale S-Transform Energy Entropy | | | | | | | | | | | | | | |
| the traveling wave protection units | | Corresponding energy entropy at each S-transformation frequency | | | | | | | | | | | | |
|  |  | 5/KHz | | 10/KHz | 15/KHz | | 20/KHz | | 25/KHz | 30/KHz | | 35/KHz | | 40/KHz |
| TR_1_ | | 0.001435232 | | 0.008045053 | 0.043692673 | | 0.018029453 | | 0.03500788 | 0.076633945 | | 0.227171187 | | 0.385599983 |
| TR_2_ | | 2.563353744 | | 2.309488159 | 2.156200633 | | 1.965916940 | | 1.928664641 | 1.682559926 | | 1.819527005 | | 1.718466191 |
| TR_3_ | | 2.643214422 | | 2.402746272 | 2.170981483 | | 2.122543566 | | 1.969553966 | 2.075364286 | | 1.830304069 | | 1.827333816 |
