# Supplementary material for: A new method for identifying a fault in T-connected lines based on multiscale S-transform energy entropy and an extreme learning machine
Source: PLoS One. 2019 Aug 15;14(8):e0220870. doi: 10.1371/journal.pone.0220870 (PMC6695217; doi:10.1371/journal.pone.0220870)
Supplement: S10 Table — (DOCX) [file pone.0220870.s011.docx]

**S10 Table. The partial data obtained from Fig.4 is as follows.**

| AG phase to ground short circuit occurring on transmission line AO at a distance of 250 km from O point, fault resistance of 50 Ω (fault initial angle of 60°) | | | | |
| --- | --- | --- | --- | --- |
| N-th sampling point | Original current | original current s-transformed | Current reverse traveling wave | Current reverse traveling wave s-transformed |
| 1 | 0.579042 | 1.56E-09 | 2.986992 | 8.02E-09 |
| 2 | 0.577603 | 1.55E-09 | 2.993009 | 8.03E-09 |
| 3 | 0.576163 | 1.55E-09 | 2.999019 | 8.05E-09 |
| 4 | 0.574722 | 1.54E-09 | 3.005021 | 8.06E-09 |
| 5 | 0.573279 | 1.54E-09 | 3.011016 | 8.08E-09 |
| 6 | 0.571835 | 1.54E-09 | 3.017003 | 8.10E-09 |
| 7 | 0.570389 | 1.53E-09 | 3.022983 | 8.11E-09 |
| 8 | 0.568942 | 1.53E-09 | 3.028955 | 8.13E-09 |
| 9 | 0.567493 | 1.52E-09 | 3.03492 | 8.14E-09 |
| 10 | 0.566043 | 1.52E-09 | 3.040878 | 8.16E-09 |
| 11 | 0.564592 | 1.52E-09 | 3.046828 | 8.18E-09 |
| 12 | 0.563139 | 1.51E-09 | 3.05277 | 8.19E-09 |
| 13 | 0.561684 | 1.51E-09 | 3.058705 | 8.21E-09 |
| 14 | 0.560228 | 1.51E-09 | 3.064632 | 8.22E-09 |
| 15 | 0.558771 | 1.50E-09 | 3.070551 | 8.24E-09 |
| 16 | 0.557312 | 1.50E-09 | 3.076463 | 8.26E-09 |
| 17 | 0.555852 | 1.49E-09 | 3.082368 | 8.27E-09 |
| 18 | 0.55439 | 1.49E-09 | 3.088264 | 8.29E-09 |
| 19 | 0.552927 | 1.49E-09 | 3.094153 | 8.30E-09 |
| 20 | 0.551463 | 1.48E-09 | 3.100035 | 8.32E-09 |
| 21 | 0.549997 | 1.48E-09 | 3.105908 | 8.33E-09 |
| 22 | 0.54853 | 1.47E-09 | 3.111774 | 8.35E-09 |
| 23 | 0.547061 | 1.47E-09 | 3.117632 | 8.36E-09 |
| 24 | 0.545591 | 1.47E-09 | 3.123483 | 8.38E-09 |
| 25 | 0.54412 | 1.46E-09 | 3.129326 | 8.40E-09 |
| 26 | 0.542647 | 1.46E-09 | 3.135161 | 8.41E-09 |
| 27 | 0.541173 | 1.45E-09 | 3.140988 | 8.43E-09 |
| 28 | 0.539697 | 1.45E-09 | 3.146807 | 8.44E-09 |
| 29 | 0.53822 | 1.45E-09 | 3.152619 | 8.46E-09 |
| 30 | 0.536742 | 1.44E-09 | 3.158423 | 8.47E-09 |
| 31 | 0.535262 | 1.44E-09 | 3.164219 | 8.49E-09 |
| 32 | 0.533781 | 1.44E-09 | 3.170007 | 8.50E-09 |
| 33 | 0.532299 | 1.43E-09 | 3.175787 | 8.52E-09 |
| 34 | 0.530815 | 1.43E-09 | 3.18156 | 8.54E-09 |
| 35 | 0.52933 | 1.42E-09 | 3.187324 | 8.55E-09 |
| 36 | 0.527844 | 1.42E-09 | 3.19308 | 8.57E-09 |
| 37 | 0.526356 | 1.42E-09 | 3.198829 | 8.58E-09 |
| 38 | 0.524867 | 1.41E-09 | 3.20457 | 8.60E-09 |
| 39 | 0.523377 | 1.41E-09 | 3.210302 | 8.61E-09 |
| 40 | 0.521885 | 1.40E-09 | 3.216027 | 8.63E-09 |
| 41 | 0.520392 | 1.40E-09 | 3.221744 | 8.64E-09 |
| 42 | 0.518898 | 1.40E-09 | 3.227453 | 8.66E-09 |
| 43 | 0.517402 | 1.39E-09 | 3.233153 | 8.67E-09 |
| 44 | 0.515905 | 1.39E-09 | 3.238846 | 8.69E-09 |
| 45 | 0.514407 | 1.38E-09 | 3.24453 | 8.70E-09 |
| 46 | 0.512907 | 1.38E-09 | 3.250207 | 8.72E-09 |
| 47 | 0.511407 | 1.38E-09 | 3.255875 | 8.73E-09 |
| 48 | 0.509904 | 1.37E-09 | 3.261536 | 8.75E-09 |
| 49 | 0.508401 | 1.37E-09 | 3.267188 | 8.76E-09 |
| 50 | 0.506896 | 1.36E-09 | 3.272832 | 8.78E-09 |
| 51 | 0.505391 | 1.36E-09 | 3.278468 | 8.79E-09 |
| 52 | 0.503883 | 1.36E-09 | 3.284096 | 8.81E-09 |
| 53 | 0.502375 | 1.35E-09 | 3.289715 | 8.82E-09 |
| 54 | 0.500865 | 1.35E-09 | 3.295327 | 8.84E-09 |
| 55 | 0.499354 | 1.34E-09 | 3.30093 | 8.85E-09 |
| 56 | 0.497842 | 1.34E-09 | 3.306525 | 8.87E-09 |
| 57 | 0.496329 | 1.34E-09 | 3.312112 | 8.88E-09 |
| 58 | 0.494814 | 1.33E-09 | 3.31769 | 8.90E-09 |
| 59 | 0.493298 | 1.33E-09 | 3.323261 | 8.91E-09 |
| 60 | 0.491781 | 1.32E-09 | 3.328823 | 8.93E-09 |
| 61 | 0.490262 | 1.32E-09 | 3.334376 | 8.94E-09 |
| 62 | 0.488743 | 1.32E-09 | 3.339922 | 8.96E-09 |
| 63 | 0.487222 | 1.31E-09 | 3.345459 | 8.97E-09 |
| 64 | 0.4857 | 1.31E-09 | 3.350988 | 8.99E-09 |
| 65 | 0.484177 | 1.30E-09 | 3.356508 | 9.00E-09 |
| 66 | 0.482652 | 1.30E-09 | 3.36202 | 9.02E-09 |
| 67 | 0.481127 | 1.30E-09 | 3.367524 | 9.03E-09 |
| 68 | 0.4796 | 1.29E-09 | 3.373019 | 9.05E-09 |
| 69 | 0.478072 | 1.29E-09 | 3.378506 | 9.06E-09 |
| 70 | 0.476543 | 1.28E-09 | 3.383984 | 9.08E-09 |
| 71 | 0.475012 | 1.28E-09 | 3.389454 | 9.09E-09 |
| 72 | 0.473481 | 1.27E-09 | 3.394916 | 9.10E-09 |
| 73 | 0.471948 | 1.27E-09 | 3.400369 | 9.12E-09 |
| 74 | 0.470414 | 1.27E-09 | 3.405814 | 9.13E-09 |
| 75 | 0.468879 | 1.26E-09 | 3.41125 | 9.15E-09 |
| 76 | 0.467343 | 1.26E-09 | 3.416677 | 9.16E-09 |
| 77 | 0.465805 | 1.25E-09 | 3.422096 | 9.18E-09 |
| 78 | 0.464267 | 1.25E-09 | 3.427507 | 9.19E-09 |
| 79 | 0.462727 | 1.25E-09 | 3.432909 | 9.21E-09 |
| 80 | 0.461186 | 1.24E-09 | 3.438302 | 9.22E-09 |
| 81 | 0.459645 | 1.24E-09 | 3.443687 | 9.24E-09 |
| 82 | 0.458101 | 1.23E-09 | 3.449063 | 9.25E-09 |
| 83 | 0.456557 | 1.23E-09 | 3.454431 | 9.26E-09 |
| 84 | 0.455012 | 1.23E-09 | 3.45979 | 9.28E-09 |
| 85 | 0.453466 | 1.22E-09 | 3.46514 | 9.29E-09 |
| 86 | 0.451918 | 1.22E-09 | 3.470482 | 9.31E-09 |
| 87 | 0.450369 | 1.21E-09 | 3.475815 | 9.32E-09 |
| 88 | 0.44882 | 1.21E-09 | 3.48114 | 9.33E-09 |
| 89 | 0.447269 | 1.21E-09 | 3.486455 | 9.35E-09 |
| 90 | 0.445717 | 1.20E-09 | 3.491762 | 9.36E-09 |
| 91 | 0.444164 | 1.20E-09 | 3.497061 | 9.38E-09 |
| 92 | 0.44261 | 1.19E-09 | 3.50235 | 9.39E-09 |
| 93 | 0.441054 | 1.19E-09 | 3.507631 | 9.41E-09 |
| 94 | 0.439498 | 1.18E-09 | 3.512903 | 9.42E-09 |
| 95 | 0.437941 | 1.18E-09 | 3.518166 | 9.43E-09 |
| 96 | 0.436382 | 1.18E-09 | 3.523421 | 9.45E-09 |
| 97 | 0.434823 | 1.17E-09 | 3.528666 | 9.46E-09 |
| 98 | 0.433262 | 1.17E-09 | 3.533903 | 9.48E-09 |
| 99 | 0.431701 | 1.16E-09 | 3.539131 | 9.49E-09 |
| 100 | 0.430138 | 1.16E-09 | 3.54435 | 9.50E-09 |
| 101 | 0.428574 | 1.16E-09 | 3.54956 | 9.51E-09 |
| 102 | 0.42701 | 1.16E-09 | 3.554762 | 9.53E-09 |
| 103 | 0.425444 | 1.09E-09 | 3.559954 | 9.66E-09 |
| 104 | 0.423877 | 9.69E-10 | 3.565138 | 9.88E-09 |
| 105 | 0.422309 | 1.59E-09 | 3.570313 | 8.94E-09 |
| 106 | 0.420741 | 3.86E-09 | 3.575478 | 4.26E-09 |
| 107 | 0.419171 | 9.24E-09 | 3.580635 | 1.82E-08 |
| 108 | 0.4176 | 2.63E-08 | 3.585783 | 6.27E-08 |
| 109 | 0.416028 | 7.99E-08 | 3.590922 | 1.69E-07 |
| 110 | 0.414455 | 2.31E-07 | 3.596052 | 4.59E-07 |
| 111 | 0.412881 | 6.37E-07 | 3.601173 | 1.26E-06 |
| 112 | 0.411306 | 1.68E-06 | 3.606285 | 3.35E-06 |
| 113 | 0.40973 | 4.26E-06 | 3.611388 | 8.52E-06 |
| 114 | 0.408154 | 1.04E-05 | 3.616482 | 2.08E-05 |
| 115 | 0.406576 | 2.43E-05 | 3.621566 | 4.86E-05 |
| 116 | 0.404997 | 5.48E-05 | 3.626642 | 0.000109 |
| 117 | 0.403417 | 0.000118 | 3.631709 | 0.000237 |
| 118 | 0.401837 | 0.000246 | 3.636766 | 0.000492 |
| 119 | 0.400255 | 0.000491 | 3.641815 | 0.000981 |
| 120 | 0.398672 | 0.000941 | 3.646854 | 0.00188 |
| 121 | 0.397089 | 0.001732 | 3.651884 | 0.003461 |
| 122 | 0.395504 | 0.003062 | 3.656905 | 0.006119 |
| 123 | 0.393918 | 0.0052 | 3.661917 | 0.010389 |
| 124 | 0.392332 | 0.008477 | 3.66692 | 0.016939 |
| 125 | 0.390745 | 0.013272 | 3.671913 | 0.026519 |
| 126 | 0.389156 | 0.019949 | 3.676898 | 0.03986 |
| 127 | 0.387567 | 0.028787 | 3.681873 | 0.05752 |
| 128 | 0.385977 | 0.039879 | 3.686839 | 0.079683 |
| 129 | 0.384386 | 0.05303 | 3.691795 | 0.105958 |
| 130 | 0.382794 | 0.067685 | 3.696743 | 0.135239 |
| 131 | 0.381201 | 0.082915 | 3.701681 | 0.165668 |
| 132 | 0.379607 | 0.09748 | 3.70661 | 0.194768 |
| 133 | 0.378013 | 0.109981 | 3.711529 | 0.219744 |
| 134 | 0.376417 | 0.119076 | 3.71644 | 0.237912 |
| 135 | 0.642492 | 0.123712 | 3.18659 | 0.247174 |
| 136 | 2.320314 | 0.123333 | -0.16466 | 0.246414 |
| 137 | 2.358567 | 0.117983 | -0.24063 | 0.235722 |
| 138 | 2.36698 | 0.108301 | -0.25689 | 0.216375 |
| 139 | 2.36602 | 0.095394 | -0.25432 | 0.190586 |
| 140 | 2.361734 | 0.080631 | -0.24501 | 0.161089 |
| 141 | 2.356459 | 0.0654 | -0.23364 | 0.130659 |
| 142 | 2.351071 | 0.050907 | -0.22197 | 0.101704 |
| 143 | 2.345883 | 0.03803 | -0.21064 | 0.075975 |
| 144 | 2.340997 | 0.027266 | -0.19985 | 0.054472 |
| 145 | 2.33643 | 0.018764 | -0.18966 | 0.037485 |
| 146 | 2.332175 | 0.012394 | -0.18006 | 0.02476 |
| 147 | 2.328211 | 0.007859 | -0.171 | 0.015699 |
| 148 | 2.324518 | 0.004784 | -0.16246 | 0.009556 |
| 149 | 2.321072 | 0.002795 | -0.15439 | 0.005584 |
| 150 | 2.31785 | 0.001568 | -0.14676 | 0.003133 |
| 151 | 2.314834 | 0.000845 | -0.13951 | 0.001688 |
| 152 | 2.312001 | 0.000437 | -0.13263 | 0.000873 |
| 153 | 2.309334 | 0.000217 | -0.12606 | 0.000434 |
| 154 | 2.306815 | 0.000104 | -0.11979 | 0.000207 |
| 155 | 2.304428 | 4.74E-05 | -0.11378 | 9.47E-05 |
| 156 | 2.302159 | 2.09E-05 | -0.108 | 4.17E-05 |
| 157 | 2.299994 | 8.82E-06 | -0.10243 | 1.76E-05 |
| 158 | 2.297921 | 3.58E-06 | -0.09704 | 7.15E-06 |
| 159 | 2.295929 | 1.39E-06 | -0.09182 | 2.79E-06 |
| 160 | 2.294009 | 5.16E-07 | -0.08675 | 1.04E-06 |
| 161 | 2.292151 | 1.88E-07 | -0.0818 | 3.75E-07 |
| 162 | 2.290348 | 7.08E-08 | -0.07697 | 1.30E-07 |
| 163 | 2.288592 | 2.57E-08 | -0.07224 | 4.34E-08 |
| 164 | 2.286877 | 6.07E-09 | -0.0676 | 1.39E-08 |
| 165 | 2.285198 | 4.13E-09 | -0.06303 | 3.98E-09 |
| 166 | 2.283549 | 6.11E-09 | -0.05854 | 9.21E-10 |
| 167 | 2.281927 | 6.27E-09 | -0.0541 | 5.03E-10 |
| 168 | 2.280327 | 6.13E-09 | -0.04972 | 4.40E-10 |
| 169 | 2.278746 | 6.09E-09 | -0.04539 | 3.63E-10 |
| 170 | 2.277181 | 6.09E-09 | -0.0411 | 3.41E-10 |
| 171 | 2.275629 | 6.09E-09 | -0.03684 | 3.41E-10 |
| 172 | 2.274088 | 6.09E-09 | -0.03261 | 3.41E-10 |
| 173 | 2.272556 | 6.09E-09 | -0.02841 | 3.40E-10 |
| 174 | 2.27103 | 6.08E-09 | -0.02424 | 3.38E-10 |
| 175 | 2.26951 | 6.08E-09 | -0.02008 | 3.37E-10 |
| 176 | 2.267994 | 6.07E-09 | -0.01594 | 3.36E-10 |
| 177 | 2.266481 | 6.07E-09 | -0.01182 | 3.36E-10 |
| 178 | 2.264969 | 6.07E-09 | -0.00772 | 3.36E-10 |
| 179 | 2.263458 | 6.06E-09 | -0.00362 | 3.36E-10 |
| 180 | 2.261947 | 6.06E-09 | 0.000467 | 3.36E-10 |
| 181 | 2.260435 | 6.06E-09 | 0.004544 | 3.37E-10 |
| 182 | 2.258922 | 6.05E-09 | 0.008613 | 3.38E-10 |
| 183 | 2.257407 | 6.05E-09 | 0.012676 | 3.39E-10 |
| 184 | 2.255889 | 6.04E-09 | 0.016733 | 3.40E-10 |
| 185 | 2.254368 | 6.04E-09 | 0.020785 | 3.42E-10 |
| 186 | 2.252844 | 6.04E-09 | 0.024833 | 3.44E-10 |
| 187 | 2.251317 | 6.03E-09 | 0.028877 | 3.47E-10 |
| 188 | 2.249786 | 6.03E-09 | 0.032917 | 3.49E-10 |
| 189 | 2.248251 | 6.02E-09 | 0.036955 | 3.52E-10 |
| 190 | 2.246712 | 6.02E-09 | 0.04099 | 3.56E-10 |
| 191 | 2.245168 | 6.01E-09 | 0.045023 | 3.59E-10 |
| 192 | 2.24362 | 6.01E-09 | 0.049054 | 3.63E-10 |
| 193 | 2.242068 | 6.01E-09 | 0.053083 | 3.67E-10 |
| 194 | 2.240511 | 6.00E-09 | 0.057111 | 3.72E-10 |
| 195 | 2.238949 | 6.00E-09 | 0.061137 | 3.76E-10 |
| 196 | 2.237382 | 5.99E-09 | 0.065161 | 3.81E-10 |
| 197 | 2.235811 | 5.99E-09 | 0.069185 | 3.86E-10 |
| 198 | 2.234235 | 5.99E-09 | 0.073207 | 3.91E-10 |
| 199 | 2.232654 | 5.98E-09 | 0.077227 | 3.97E-10 |
| 200 | 2.231068 | 5.98E-09 | 0.081247 | 4.03E-10 |
