# Supplementary material for: A new method for identifying a fault in T-connected lines based on multiscale S-transform energy entropy and an extreme learning machine
Source: PLoS One. 2019 Aug 15;14(8):e0220870. doi: 10.1371/journal.pone.0220870 (PMC6695217; doi:10.1371/journal.pone.0220870)
Supplement: S11 Table — (DOCX) [file pone.0220870.s012.docx]

**S11 Table. The partial data obtained from Fig.5 is as follows.**

| AG phase to ground short circuit occurring on transmission line AO at a distance of 250 km from O point, fault resistance of 50 Ω (fault initial angle of 60°) | | | | |
| --- | --- | --- | --- | --- |
| N-th sampling point | Original current | original current s-transformed | Current reverse traveling wave | Current reverse traveling wave s-transformed |
| 301 | 0.729538 | 1.96E-09 | 2.845741 | 7.64E-09 |
| 302 | 0.728149 | 1.95E-09 | 2.851609 | 7.65E-09 |
| 303 | 0.726759 | 1.95E-09 | 2.85747 | 7.67E-09 |
| 304 | 0.725366 | 1.95E-09 | 2.863324 | 7.69E-09 |
| 305 | 0.723972 | 1.94E-09 | 2.869172 | 7.70E-09 |
| 306 | 0.722575 | 1.94E-09 | 2.875012 | 7.72E-09 |
| 307 | 0.721177 | 1.94E-09 | 2.880845 | 7.73E-09 |
| 308 | 0.719777 | 1.93E-09 | 2.88667 | 7.75E-09 |
| 309 | 0.718376 | 1.93E-09 | 2.892489 | 7.76E-09 |
| 310 | 0.716972 | 1.92E-09 | 2.898301 | 7.78E-09 |
| 311 | 0.715567 | 1.92E-09 | 2.904105 | 7.79E-09 |
| 312 | 0.714159 | 1.92E-09 | 2.909903 | 7.81E-09 |
| 313 | 0.71275 | 1.91E-09 | 2.915693 | 7.82E-09 |
| 314 | 0.711339 | 1.91E-09 | 2.921476 | 7.84E-09 |
| 315 | 0.709927 | 1.91E-09 | 2.927251 | 7.86E-09 |
| 316 | 0.708512 | 1.90E-09 | 2.93302 | 7.87E-09 |
| 317 | 0.707096 | 1.90E-09 | 2.938781 | 7.89E-09 |
| 318 | 0.705678 | 1.89E-09 | 2.944535 | 7.90E-09 |
| 319 | 0.704258 | 1.89E-09 | 2.950281 | 7.92E-09 |
| 320 | 0.702836 | 1.89E-09 | 2.956021 | 7.93E-09 |
| 321 | 0.701413 | 1.88E-09 | 2.961753 | 7.95E-09 |
| 322 | 0.699987 | 1.88E-09 | 2.967478 | 7.96E-09 |
| 323 | 0.69856 | 1.87E-09 | 2.973195 | 7.98E-09 |
| 324 | 0.697132 | 1.87E-09 | 2.978905 | 7.99E-09 |
| 325 | 0.695701 | 1.87E-09 | 2.984608 | 8.01E-09 |
| 326 | 0.694269 | 1.86E-09 | 2.990303 | 8.02E-09 |
| 327 | 0.692834 | 1.86E-09 | 2.995991 | 8.04E-09 |
| 328 | 0.691398 | 1.86E-09 | 3.001671 | 8.05E-09 |
| 329 | 0.689961 | 1.85E-09 | 3.007345 | 8.07E-09 |
| 330 | 0.688521 | 1.85E-09 | 3.01301 | 8.08E-09 |
| 331 | 0.68708 | 1.84E-09 | 3.018668 | 8.10E-09 |
| 332 | 0.685637 | 1.84E-09 | 3.024319 | 8.11E-09 |
| 333 | 0.684193 | 1.84E-09 | 3.029962 | 8.13E-09 |
| 334 | 0.682746 | 1.83E-09 | 3.035598 | 8.14E-09 |
| 335 | 0.681298 | 1.83E-09 | 3.041226 | 8.16E-09 |
| 336 | 0.679849 | 1.83E-09 | 3.046847 | 8.17E-09 |
| 337 | 0.678397 | 1.82E-09 | 3.05246 | 8.19E-09 |
| 338 | 0.676944 | 1.82E-09 | 3.058066 | 8.20E-09 |
| 339 | 0.675489 | 1.81E-09 | 3.063663 | 8.22E-09 |
| 340 | 0.674032 | 1.81E-09 | 3.069254 | 8.23E-09 |
| 341 | 0.672574 | 1.81E-09 | 3.074837 | 8.25E-09 |
| 342 | 0.671114 | 1.80E-09 | 3.080412 | 8.26E-09 |
| 343 | 0.669652 | 1.80E-09 | 3.085979 | 8.28E-09 |
| 344 | 0.668189 | 1.79E-09 | 3.091539 | 8.29E-09 |
| 345 | 0.666723 | 1.79E-09 | 3.097092 | 8.31E-09 |
| 346 | 0.665257 | 1.79E-09 | 3.102636 | 8.32E-09 |
| 347 | 0.663788 | 1.78E-09 | 3.108173 | 8.34E-09 |
| 348 | 0.662318 | 1.78E-09 | 3.113702 | 8.35E-09 |
| 349 | 0.660846 | 1.77E-09 | 3.119224 | 8.37E-09 |
| 350 | 0.659373 | 1.77E-09 | 3.124737 | 8.38E-09 |
| 351 | 0.657898 | 1.77E-09 | 3.130243 | 8.40E-09 |
| 352 | 0.656421 | 1.76E-09 | 3.135741 | 8.41E-09 |
| 353 | 0.654942 | 1.76E-09 | 3.141232 | 8.43E-09 |
| 354 | 0.653462 | 1.75E-09 | 3.146714 | 8.44E-09 |
| 355 | 0.651981 | 1.75E-09 | 3.152189 | 8.46E-09 |
| 356 | 0.650497 | 1.75E-09 | 3.157656 | 8.47E-09 |
| 357 | 0.649012 | 1.74E-09 | 3.163115 | 8.48E-09 |
| 358 | 0.647526 | 1.74E-09 | 3.168567 | 8.50E-09 |
| 359 | 0.646037 | 1.73E-09 | 3.17401 | 8.51E-09 |
| 360 | 0.644548 | 1.73E-09 | 3.179446 | 8.53E-09 |
| 361 | 0.643056 | 1.73E-09 | 3.184873 | 8.54E-09 |
| 362 | 0.641563 | 1.72E-09 | 3.190293 | 8.56E-09 |
| 363 | 0.640068 | 1.72E-09 | 3.195705 | 8.57E-09 |
| 364 | 0.638572 | 1.72E-09 | 3.201109 | 8.59E-09 |
| 365 | 0.637074 | 1.71E-09 | 3.206505 | 8.60E-09 |
| 366 | 0.635575 | 1.71E-09 | 3.211893 | 8.61E-09 |
| 367 | 0.634074 | 1.70E-09 | 3.217273 | 8.63E-09 |
| 368 | 0.632571 | 1.70E-09 | 3.222645 | 8.64E-09 |
| 369 | 0.631067 | 1.70E-09 | 3.228009 | 8.66E-09 |
| 370 | 0.629561 | 1.69E-09 | 3.233365 | 8.67E-09 |
| 371 | 0.628054 | 1.69E-09 | 3.238713 | 8.69E-09 |
| 372 | 0.626545 | 1.68E-09 | 3.244053 | 8.70E-09 |
| 373 | 0.625034 | 1.68E-09 | 3.249384 | 8.71E-09 |
| 374 | 0.623522 | 1.67E-09 | 3.254708 | 8.73E-09 |
| 375 | 0.622009 | 1.67E-09 | 3.260024 | 8.74E-09 |
| 376 | 0.620493 | 1.67E-09 | 3.265331 | 8.76E-09 |
| 377 | 0.618977 | 1.66E-09 | 3.270631 | 8.77E-09 |
| 378 | 0.617458 | 1.66E-09 | 3.275922 | 8.79E-09 |
| 379 | 0.615939 | 1.65E-09 | 3.281205 | 8.80E-09 |
| 380 | 0.614417 | 1.65E-09 | 3.28648 | 8.81E-09 |
| 381 | 0.612895 | 1.65E-09 | 3.291747 | 8.83E-09 |
| 382 | 0.61137 | 1.64E-09 | 3.297006 | 8.84E-09 |
| 383 | 0.609844 | 1.64E-09 | 3.302256 | 8.86E-09 |
| 384 | 0.608317 | 1.63E-09 | 3.307499 | 8.87E-09 |
| 385 | 0.606788 | 1.63E-09 | 3.312733 | 8.88E-09 |
| 386 | 0.605258 | 1.63E-09 | 3.317958 | 8.90E-09 |
| 387 | 0.603726 | 1.62E-09 | 3.323176 | 8.91E-09 |
| 388 | 0.602192 | 1.62E-09 | 3.328385 | 8.93E-09 |
| 389 | 0.600658 | 1.61E-09 | 3.333586 | 8.94E-09 |
| 390 | 0.599121 | 1.61E-09 | 3.338779 | 8.95E-09 |
| 391 | 0.597583 | 1.61E-09 | 3.343963 | 8.97E-09 |
| 392 | 0.596044 | 1.60E-09 | 3.349139 | 8.98E-09 |
| 393 | 0.594503 | 1.60E-09 | 3.354307 | 8.99E-09 |
| 394 | 0.592961 | 1.59E-09 | 3.359466 | 9.01E-09 |
| 395 | 0.591417 | 1.59E-09 | 3.364617 | 9.02E-09 |
| 396 | 0.589872 | 1.59E-09 | 3.36976 | 9.04E-09 |
| 397 | 0.588326 | 1.58E-09 | 3.374894 | 9.05E-09 |
| 398 | 0.586778 | 1.58E-09 | 3.38002 | 9.06E-09 |
| 399 | 0.585228 | 1.57E-09 | 3.385137 | 9.08E-09 |
| 400 | 0.583677 | 1.57E-09 | 3.390246 | 9.09E-09 |
| 401 | 0.582125 | 1.56E-09 | 3.395347 | 9.10E-09 |
| 402 | 0.580571 | 1.56E-09 | 3.400439 | 9.12E-09 |
| 403 | 0.579016 | 1.56E-09 | 3.405522 | 9.13E-09 |
| 404 | 0.577459 | 1.55E-09 | 3.410597 | 9.15E-09 |
| 405 | 0.575901 | 1.55E-09 | 3.415664 | 9.16E-09 |
| 406 | 0.574341 | 1.54E-09 | 3.420722 | 9.17E-09 |
| 407 | 0.57278 | 1.54E-09 | 3.425771 | 9.19E-09 |
| 408 | 0.571218 | 1.54E-09 | 3.430812 | 9.20E-09 |
| 409 | 0.569654 | 1.53E-09 | 3.435845 | 9.21E-09 |
| 410 | 0.568089 | 1.53E-09 | 3.440868 | 9.23E-09 |
| 411 | 0.566523 | 1.52E-09 | 3.445884 | 9.24E-09 |
| 412 | 0.564955 | 1.52E-09 | 3.45089 | 9.25E-09 |
| 413 | 0.563386 | 1.51E-09 | 3.455888 | 9.27E-09 |
| 414 | 0.561815 | 1.51E-09 | 3.460878 | 9.28E-09 |
| 415 | 0.560243 | 1.51E-09 | 3.465859 | 9.29E-09 |
| 416 | 0.55867 | 1.50E-09 | 3.470831 | 9.31E-09 |
| 417 | 0.557095 | 1.50E-09 | 3.475794 | 9.32E-09 |
| 418 | 0.555519 | 1.49E-09 | 3.480749 | 9.33E-09 |
| 419 | 0.553941 | 1.49E-09 | 3.485695 | 9.35E-09 |
| 420 | 0.552363 | 1.49E-09 | 3.490632 | 9.36E-09 |
| 421 | 0.550783 | 1.48E-09 | 3.495561 | 9.37E-09 |
| 422 | 0.549201 | 1.48E-09 | 3.500481 | 9.39E-09 |
| 423 | 0.547618 | 1.47E-09 | 3.505392 | 9.40E-09 |
| 424 | 0.546034 | 1.47E-09 | 3.510295 | 9.41E-09 |
| 425 | 0.544449 | 1.46E-09 | 3.515188 | 9.42E-09 |
| 426 | 0.542862 | 1.46E-09 | 3.520073 | 9.44E-09 |
| 427 | 0.541274 | 1.46E-09 | 3.524949 | 9.45E-09 |
| 428 | 0.539685 | 1.45E-09 | 3.529817 | 9.46E-09 |
| 429 | 0.538094 | 1.45E-09 | 3.534675 | 9.48E-09 |
| 430 | 0.536502 | 1.44E-09 | 3.539525 | 9.49E-09 |
| 431 | 0.534909 | 1.44E-09 | 3.544366 | 9.50E-09 |
| 432 | 0.533314 | 1.43E-09 | 3.549198 | 9.52E-09 |
| 433 | 0.531718 | 1.43E-09 | 3.554021 | 9.53E-09 |
| 434 | 0.530121 | 1.43E-09 | 3.558835 | 9.54E-09 |
| 435 | 0.528522 | 1.42E-09 | 3.56364 | 9.55E-09 |
| 436 | 0.526923 | 1.42E-09 | 3.568437 | 9.57E-09 |
| 437 | 0.525322 | 1.41E-09 | 3.573224 | 9.58E-09 |
| 438 | 0.52372 | 1.41E-09 | 3.578002 | 9.59E-09 |
| 439 | 0.522116 | 1.40E-09 | 3.582772 | 9.60E-09 |
| 440 | 0.520511 | 1.40E-09 | 3.587533 | 9.62E-09 |
| 441 | 0.518905 | 1.40E-09 | 3.592284 | 9.63E-09 |
| 442 | 0.517298 | 1.39E-09 | 3.597027 | 9.64E-09 |
| 443 | 0.51569 | 1.39E-09 | 3.601761 | 9.66E-09 |
| 444 | 0.51408 | 1.38E-09 | 3.606485 | 9.67E-09 |
| 445 | 0.512469 | 1.38E-09 | 3.611201 | 9.68E-09 |
| 446 | 0.510857 | 1.37E-09 | 3.615907 | 9.69E-09 |
| 447 | 0.509243 | 1.37E-09 | 3.620605 | 9.71E-09 |
| 448 | 0.507629 | 1.37E-09 | 3.625293 | 9.72E-09 |
| 449 | 0.506013 | 1.36E-09 | 3.629973 | 9.73E-09 |
| 450 | 0.504396 | 1.36E-09 | 3.634643 | 9.74E-09 |
| 451 | 0.502778 | 1.35E-09 | 3.639304 | 9.76E-09 |
| 452 | 0.501158 | 1.35E-09 | 3.643956 | 9.77E-09 |
| 453 | 0.499538 | 1.34E-09 | 3.648599 | 9.78E-09 |
| 454 | 0.497916 | 1.34E-09 | 3.653233 | 9.79E-09 |
| 455 | 0.496293 | 1.34E-09 | 3.657858 | 9.81E-09 |
| 456 | 0.494669 | 1.33E-09 | 3.662473 | 9.82E-09 |
| 457 | 0.493044 | 1.33E-09 | 3.667079 | 9.83E-09 |
| 458 | 0.491417 | 1.32E-09 | 3.671677 | 9.84E-09 |
| 459 | 0.489789 | 1.32E-09 | 3.676265 | 9.85E-09 |
| 460 | 0.488161 | 1.31E-09 | 3.680843 | 9.87E-09 |
| 461 | 0.486531 | 1.31E-09 | 3.685413 | 9.88E-09 |
| 462 | 0.484899 | 1.31E-09 | 3.689973 | 9.89E-09 |
| 463 | 0.483267 | 1.30E-09 | 3.694524 | 9.90E-09 |
| 464 | 0.481634 | 1.30E-09 | 3.699066 | 9.92E-09 |
| 465 | 0.479999 | 1.29E-09 | 3.703599 | 9.93E-09 |
| 466 | 0.478364 | 1.29E-09 | 3.708122 | 9.94E-09 |
| 467 | 0.476727 | 1.28E-09 | 3.712636 | 9.95E-09 |
| 468 | 0.475089 | 1.28E-09 | 3.71714 | 9.96E-09 |
| 469 | 0.47345 | 1.28E-09 | 3.721636 | 9.98E-09 |
| 470 | 0.47181 | 1.27E-09 | 3.726122 | 9.99E-09 |
| 471 | 0.470168 | 1.27E-09 | 3.730598 | 1.00E-08 |
| 472 | 0.468526 | 1.26E-09 | 3.735066 | 1.00E-08 |
| 473 | 0.466882 | 1.26E-09 | 3.739524 | 1.00E-08 |
| 474 | 0.465238 | 1.25E-09 | 3.743972 | 1.00E-08 |
| 475 | 0.463592 | 1.25E-09 | 3.748412 | 1.00E-08 |
| 476 | 0.461945 | 1.24E-09 | 3.752841 | 1.01E-08 |
| 477 | 0.460298 | 1.24E-09 | 3.757262 | 1.01E-08 |
| 478 | 0.458649 | 1.24E-09 | 3.761673 | 1.01E-08 |
| 479 | 0.456999 | 1.23E-09 | 3.766074 | 1.01E-08 |
| 480 | 0.455348 | 1.23E-09 | 3.770467 | 1.01E-08 |
| 481 | 0.453696 | 1.22E-09 | 3.774849 | 1.01E-08 |
| 482 | 0.452042 | 1.22E-09 | 3.779223 | 1.01E-08 |
| 483 | 0.450388 | 1.21E-09 | 3.783586 | 1.01E-08 |
| 484 | 0.448733 | 1.21E-09 | 3.787941 | 1.02E-08 |
| 485 | 0.447077 | 1.21E-09 | 3.792285 | 1.02E-08 |
| 486 | 0.445419 | 1.20E-09 | 3.796621 | 1.02E-08 |
| 487 | 0.443761 | 1.18E-09 | 3.800946 | 1.02E-08 |
| 488 | 0.442102 | 1.18E-09 | 3.805263 | 1.02E-08 |
| 489 | 0.440441 | 1.22E-09 | 3.809569 | 1.02E-08 |
| 490 | 0.43878 | 1.26E-09 | 3.813866 | 1.02E-08 |
| 491 | 0.437117 | 1.17E-09 | 3.818154 | 1.02E-08 |
| 492 | 0.435454 | 9.82E-10 | 3.822432 | 1.02E-08 |
| 493 | 0.433789 | 1.06E-09 | 3.8267 | 1.03E-08 |
| 494 | 0.432124 | 1.47E-09 | 3.830959 | 1.03E-08 |
| 495 | 0.430457 | 1.67E-09 | 3.835208 | 1.03E-08 |
| 496 | 0.42879 | 1.28E-09 | 3.839448 | 1.03E-08 |
| 497 | 0.427121 | 3.39E-10 | 3.843678 | 1.03E-08 |
| 498 | 0.425452 | 1.11E-09 | 3.847898 | 1.03E-08 |
| 499 | 0.423781 | 2.15E-09 | 3.852109 | 1.03E-08 |
| 500 | 0.42211 | 2.42E-09 | 3.85631 | 1.03E-08 |
