# Supplementary material for: A new method for identifying a fault in T-connected lines based on multiscale S-transform energy entropy and an extreme learning machine
Source: PLoS One. 2019 Aug 15;14(8):e0220870. doi: 10.1371/journal.pone.0220870 (PMC6695217; doi:10.1371/journal.pone.0220870)
Supplement: S12 Table — (DOCX) [file pone.0220870.s013.docx]

**S12 Table. The partial data obtained from Fig.6 is as follows.**

| AG phase to ground short circuit occurring on transmission line AO at a distance of 250 km from O point, fault resistance of 50 Ω (fault initial angle of 60°) | | | | |
| --- | --- | --- | --- | --- |
| N-th sampling point | Original current | original current s-transformed | Current reverse traveling wave | Current reverse traveling wave s-transformed |
| 301 | 0.815289 | 2.19E-09 | 2.80771 | 7.53E-09 |
| 302 | 0.814234 | 2.18E-09 | 2.813243 | 7.55E-09 |
| 303 | 0.813178 | 2.18E-09 | 2.818769 | 7.56E-09 |
| 304 | 0.812119 | 2.18E-09 | 2.824289 | 7.58E-09 |
| 305 | 0.811057 | 2.17E-09 | 2.829801 | 7.59E-09 |
| 306 | 0.809994 | 2.17E-09 | 2.835306 | 7.61E-09 |
| 307 | 0.808929 | 2.17E-09 | 2.840805 | 7.62E-09 |
| 308 | 0.807862 | 2.17E-09 | 2.846296 | 7.64E-09 |
| 309 | 0.806793 | 2.16E-09 | 2.85178 | 7.65E-09 |
| 310 | 0.805721 | 2.16E-09 | 2.857257 | 7.67E-09 |
| 311 | 0.804648 | 2.16E-09 | 2.862728 | 7.68E-09 |
| 312 | 0.803573 | 2.15E-09 | 2.868191 | 7.70E-09 |
| 313 | 0.802495 | 2.15E-09 | 2.873647 | 7.71E-09 |
| 314 | 0.801416 | 2.15E-09 | 2.879095 | 7.73E-09 |
| 315 | 0.800334 | 2.15E-09 | 2.884537 | 7.74E-09 |
| 316 | 0.799251 | 2.14E-09 | 2.889972 | 7.75E-09 |
| 317 | 0.798165 | 2.14E-09 | 2.895399 | 7.77E-09 |
| 318 | 0.797077 | 2.14E-09 | 2.900819 | 7.78E-09 |
| 319 | 0.795988 | 2.13E-09 | 2.906232 | 7.80E-09 |
| 320 | 0.794896 | 2.13E-09 | 2.911638 | 7.81E-09 |
| 321 | 0.793803 | 2.13E-09 | 2.917037 | 7.83E-09 |
| 322 | 0.792707 | 2.13E-09 | 2.922428 | 7.84E-09 |
| 323 | 0.791609 | 2.12E-09 | 2.927812 | 7.86E-09 |
| 324 | 0.79051 | 2.12E-09 | 2.933189 | 7.87E-09 |
| 325 | 0.789408 | 2.12E-09 | 2.938559 | 7.88E-09 |
| 326 | 0.788304 | 2.11E-09 | 2.943921 | 7.90E-09 |
| 327 | 0.787199 | 2.11E-09 | 2.949276 | 7.91E-09 |
| 328 | 0.786091 | 2.11E-09 | 2.954624 | 7.93E-09 |
| 329 | 0.784981 | 2.10E-09 | 2.959965 | 7.94E-09 |
| 330 | 0.78387 | 2.10E-09 | 2.965298 | 7.96E-09 |
| 331 | 0.782756 | 2.10E-09 | 2.970623 | 7.97E-09 |
| 332 | 0.781641 | 2.10E-09 | 2.975942 | 7.98E-09 |
| 333 | 0.780523 | 2.09E-09 | 2.981253 | 8.00E-09 |
| 334 | 0.779404 | 2.09E-09 | 2.986556 | 8.01E-09 |
| 335 | 0.778282 | 2.09E-09 | 2.991852 | 8.03E-09 |
| 336 | 0.777159 | 2.08E-09 | 2.997141 | 8.04E-09 |
| 337 | 0.776033 | 2.08E-09 | 3.002423 | 8.05E-09 |
| 338 | 0.774906 | 2.08E-09 | 3.007696 | 8.07E-09 |
| 339 | 0.773777 | 2.07E-09 | 3.012963 | 8.08E-09 |
| 340 | 0.772645 | 2.07E-09 | 3.018222 | 8.10E-09 |
| 341 | 0.771512 | 2.07E-09 | 3.023473 | 8.11E-09 |
| 342 | 0.770377 | 2.07E-09 | 3.028717 | 8.12E-09 |
| 343 | 0.76924 | 2.06E-09 | 3.033953 | 8.14E-09 |
| 344 | 0.768101 | 2.06E-09 | 3.039182 | 8.15E-09 |
| 345 | 0.76696 | 2.06E-09 | 3.044404 | 8.17E-09 |
| 346 | 0.765817 | 2.05E-09 | 3.049617 | 8.18E-09 |
| 347 | 0.764672 | 2.05E-09 | 3.054824 | 8.19E-09 |
| 348 | 0.763525 | 2.05E-09 | 3.060022 | 8.21E-09 |
| 349 | 0.762377 | 2.04E-09 | 3.065213 | 8.22E-09 |
| 350 | 0.761226 | 2.04E-09 | 3.070396 | 8.24E-09 |
| 351 | 0.760074 | 2.04E-09 | 3.075572 | 8.25E-09 |
| 352 | 0.758919 | 2.04E-09 | 3.08074 | 8.26E-09 |
| 353 | 0.757763 | 2.03E-09 | 3.085901 | 8.28E-09 |
| 354 | 0.756605 | 2.03E-09 | 3.091054 | 8.29E-09 |
| 355 | 0.755445 | 2.03E-09 | 3.096199 | 8.30E-09 |
| 356 | 0.754283 | 2.02E-09 | 3.101336 | 8.32E-09 |
| 357 | 0.753119 | 2.02E-09 | 3.106466 | 8.33E-09 |
| 358 | 0.751953 | 2.02E-09 | 3.111588 | 8.35E-09 |
| 359 | 0.750785 | 2.01E-09 | 3.116702 | 8.36E-09 |
| 360 | 0.749616 | 2.01E-09 | 3.121808 | 8.37E-09 |
| 361 | 0.748444 | 2.01E-09 | 3.126907 | 8.39E-09 |
| 362 | 0.747271 | 2.00E-09 | 3.131998 | 8.40E-09 |
| 363 | 0.746096 | 2.00E-09 | 3.137081 | 8.41E-09 |
| 364 | 0.744919 | 2.00E-09 | 3.142157 | 8.43E-09 |
| 365 | 0.74374 | 1.99E-09 | 3.147224 | 8.44E-09 |
| 366 | 0.742559 | 1.99E-09 | 3.152284 | 8.45E-09 |
| 367 | 0.741376 | 1.99E-09 | 3.157336 | 8.47E-09 |
| 368 | 0.740192 | 1.99E-09 | 3.16238 | 8.48E-09 |
| 369 | 0.739006 | 1.98E-09 | 3.167416 | 8.49E-09 |
| 370 | 0.737818 | 1.98E-09 | 3.172444 | 8.51E-09 |
| 371 | 0.736628 | 1.98E-09 | 3.177464 | 8.52E-09 |
| 372 | 0.735436 | 1.97E-09 | 3.182477 | 8.53E-09 |
| 373 | 0.734242 | 1.97E-09 | 3.187481 | 8.55E-09 |
| 374 | 0.733046 | 1.97E-09 | 3.192478 | 8.56E-09 |
| 375 | 0.731849 | 1.96E-09 | 3.197467 | 8.57E-09 |
| 376 | 0.73065 | 1.96E-09 | 3.202447 | 8.59E-09 |
| 377 | 0.729449 | 1.96E-09 | 3.20742 | 8.60E-09 |
| 378 | 0.728246 | 1.95E-09 | 3.212385 | 8.61E-09 |
| 379 | 0.727041 | 1.95E-09 | 3.217341 | 8.63E-09 |
| 380 | 0.725835 | 1.95E-09 | 3.22229 | 8.64E-09 |
| 381 | 0.724627 | 1.94E-09 | 3.227231 | 8.65E-09 |
| 382 | 0.723417 | 1.94E-09 | 3.232163 | 8.67E-09 |
| 383 | 0.722205 | 1.94E-09 | 3.237088 | 8.68E-09 |
| 384 | 0.720991 | 1.93E-09 | 3.242004 | 8.69E-09 |
| 385 | 0.719776 | 1.93E-09 | 3.246913 | 8.71E-09 |
| 386 | 0.718559 | 1.93E-09 | 3.251813 | 8.72E-09 |
| 387 | 0.71734 | 1.92E-09 | 3.256705 | 8.73E-09 |
| 388 | 0.716119 | 1.92E-09 | 3.261589 | 8.75E-09 |
| 389 | 0.714896 | 1.92E-09 | 3.266465 | 8.76E-09 |
| 390 | 0.713672 | 1.91E-09 | 3.271333 | 8.77E-09 |
| 391 | 0.712446 | 1.91E-09 | 3.276193 | 8.78E-09 |
| 392 | 0.711218 | 1.91E-09 | 3.281044 | 8.80E-09 |
| 393 | 0.709988 | 1.90E-09 | 3.285887 | 8.81E-09 |
| 394 | 0.708757 | 1.90E-09 | 3.290722 | 8.82E-09 |
| 395 | 0.707524 | 1.90E-09 | 3.295549 | 8.84E-09 |
| 396 | 0.706289 | 1.89E-09 | 3.300368 | 8.85E-09 |
| 397 | 0.705052 | 1.89E-09 | 3.305178 | 8.86E-09 |
| 398 | 0.703814 | 1.89E-09 | 3.309981 | 8.87E-09 |
| 399 | 0.702574 | 1.88E-09 | 3.314774 | 8.89E-09 |
| 400 | 0.701332 | 1.88E-09 | 3.31956 | 8.90E-09 |
| 401 | 0.700089 | 1.88E-09 | 3.324337 | 8.91E-09 |
| 402 | 0.698843 | 1.87E-09 | 3.329106 | 8.93E-09 |
| 403 | 0.697596 | 1.87E-09 | 3.333867 | 8.94E-09 |
| 404 | 0.696347 | 1.87E-09 | 3.33862 | 8.95E-09 |
| 405 | 0.695097 | 1.86E-09 | 3.343364 | 8.96E-09 |
| 406 | 0.693845 | 1.86E-09 | 3.348099 | 8.98E-09 |
| 407 | 0.692591 | 1.86E-09 | 3.352827 | 8.99E-09 |
| 408 | 0.691335 | 1.85E-09 | 3.357546 | 9.00E-09 |
| 409 | 0.690078 | 1.85E-09 | 3.362256 | 9.01E-09 |
| 410 | 0.688819 | 1.85E-09 | 3.366958 | 9.03E-09 |
| 411 | 0.687558 | 1.84E-09 | 3.371652 | 9.04E-09 |
| 412 | 0.686296 | 1.84E-09 | 3.376337 | 9.05E-09 |
| 413 | 0.685032 | 1.84E-09 | 3.381014 | 9.06E-09 |
| 414 | 0.683766 | 1.83E-09 | 3.385683 | 9.08E-09 |
| 415 | 0.682499 | 1.83E-09 | 3.390343 | 9.09E-09 |
| 416 | 0.68123 | 1.83E-09 | 3.394994 | 9.10E-09 |
| 417 | 0.679959 | 1.82E-09 | 3.399637 | 9.11E-09 |
| 418 | 0.678687 | 1.82E-09 | 3.404272 | 9.13E-09 |
| 419 | 0.677413 | 1.82E-09 | 3.408898 | 9.14E-09 |
| 420 | 0.676137 | 1.81E-09 | 3.413515 | 9.15E-09 |
| 421 | 0.67486 | 1.81E-09 | 3.418124 | 9.16E-09 |
| 422 | 0.673581 | 1.81E-09 | 3.422724 | 9.18E-09 |
| 423 | 0.6723 | 1.80E-09 | 3.427316 | 9.19E-09 |
| 424 | 0.671018 | 1.80E-09 | 3.431899 | 9.20E-09 |
| 425 | 0.669734 | 1.80E-09 | 3.436474 | 9.21E-09 |
| 426 | 0.668448 | 1.79E-09 | 3.44104 | 9.22E-09 |
| 427 | 0.667161 | 1.79E-09 | 3.445598 | 9.24E-09 |
| 428 | 0.665872 | 1.79E-09 | 3.450146 | 9.25E-09 |
| 429 | 0.664582 | 1.78E-09 | 3.454687 | 9.26E-09 |
| 430 | 0.663289 | 1.78E-09 | 3.459218 | 9.27E-09 |
| 431 | 0.661996 | 1.78E-09 | 3.463741 | 9.29E-09 |
| 432 | 0.6607 | 1.77E-09 | 3.468255 | 9.30E-09 |
| 433 | 0.659404 | 1.77E-09 | 3.472761 | 9.31E-09 |
| 434 | 0.658105 | 1.77E-09 | 3.477257 | 9.32E-09 |
| 435 | 0.656805 | 1.76E-09 | 3.481745 | 9.33E-09 |
| 436 | 0.655503 | 1.76E-09 | 3.486225 | 9.35E-09 |
| 437 | 0.6542 | 1.76E-09 | 3.490695 | 9.36E-09 |
| 438 | 0.652895 | 1.75E-09 | 3.495157 | 9.37E-09 |
| 439 | 0.651589 | 1.75E-09 | 3.499611 | 9.38E-09 |
| 440 | 0.650281 | 1.75E-09 | 3.504055 | 9.39E-09 |
| 441 | 0.648971 | 1.74E-09 | 3.508491 | 9.40E-09 |
| 442 | 0.64766 | 1.74E-09 | 3.512917 | 9.42E-09 |
| 443 | 0.646347 | 1.73E-09 | 3.517335 | 9.43E-09 |
| 444 | 0.645033 | 1.73E-09 | 3.521745 | 9.44E-09 |
| 445 | 0.643717 | 1.73E-09 | 3.526145 | 9.45E-09 |
| 446 | 0.642399 | 1.72E-09 | 3.530536 | 9.46E-09 |
| 447 | 0.64108 | 1.72E-09 | 3.534919 | 9.48E-09 |
| 448 | 0.63976 | 1.72E-09 | 3.539293 | 9.49E-09 |
| 449 | 0.638438 | 1.71E-09 | 3.543658 | 9.50E-09 |
| 450 | 0.637114 | 1.71E-09 | 3.548014 | 9.51E-09 |
| 451 | 0.635789 | 1.71E-09 | 3.552361 | 9.52E-09 |
| 452 | 0.634462 | 1.70E-09 | 3.5567 | 9.53E-09 |
| 453 | 0.633134 | 1.70E-09 | 3.561029 | 9.55E-09 |
| 454 | 0.631805 | 1.70E-09 | 3.565349 | 9.56E-09 |
| 455 | 0.630473 | 1.69E-09 | 3.569661 | 9.57E-09 |
| 456 | 0.629141 | 1.69E-09 | 3.573963 | 9.58E-09 |
| 457 | 0.627806 | 1.69E-09 | 3.578257 | 9.59E-09 |
| 458 | 0.626471 | 1.68E-09 | 3.582542 | 9.60E-09 |
| 459 | 0.625133 | 1.68E-09 | 3.586817 | 9.61E-09 |
| 460 | 0.623794 | 1.67E-09 | 3.591084 | 9.63E-09 |
| 461 | 0.622454 | 1.67E-09 | 3.595342 | 9.64E-09 |
| 462 | 0.621112 | 1.67E-09 | 3.59959 | 9.65E-09 |
| 463 | 0.619769 | 1.66E-09 | 3.60383 | 9.66E-09 |
| 464 | 0.618424 | 1.66E-09 | 3.60806 | 9.67E-09 |
| 465 | 0.617078 | 1.66E-09 | 3.612282 | 9.68E-09 |
| 466 | 0.615731 | 1.65E-09 | 3.616494 | 9.69E-09 |
| 467 | 0.614381 | 1.65E-09 | 3.620698 | 9.70E-09 |
| 468 | 0.613031 | 1.65E-09 | 3.624892 | 9.72E-09 |
| 469 | 0.611679 | 1.64E-09 | 3.629077 | 9.73E-09 |
| 470 | 0.610325 | 1.64E-09 | 3.633253 | 9.74E-09 |
| 471 | 0.60897 | 1.64E-09 | 3.63742 | 9.75E-09 |
| 472 | 0.607614 | 1.63E-09 | 3.641578 | 9.76E-09 |
| 473 | 0.606256 | 1.63E-09 | 3.645727 | 9.77E-09 |
| 474 | 0.604896 | 1.62E-09 | 3.649866 | 9.78E-09 |
| 475 | 0.603536 | 1.62E-09 | 3.653997 | 9.79E-09 |
| 476 | 0.602173 | 1.62E-09 | 3.658118 | 9.80E-09 |
| 477 | 0.60081 | 1.61E-09 | 3.66223 | 9.82E-09 |
| 478 | 0.599445 | 1.61E-09 | 3.666333 | 9.83E-09 |
| 479 | 0.598078 | 1.61E-09 | 3.670427 | 9.84E-09 |
| 480 | 0.59671 | 1.60E-09 | 3.674511 | 9.85E-09 |
| 481 | 0.595341 | 1.60E-09 | 3.678586 | 9.86E-09 |
| 482 | 0.59397 | 1.60E-09 | 3.682652 | 9.87E-09 |
| 483 | 0.592598 | 1.59E-09 | 3.686709 | 9.88E-09 |
| 484 | 0.591225 | 1.59E-09 | 3.690757 | 9.89E-09 |
| 485 | 0.58985 | 1.58E-09 | 3.694795 | 9.90E-09 |
| 486 | 0.588473 | 1.58E-09 | 3.698824 | 9.91E-09 |
| 487 | 0.587096 | 1.58E-09 | 3.702844 | 9.92E-09 |
| 488 | 0.585716 | 1.57E-09 | 3.706854 | 9.93E-09 |
| 489 | 0.584336 | 1.57E-09 | 3.710855 | 9.95E-09 |
| 490 | 0.582954 | 1.57E-09 | 3.714847 | 9.96E-09 |
| 491 | 0.581571 | 1.56E-09 | 3.718829 | 9.97E-09 |
| 492 | 0.580186 | 1.56E-09 | 3.722802 | 9.98E-09 |
| 493 | 0.5788 | 1.55E-09 | 3.726766 | 9.99E-09 |
| 494 | 0.577413 | 1.55E-09 | 3.73072 | 1.00E-08 |
| 495 | 0.576024 | 1.55E-09 | 3.734665 | 1.00E-08 |
| 496 | 0.574634 | 1.54E-09 | 3.738601 | 1.00E-08 |
| 497 | 0.573243 | 1.54E-09 | 3.742527 | 1.00E-08 |
| 498 | 0.57185 | 1.54E-09 | 3.746444 | 1.00E-08 |
| 499 | 0.570456 | 1.53E-09 | 3.750352 | 1.01E-08 |
| 500 | 0.56906 | 1.53E-09 | 3.75425 | 1.01E-08 |
