# Supplementary material for: A new method for identifying a fault in T-connected lines based on multiscale S-transform energy entropy and an extreme learning machine
Source: PLoS One. 2019 Aug 15;14(8):e0220870. doi: 10.1371/journal.pone.0220870 (PMC6695217; doi:10.1371/journal.pone.0220870)
Supplement: S13 Table — (DOCX) [file pone.0220870.s014.docx]

**S13 Table. The partial data obtained from Fig.7 is as follows.**

| AG phase to ground short circuit occurring on transmission line BE at a distance of 250 km from O point, fault resistance of 100 Ω (fault initial angle of 60°) | | | | |
| --- | --- | --- | --- | --- |
| N-th sampling point | Original current | original current s-transformed | Current reverse traveling wave | Current reverse traveling wave s-transformed |
| 350 | 0.014256 | 1.47E-10 | 4.544697 | 1.22E-08 |
| 351 | 0.012572 | 1.45E-10 | 4.547341 | 1.22E-08 |
| 352 | 0.010889 | 1.45E-10 | 4.549974 | 1.22E-08 |
| 353 | 0.009205 | 1.44E-10 | 4.552596 | 1.22E-08 |
| 354 | 0.007522 | 1.43E-10 | 4.555206 | 1.22E-08 |
| 355 | 0.005838 | 1.42E-10 | 4.557805 | 1.22E-08 |
| 356 | 0.004155 | 1.42E-10 | 4.560393 | 1.22E-08 |
| 357 | 0.002472 | 1.42E-10 | 4.562969 | 1.22E-08 |
| 358 | 0.000789 | 1.42E-10 | 4.565534 | 1.22E-08 |
| 359 | -0.00089 | 1.42E-10 | 4.568088 | 1.22E-08 |
| 360 | -0.00258 | 1.42E-10 | 4.57063 | 1.22E-08 |
| 361 | -0.00426 | 1.42E-10 | 4.573161 | 1.23E-08 |
| 362 | -0.00594 | 1.42E-10 | 4.57568 | 1.23E-08 |
| 363 | -0.00762 | 1.43E-10 | 4.578188 | 1.23E-08 |
| 364 | -0.00931 | 1.44E-10 | 4.580685 | 1.23E-08 |
| 365 | -0.01099 | 1.44E-10 | 4.58317 | 1.23E-08 |
| 366 | -0.01267 | 1.45E-10 | 4.585644 | 1.23E-08 |
| 367 | -0.01435 | 1.47E-10 | 4.588107 | 1.23E-08 |
| 368 | -0.01603 | 1.48E-10 | 4.590558 | 1.23E-08 |
| 369 | -0.01772 | 1.49E-10 | 4.592997 | 1.23E-08 |
| 370 | -0.0194 | 1.51E-10 | 4.595425 | 1.23E-08 |
| 371 | -0.02108 | 1.52E-10 | 4.597842 | 1.23E-08 |
| 372 | -0.02276 | 1.54E-10 | 4.600247 | 1.23E-08 |
| 373 | -0.02444 | 1.56E-10 | 4.602641 | 1.23E-08 |
| 374 | -0.02612 | 1.58E-10 | 4.605023 | 1.23E-08 |
| 375 | -0.0278 | 1.60E-10 | 4.607394 | 1.23E-08 |
| 376 | -0.02948 | 1.62E-10 | 4.609754 | 1.23E-08 |
| 377 | -0.03116 | 1.64E-10 | 4.612102 | 1.24E-08 |
| 378 | -0.03284 | 1.66E-10 | 4.614438 | 1.24E-08 |
| 379 | -0.03452 | 1.69E-10 | 4.616763 | 1.24E-08 |
| 380 | -0.0362 | 1.71E-10 | 4.619077 | 1.24E-08 |
| 381 | -0.03788 | 1.74E-10 | 4.621379 | 1.24E-08 |
| 382 | -0.03956 | 1.77E-10 | 4.623669 | 1.24E-08 |
| 383 | -0.04124 | 1.79E-10 | 4.625948 | 1.24E-08 |
| 384 | -0.04292 | 1.82E-10 | 4.628216 | 1.24E-08 |
| 385 | -0.04459 | 1.85E-10 | 4.630472 | 1.24E-08 |
| 386 | -0.04627 | 1.88E-10 | 4.632716 | 1.24E-08 |
| 387 | -0.04795 | 1.91E-10 | 4.634949 | 1.24E-08 |
| 388 | -0.04963 | 1.94E-10 | 4.637171 | 1.24E-08 |
| 389 | -0.05131 | 1.97E-10 | 4.63938 | 1.24E-08 |
| 390 | -0.05298 | 2.00E-10 | 4.641579 | 1.24E-08 |
| 391 | -0.05466 | 2.03E-10 | 4.643766 | 1.24E-08 |
| 392 | -0.05634 | 2.07E-10 | 4.645941 | 1.24E-08 |
| 393 | -0.05801 | 2.10E-10 | 4.648105 | 1.25E-08 |
| 394 | -0.05969 | 2.13E-10 | 4.650257 | 1.25E-08 |
| 395 | -0.06136 | 2.17E-10 | 4.652398 | 1.25E-08 |
| 396 | -0.06304 | 2.20E-10 | 4.654527 | 1.25E-08 |
| 397 | -0.06472 | 2.23E-10 | 4.656644 | 1.25E-08 |
| 398 | -0.06639 | 2.27E-10 | 4.65875 | 1.25E-08 |
| 399 | -0.06807 | 2.30E-10 | 4.660844 | 1.25E-08 |
| 400 | -0.06974 | 2.34E-10 | 4.662927 | 1.25E-08 |
| 401 | -0.07141 | 2.38E-10 | 4.664998 | 1.25E-08 |
| 402 | -0.07309 | 2.41E-10 | 4.667058 | 1.25E-08 |
| 403 | -0.07476 | 2.45E-10 | 4.669106 | 1.25E-08 |
| 404 | -0.07643 | 2.49E-10 | 4.671142 | 1.25E-08 |
| 405 | -0.07811 | 2.52E-10 | 4.673167 | 1.25E-08 |
| 406 | -0.07978 | 2.56E-10 | 4.67518 | 1.25E-08 |
| 407 | -0.08145 | 2.60E-10 | 4.677182 | 1.25E-08 |
| 408 | -0.08312 | 2.63E-10 | 4.679172 | 1.25E-08 |
| 409 | -0.0848 | 2.67E-10 | 4.68115 | 1.25E-08 |
| 410 | -0.08647 | 2.71E-10 | 4.683117 | 1.25E-08 |
| 411 | -0.08814 | 2.75E-10 | 4.685072 | 1.25E-08 |
| 412 | -0.08981 | 2.79E-10 | 4.687015 | 1.26E-08 |
| 413 | -0.09148 | 2.83E-10 | 4.688947 | 1.26E-08 |
| 414 | -0.09315 | 2.86E-10 | 4.690867 | 1.26E-08 |
| 415 | -0.09482 | 2.90E-10 | 4.692776 | 1.26E-08 |
| 416 | -0.09649 | 2.94E-10 | 4.694672 | 1.26E-08 |
| 417 | -0.09816 | 2.98E-10 | 4.696558 | 1.26E-08 |
| 418 | -0.09983 | 3.02E-10 | 4.698431 | 1.26E-08 |
| 419 | -0.10149 | 3.06E-10 | 4.700293 | 1.26E-08 |
| 420 | -0.10316 | 3.10E-10 | 4.702143 | 1.26E-08 |
| 421 | -0.10483 | 3.14E-10 | 4.703982 | 1.26E-08 |
| 422 | -0.1065 | 3.18E-10 | 4.705809 | 1.26E-08 |
| 423 | -0.10816 | 3.22E-10 | 4.707624 | 1.26E-08 |
| 424 | -0.10983 | 3.26E-10 | 4.709427 | 1.26E-08 |
| 425 | -0.11149 | 3.30E-10 | 4.711219 | 1.26E-08 |
| 426 | -0.11316 | 3.34E-10 | 4.712999 | 1.26E-08 |
| 427 | -0.11482 | 3.38E-10 | 4.714767 | 1.26E-08 |
| 428 | -0.11649 | 3.42E-10 | 4.716524 | 1.26E-08 |
| 429 | -0.11815 | 3.46E-10 | 4.718269 | 1.26E-08 |
| 430 | -0.11982 | 3.50E-10 | 4.720002 | 1.26E-08 |
| 431 | -0.12148 | 3.54E-10 | 4.721724 | 1.26E-08 |
| 432 | -0.12314 | 3.58E-10 | 4.723434 | 1.27E-08 |
| 433 | -0.12481 | 3.62E-10 | 4.725132 | 1.27E-08 |
| 434 | -0.12647 | 3.66E-10 | 4.726818 | 1.27E-08 |
| 435 | -0.12813 | 3.70E-10 | 4.728493 | 1.27E-08 |
| 436 | -0.12979 | 3.75E-10 | 4.730156 | 1.27E-08 |
| 437 | -0.13145 | 3.91E-10 | 4.731807 | 1.27E-08 |
| 438 | -0.13311 | 4.14E-10 | 4.733447 | 1.28E-08 |
| 439 | -0.13477 | 3.33E-10 | 4.735075 | 1.26E-08 |
| 440 | -0.13643 | 1.91E-10 | 4.736691 | 1.16E-08 |
| 441 | -0.13809 | 1.79E-09 | 4.738295 | 1.14E-08 |
| 442 | -0.13975 | 5.98E-09 | 4.739887 | 2.18E-08 |
| 443 | -0.14141 | 1.71E-08 | 4.741468 | 4.57E-08 |
| 444 | -0.14306 | 4.82E-08 | 4.743037 | 9.70E-08 |
| 445 | -0.14472 | 1.33E-07 | 4.744594 | 2.55E-07 |
| 446 | -0.14638 | 3.54E-07 | 4.74614 | 7.00E-07 |
| 447 | -0.14803 | 9.03E-07 | 4.747674 | 1.81E-06 |
| 448 | -0.14969 | 2.21E-06 | 4.749196 | 4.43E-06 |
| 449 | -0.15135 | 5.20E-06 | 4.750706 | 1.04E-05 |
| 450 | -0.153 | 1.18E-05 | 4.752204 | 2.35E-05 |
| 451 | -0.15465 | 2.56E-05 | 4.753691 | 5.11E-05 |
| 452 | -0.15631 | 5.33E-05 | 4.755166 | 1.07E-04 |
| 453 | -0.15796 | 1.07E-04 | 4.756629 | 2.14E-04 |
| 454 | -0.15961 | 2.06E-04 | 4.75808 | 4.11E-04 |
| 455 | -0.16127 | 3.81E-04 | 4.759519 | 7.60E-04 |
| 456 | -0.16292 | 6.76E-04 | 4.760947 | 1.35E-03 |
| 457 | -0.16457 | 1.15E-03 | 4.762363 | 2.30E-03 |
| 458 | -0.16622 | 1.89E-03 | 4.763767 | 3.77E-03 |
| 459 | -0.16787 | 2.97E-03 | 4.765159 | 5.93E-03 |
| 460 | -0.16952 | 4.48E-03 | 4.76654 | 8.95E-03 |
| 461 | -0.17117 | 6.49E-03 | 4.767909 | 1.30E-02 |
| 462 | -0.17282 | 9.03E-03 | 4.769265 | 1.80E-02 |
| 463 | -0.17447 | 1.21E-02 | 4.770611 | 2.41E-02 |
| 464 | -0.17611 | 1.55E-02 | 4.771944 | 3.09E-02 |
| 465 | -0.17776 | 1.90E-02 | 4.773265 | 0.037974 |
| 466 | -0.17941 | 0.02243 | 4.774575 | 0.044812 |
| 467 | -0.18105 | 0.025398 | 4.775873 | 0.050742 |
| 468 | -0.1827 | 0.027594 | 4.777159 | 0.055127 |
| 469 | -0.14188 | 0.028762 | 4.693588 | 0.057461 |
| 470 | 0.22954 | 0.028763 | 3.949429 | 0.057462 |
| 471 | 0.472199 | 0.027595 | 3.462263 | 0.055128 |
| 472 | 0.587361 | 0.025399 | 3.22971 | 0.05074 |
| 473 | 0.64819 | 0.022428 | 3.105675 | 0.044804 |
| 474 | 0.685247 | 0.019001 | 3.029137 | 0.037957 |
| 475 | 0.709222 | 0.015445 | 2.978744 | 0.030853 |
| 476 | 0.725224 | 0.012046 | 2.944297 | 0.024064 |
| 477 | 0.736198 | 0.009016 | 2.919915 | 0.01801 |
| 478 | 0.743923 | 0.006476 | 2.902039 | 0.012937 |
| 479 | 0.749495 | 0.004465 | 2.888482 | 0.008919 |
| 480 | 0.753599 | 0.002955 | 2.87787 | 0.005902 |
| 481 | 0.756674 | 0.001877 | 2.869326 | 0.00375 |
| 482 | 0.759006 | 0.001145 | 2.862274 | 0.002287 |
| 483 | 0.760789 | 0.000671 | 2.856327 | 0.00134 |
| 484 | 0.762155 | 0.000377 | 2.85122 | 0.000754 |
| 485 | 0.763196 | 0.000204 | 2.846766 | 0.000408 |
| 486 | 0.763979 | 0.000106 | 2.84283 | 0.000212 |
| 487 | 0.764553 | 5.29E-05 | 2.839311 | 0.000106 |
| 488 | 0.764955 | 2.54E-05 | 2.836135 | 5.07E-05 |
| 489 | 0.765214 | 1.17E-05 | 2.833244 | 2.34E-05 |
| 490 | 0.765351 | 5.18E-06 | 2.830594 | 1.04E-05 |
| 491 | 0.765384 | 2.21E-06 | 2.828149 | 4.42E-06 |
| 492 | 0.765326 | 9.09E-07 | 2.82588 | 1.80E-06 |
| 493 | 0.765189 | 3.59E-07 | 2.823766 | 7.10E-07 |
| 494 | 0.764981 | 1.35E-07 | 2.821786 | 2.75E-07 |
| 495 | 0.76471 | 4.75E-08 | 2.819926 | 1.06E-07 |
| 496 | 0.764383 | 1.72E-08 | 2.818173 | 3.68E-08 |
| 497 | 0.764004 | 7.74E-09 | 2.816516 | 6.64E-09 |
| 498 | 0.763578 | 3.80E-09 | 2.814945 | 5.00E-09 |
| 499 | 0.763108 | 2.01E-09 | 2.813454 | 7.88E-09 |
| 500 | 0.762598 | 1.90E-09 | 2.812034 | 7.82E-09 |
| 501 | 0.762051 | 2.05E-09 | 2.810682 | 7.51E-09 |
| 502 | 0.761468 | 2.08E-09 | 2.809391 | 7.44E-09 |
| 503 | 0.760853 | 2.07E-09 | 2.808157 | 7.45E-09 |
| 504 | 0.760206 | 2.07E-09 | 2.806977 | 7.46E-09 |
| 505 | 0.75953 | 2.06E-09 | 2.805847 | 7.46E-09 |
| 506 | 0.758825 | 2.06E-09 | 2.804763 | 7.46E-09 |
| 507 | 0.758094 | 2.06E-09 | 2.803723 | 7.46E-09 |
| 508 | 0.757338 | 2.06E-09 | 2.802725 | 7.45E-09 |
| 509 | 0.756557 | 2.05E-09 | 2.801765 | 7.45E-09 |
| 510 | 0.755753 | 2.04E-09 | 2.800843 | 7.47E-09 |
| 511 | 0.754926 | 2.03E-09 | 2.799955 | 7.48E-09 |
| 512 | 0.754079 | 2.04E-09 | 2.7991 | 7.46E-09 |
| 513 | 0.75321 | 2.07E-09 | 2.798277 | 7.39E-09 |
| 514 | 0.752322 | 2.07E-09 | 2.797483 | 7.38E-09 |
| 515 | 0.751415 | 2.01E-09 | 2.796717 | 7.52E-09 |
| 516 | 0.75049 | 1.94E-09 | 2.795977 | 7.64E-09 |
| 517 | 0.749547 | 2.00E-09 | 2.795263 | 7.51E-09 |
| 518 | 0.748587 | 2.15E-09 | 2.794572 | 7.19E-09 |
| 519 | 0.74761 | 2.17E-09 | 2.793905 | 7.17E-09 |
| 520 | 0.746618 | 1.97E-09 | 2.793258 | 7.60E-09 |
| 521 | 0.745611 | 1.78E-09 | 2.792632 | 7.92E-09 |
| 522 | 0.744589 | 1.93E-09 | 2.792025 | 7.63E-09 |
| 523 | 0.743553 | 2.20E-09 | 2.791437 | 7.06E-09 |
| 524 | 0.742503 | 2.22E-09 | 2.790865 | 7.04E-09 |
| 525 | 0.741439 | 1.97E-09 | 2.79031 | 7.56E-09 |
| 526 | 0.740363 | 1.80E-09 | 2.789771 | 7.85E-09 |
| 527 | 0.739275 | 1.93E-09 | 2.789246 | 7.59E-09 |
| 528 | 0.738175 | 2.09E-09 | 2.788734 | 7.24E-09 |
| 529 | 0.737063 | 2.07E-09 | 2.788236 | 7.29E-09 |
| 530 | 0.735939 | 1.95E-09 | 2.78775 | 7.52E-09 |
| 531 | 0.734805 | 1.95E-09 | 2.787276 | 7.51E-09 |
| 532 | 0.733661 | 2.03E-09 | 2.786812 | 7.34E-09 |
| 533 | 0.732506 | 2.01E-09 | 2.786359 | 7.38E-09 |
| 534 | 0.731341 | 1.90E-09 | 2.785915 | 7.60E-09 |
| 535 | 0.730167 | 1.88E-09 | 2.785481 | 7.62E-09 |
| 536 | 0.728983 | 2.00E-09 | 2.785055 | 7.37E-09 |
| 537 | 0.72779 | 2.08E-09 | 2.784636 | 7.20E-09 |
| 538 | 0.726589 | 2.00E-09 | 2.784226 | 7.37E-09 |
| 539 | 0.725379 | 1.87E-09 | 2.783822 | 7.61E-09 |
| 540 | 0.724161 | 1.86E-09 | 2.783424 | 7.61E-09 |
| 541 | 0.722934 | 1.96E-09 | 2.783032 | 7.41E-09 |
| 542 | 0.7217 | 2.01E-09 | 2.782646 | 7.30E-09 |
| 543 | 0.720458 | 1.97E-09 | 2.782266 | 7.39E-09 |
| 544 | 0.719209 | 1.91E-09 | 2.781889 | 7.49E-09 |
| 545 | 0.717952 | 1.90E-09 | 2.781517 | 7.49E-09 |
| 546 | 0.716688 | 1.93E-09 | 2.78115 | 7.44E-09 |
| 547 | 0.715418 | 1.94E-09 | 2.780785 | 7.41E-09 |
| 548 | 0.714141 | 1.93E-09 | 2.780424 | 7.42E-09 |
| 549 | 0.712857 | 1.92E-09 | 2.780066 | 7.44E-09 |
