# Supplementary material for: A new method for identifying a fault in T-connected lines based on multiscale S-transform energy entropy and an extreme learning machine
Source: PLoS One. 2019 Aug 15;14(8):e0220870. doi: 10.1371/journal.pone.0220870 (PMC6695217; doi:10.1371/journal.pone.0220870)
Supplement: S14 Table — (DOCX) [file pone.0220870.s015.docx]

**S14 Table. The partial data obtained from Fig.8 is as follows.**

| AG phase to ground short circuit occurring on transmission line BE at a distance of 250 km from O point, fault resistance of 100 Ω (fault initial angle of 60°) | | | | |
| --- | --- | --- | --- | --- |
| N-th sampling point | Original current | original current s-transformed | Current reverse traveling wave | Current reverse traveling wave s-transformed |
| 101 | 0.579935 | 1.56E-09 | 3.397366 | 9.11E-09 |
| 102 | 0.578379 | 1.55E-09 | 3.402461 | 9.12E-09 |
| 103 | 0.576821 | 1.60E-09 | 3.407548 | 9.14E-09 |
| 104 | 0.575262 | 1.70E-09 | 3.412626 | 9.15E-09 |
| 105 | 0.573702 | 1.46E-09 | 3.417695 | 9.16E-09 |
| 106 | 0.57214 | 6.96E-10 | 3.422756 | 9.18E-09 |
| 107 | 0.570577 | 6.85E-09 | 3.427808 | 9.19E-09 |
| 108 | 0.569012 | 2.35E-08 | 3.432852 | 9.17E-09 |
| 109 | 0.567446 | 6.76E-08 | 3.437887 | 9.14E-09 |
| 110 | 0.565879 | 1.89E-07 | 3.442914 | 9.42E-09 |
| 111 | 0.56431 | 5.20E-07 | 3.447932 | 1.02E-08 |
| 112 | 0.56274 | 1.38E-06 | 3.452941 | 9.89E-09 |
| 113 | 0.561168 | 3.49E-06 | 3.457942 | 4.49E-09 |
| 114 | 0.559595 | 8.52E-06 | 3.462934 | 1.12E-08 |
| 115 | 0.558021 | 1.99E-05 | 3.467918 | 4.19E-08 |
| 116 | 0.556445 | 4.49E-05 | 3.472893 | 9.28E-08 |
| 117 | 0.554868 | 9.71E-05 | 3.477859 | 1.83E-07 |
| 118 | 0.55329 | 2.02E-04 | 3.482816 | 3.69E-07 |
| 119 | 0.55171 | 4.02E-04 | 3.487765 | 7.47E-07 |
| 120 | 0.550129 | 7.71E-04 | 3.492705 | 1.45E-06 |
| 121 | 0.548547 | 1.42E-03 | 3.497637 | 2.69E-06 |
| 122 | 0.546963 | 2.51E-03 | 3.502559 | 4.77E-06 |
| 123 | 0.545378 | 4.26E-03 | 3.507473 | 8.12E-06 |
| 124 | 0.543792 | 6.95E-03 | 3.512378 | 1.33E-05 |
| 125 | 0.542204 | 1.09E-02 | 3.517275 | 2.10E-05 |
| 126 | 0.540615 | 1.64E-02 | 3.522162 | 3.18E-05 |
| 127 | 0.539025 | 2.36E-02 | 3.527041 | 4.62E-05 |
| 128 | 0.537433 | 3.27E-02 | 3.531911 | 6.45E-05 |
| 129 | 0.53584 | 4.35E-02 | 3.536772 | 8.65E-05 |
| 130 | 0.534246 | 5.55E-02 | 3.541625 | 1.11E-04 |
| 131 | 0.532651 | 6.80E-02 | 3.546468 | 1.38E-04 |
| 132 | 0.531054 | 7.99E-02 | 3.551303 | 1.64E-04 |
| 133 | 0.529456 | 9.01E-02 | 3.556128 | 1.86E-04 |
| 134 | 0.527856 | 9.76E-02 | 3.560945 | 2.04E-04 |
| 135 | 0.306933 | 1.01E-01 | 3.566238 | 2.14E-04 |
| 136 | -1.07192 | 1.01E-01 | 3.573254 | 2.16E-04 |
| 137 | -1.10763 | 9.67E-02 | 3.577097 | 2.08E-04 |
| 138 | -1.11875 | 8.88E-02 | 3.580972 | 1.93E-04 |
| 139 | -1.12206 | 7.82E-02 | 3.584915 | 1.72E-04 |
| 140 | -1.12251 | 6.61E-02 | 3.58893 | 1.47E-04 |
| 141 | -1.12204 | 5.36E-02 | 3.593011 | 1.20E-04 |
| 142 | -1.12138 | 4.17E-02 | 3.59715 | 9.42E-05 |
| 143 | -1.1208 | 3.12E-02 | 3.601341 | 7.09E-05 |
| 144 | -1.12038 | 2.23E-02 | 3.605575 | 5.12E-05 |
| 145 | -1.12015 | 1.54E-02 | 3.609847 | 3.55E-05 |
| 146 | -1.12012 | 1.02E-02 | 3.614151 | 2.36E-05 |
| 147 | -1.12028 | 6.44E-03 | 3.618482 | 1.51E-05 |
| 148 | -1.12061 | 3.92E-03 | 3.622834 | 9.22E-06 |
| 149 | -1.12109 | 2.29E-03 | 3.627205 | 5.40E-06 |
| 150 | -1.12173 | 1.28E-03 | 3.631591 | 3.04E-06 |
| 151 | -1.1225 | 6.92E-04 | 3.635989 | 1.66E-06 |
| 152 | -1.1234 | 3.58E-04 | 3.640397 | 8.67E-07 |
| 153 | -1.1244 | 1.78E-04 | 3.644811 | 4.29E-07 |
| 154 | -1.12551 | 8.48E-05 | 3.649231 | 1.96E-07 |
| 155 | -1.1267 | 3.88E-05 | 3.653653 | 8.64E-08 |
| 156 | -1.12798 | 1.71E-05 | 3.658077 | 4.55E-08 |
| 157 | -1.12932 | 7.22E-06 | 3.662502 | 2.73E-08 |
| 158 | -1.13073 | 2.93E-06 | 3.666926 | 1.40E-08 |
| 159 | -1.1322 | 1.14E-06 | 3.671348 | 7.89E-09 |
| 160 | -1.13371 | 4.25E-07 | 3.675767 | 8.98E-09 |
| 161 | -1.13526 | 1.54E-07 | 3.680182 | 9.95E-09 |
| 162 | -1.13686 | 5.61E-08 | 3.684594 | 1.00E-08 |
| 163 | -1.13848 | 1.95E-08 | 3.689 | 9.91E-09 |
| 164 | -1.14014 | 4.42E-09 | 3.693401 | 9.89E-09 |
| 165 | -1.14182 | 1.55E-09 | 3.697796 | 9.91E-09 |
| 166 | -1.14352 | 3.11E-09 | 3.702185 | 9.92E-09 |
| 167 | -1.14524 | 3.21E-09 | 3.706567 | 9.93E-09 |
| 168 | -1.14697 | 3.10E-09 | 3.710942 | 9.95E-09 |
| 169 | -1.14872 | 3.07E-09 | 3.71531 | 9.96E-09 |
| 170 | -1.15048 | 3.08E-09 | 3.71967 | 9.97E-09 |
| 171 | -1.15225 | 3.09E-09 | 3.724023 | 9.98E-09 |
| 172 | -1.15403 | 3.09E-09 | 3.728367 | 9.99E-09 |
| 173 | -1.15581 | 3.10E-09 | 3.732704 | 1.00E-08 |
| 174 | -1.1576 | 3.10E-09 | 3.737032 | 1.00E-08 |
| 175 | -1.1594 | 3.11E-09 | 3.741352 | 1.00E-08 |
| 176 | -1.16119 | 3.11E-09 | 3.745664 | 1.00E-08 |
| 177 | -1.16299 | 3.12E-09 | 3.749967 | 1.01E-08 |
| 178 | -1.16479 | 3.12E-09 | 3.754262 | 1.01E-08 |
| 179 | -1.16659 | 3.13E-09 | 3.758547 | 1.01E-08 |
| 180 | -1.16839 | 3.13E-09 | 3.762824 | 1.01E-08 |
| 181 | -1.1702 | 3.14E-09 | 3.767093 | 1.01E-08 |
| 182 | -1.172 | 3.14E-09 | 3.771352 | 1.01E-08 |
| 183 | -1.1738 | 3.15E-09 | 3.775602 | 1.01E-08 |
| 184 | -1.1756 | 3.15E-09 | 3.779843 | 1.01E-08 |
| 185 | -1.17739 | 3.16E-09 | 3.784076 | 1.01E-08 |
| 186 | -1.17919 | 3.16E-09 | 3.788299 | 1.02E-08 |
| 187 | -1.18098 | 3.18E-09 | 3.792513 | 1.02E-08 |
| 188 | -1.18277 | 3.18E-09 | 3.796718 | 1.02E-08 |
| 189 | -1.18456 | 3.15E-09 | 3.800913 | 1.02E-08 |
| 190 | -1.18635 | 3.14E-09 | 3.8051 | 1.02E-08 |
| 191 | -1.18814 | 3.21E-09 | 3.809277 | 1.02E-08 |
| 192 | -1.18992 | 3.30E-09 | 3.813445 | 1.02E-08 |
| 193 | -1.1917 | 3.25E-09 | 3.817604 | 1.02E-08 |
| 194 | -1.19347 | 3.02E-09 | 3.821753 | 1.02E-08 |
| 195 | -1.19525 | 2.96E-09 | 3.825893 | 1.03E-08 |
| 196 | -1.19702 | 3.34E-09 | 3.830024 | 1.03E-08 |
| 197 | -1.19879 | 3.71E-09 | 3.834145 | 1.03E-08 |
| 198 | -1.20055 | 3.47E-09 | 3.838257 | 1.03E-08 |
| 199 | -1.20232 | 2.73E-09 | 3.84236 | 1.03E-08 |
| 200 | -1.20408 | 2.63E-09 | 3.846453 | 1.03E-08 |
| 201 | -1.20583 | 3.52E-09 | 3.850537 | 1.03E-08 |
| 202 | -1.20759 | 4.05E-09 | 3.854612 | 1.03E-08 |
| 203 | -1.20934 | 3.61E-09 | 3.858677 | 1.03E-08 |
| 204 | -1.21109 | 2.71E-09 | 3.862732 | 1.04E-08 |
| 205 | -1.21283 | 2.70E-09 | 3.866779 | 1.04E-08 |
| 206 | -1.21458 | 3.44E-09 | 3.870815 | 1.04E-08 |
| 207 | -1.21632 | 3.75E-09 | 3.874842 | 1.04E-08 |
| 208 | -1.21805 | 3.44E-09 | 3.87886 | 1.04E-08 |
| 209 | -1.21979 | 3.05E-09 | 3.882868 | 1.04E-08 |
| 210 | -1.22152 | 3.08E-09 | 3.886867 | 1.04E-08 |
| 211 | -1.22325 | 3.32E-09 | 3.890856 | 1.04E-08 |
| 212 | -1.22497 | 3.39E-09 | 3.894835 | 1.04E-08 |
| 213 | -1.2267 | 3.32E-09 | 3.898805 | 1.04E-08 |
| 214 | -1.22842 | 3.26E-09 | 3.902766 | 1.05E-08 |
| 215 | -1.23014 | 3.27E-09 | 3.906716 | 1.05E-08 |
| 216 | -1.23185 | 3.31E-09 | 3.910658 | 1.05E-08 |
| 217 | -1.23356 | 3.32E-09 | 3.914589 | 1.05E-08 |
| 218 | -1.23527 | 3.31E-09 | 3.918511 | 1.05E-08 |
| 219 | -1.23698 | 3.31E-09 | 3.922424 | 1.05E-08 |
| 220 | -1.23868 | 3.32E-09 | 3.926326 | 1.05E-08 |
| 221 | -1.24038 | 3.32E-09 | 3.930219 | 1.05E-08 |
| 222 | -1.24208 | 3.33E-09 | 3.934103 | 1.05E-08 |
| 223 | -1.24378 | 3.33E-09 | 3.937976 | 1.06E-08 |
| 224 | -1.24547 | 3.34E-09 | 3.94184 | 1.06E-08 |
| 225 | -1.24716 | 3.34E-09 | 3.945695 | 1.06E-08 |
| 226 | -1.24885 | 3.35E-09 | 3.949539 | 1.06E-08 |
| 227 | -1.25054 | 3.35E-09 | 3.953374 | 1.06E-08 |
| 228 | -1.25222 | 3.36E-09 | 3.9572 | 1.06E-08 |
| 229 | -1.2539 | 3.36E-09 | 3.961015 | 1.06E-08 |
| 230 | -1.25558 | 3.37E-09 | 3.964821 | 1.06E-08 |
| 231 | -1.25725 | 3.37E-09 | 3.968617 | 1.06E-08 |
| 232 | -1.25892 | 3.37E-09 | 3.972403 | 1.06E-08 |
| 233 | -1.26059 | 3.38E-09 | 3.976179 | 1.07E-08 |
| 234 | -1.26226 | 3.38E-09 | 3.979946 | 1.07E-08 |
| 235 | -1.26392 | 3.39E-09 | 3.983703 | 1.07E-08 |
| 236 | -1.26558 | 3.40E-09 | 3.98745 | 1.07E-08 |
| 237 | -1.26724 | 3.44E-09 | 3.991187 | 1.07E-08 |
| 238 | -1.2689 | 3.43E-09 | 3.994914 | 1.07E-08 |
| 239 | -1.27055 | 2.89E-09 | 3.998632 | 1.07E-08 |
| 240 | -1.2722 | 2.41E-09 | 4.00234 | 1.07E-08 |
| 241 | -1.27385 | 8.10E-09 | 4.006038 | 1.07E-08 |
| 242 | -1.27549 | 2.18E-08 | 4.009726 | 1.08E-08 |
| 243 | -1.27714 | 5.41E-08 | 4.013404 | 1.08E-08 |
| 244 | -1.27878 | 1.45E-07 | 4.017072 | 1.05E-08 |
| 245 | -1.28041 | 3.94E-07 | 4.02073 | 1.02E-08 |
| 246 | -1.28205 | 1.02E-06 | 4.024379 | 1.19E-08 |
| 247 | -1.28368 | 2.52E-06 | 4.028017 | 1.60E-08 |
| 248 | -1.28531 | 5.98E-06 | 4.031646 | 1.81E-08 |
| 249 | -1.28694 | 1.36E-05 | 4.035264 | 2.00E-08 |
| 250 | -1.28856 | 2.98E-05 | 4.038873 | 5.42E-08 |
| 251 | -1.29018 | 6.28E-05 | 4.042472 | 1.35E-07 |
| 252 | -1.2918 | 1.27E-04 | 4.04606 | 2.77E-07 |
| 253 | -1.29342 | 2.46E-04 | 4.049639 | 5.23E-07 |
| 254 | -1.29503 | 4.59E-04 | 4.053208 | 9.64E-07 |
| 255 | -1.29664 | 8.22E-04 | 4.056767 | 1.74E-06 |
| 256 | -1.29825 | 1.41E-03 | 4.060316 | 3.03E-06 |
| 257 | -1.29986 | 2.33E-03 | 4.063855 | 5.03E-06 |
| 258 | -1.30146 | 3.70E-03 | 4.067383 | 8.02E-06 |
| 259 | -1.30306 | 5.64E-03 | 4.070902 | 1.23E-05 |
| 260 | -1.30466 | 8.25E-03 | 4.074411 | 1.81E-05 |
| 261 | -1.30625 | 1.16E-02 | 4.07791 | 2.56E-05 |
| 262 | -1.30784 | 1.56E-02 | 4.081398 | 3.47E-05 |
| 263 | -1.30943 | 2.02E-02 | 4.084877 | 4.53E-05 |
| 264 | -1.31102 | 2.51E-02 | 4.088345 | 5.67E-05 |
| 265 | -1.3126 | 2.99E-02 | 4.091804 | 6.82E-05 |
| 266 | -1.31418 | 3.42E-02 | 4.095252 | 7.88E-05 |
| 267 | -1.31576 | 3.76E-02 | 4.09869 | 8.74E-05 |
| 268 | -1.31734 | 3.96E-02 | 4.102118 | 9.29E-05 |
| 269 | -0.89452 | 4.01E-02 | 4.104654 | 9.49E-05 |
| 270 | -0.55333 | 3.89E-02 | 4.107719 | 9.29E-05 |
| 271 | -0.4767 | 3.63E-02 | 4.111448 | 8.74E-05 |
| 272 | -0.45165 | 3.24E-02 | 4.115263 | 7.88E-05 |
| 273 | -0.44803 | 2.78E-02 | 4.119076 | 6.82E-05 |
| 274 | -0.45632 | 2.29E-02 | 4.122857 | 5.66E-05 |
| 275 | -0.47185 | 1.81E-02 | 4.126597 | 4.51E-05 |
| 276 | -0.49178 | 1.38E-02 | 4.130291 | 3.44E-05 |
| 277 | -0.51416 | 1.00E-02 | 4.133937 | 2.53E-05 |
| 278 | -0.53762 | 7.02E-03 | 4.137535 | 1.78E-05 |
| 279 | -0.56123 | 4.71E-03 | 4.141089 | 1.20E-05 |
| 280 | -0.58434 | 3.04E-03 | 4.1446 | 7.77E-06 |
| 281 | -0.6065 | 1.88E-03 | 4.148072 | 4.83E-06 |
| 282 | -0.62745 | 1.12E-03 | 4.151508 | 2.89E-06 |
| 283 | -0.64705 | 6.41E-04 | 4.154911 | 1.67E-06 |
| 284 | -0.66521 | 3.52E-04 | 4.158285 | 9.17E-07 |
| 285 | -0.68194 | 1.85E-04 | 4.161631 | 4.75E-07 |
| 286 | -0.69726 | 9.40E-05 | 4.164953 | 2.34E-07 |
| 287 | -0.71125 | 4.57E-05 | 4.168252 | 1.19E-07 |
| 288 | -0.72397 | 2.14E-05 | 4.171532 | 6.62E-08 |
| 289 | -0.73553 | 9.61E-06 | 4.174793 | 3.38E-08 |
| 290 | -0.746 | 4.15E-06 | 4.178037 | 1.18E-08 |
| 291 | -0.7555 | 1.72E-06 | 4.181266 | 6.72E-09 |
| 292 | -0.7641 | 6.85E-07 | 4.18448 | 1.08E-08 |
| 293 | -0.7719 | 2.64E-07 | 4.187681 | 1.18E-08 |
| 294 | -0.77898 | 9.87E-08 | 4.190869 | 1.14E-08 |
| 295 | -0.78541 | 3.42E-08 | 4.194045 | 1.12E-08 |
| 296 | -0.79127 | 9.52E-09 | 4.19721 | 1.12E-08 |
| 297 | -0.79662 | 3.00E-09 | 4.200363 | 1.12E-08 |
| 298 | -0.80151 | 3.03E-09 | 4.203506 | 1.13E-08 |
| 299 | -0.80601 | 2.58E-09 | 4.206639 | 1.13E-08 |
| 300 | -0.81015 | 2.24E-09 | 4.209761 | 1.13E-08 |
| 301 | -0.81398 | 2.21E-09 | 4.212873 | 1.13E-08 |
| 302 | -0.81754 | 2.23E-09 | 4.215976 | 1.13E-08 |
| 303 | -0.82086 | 2.24E-09 | 4.219068 | 1.13E-08 |
| 304 | -0.82397 | 2.24E-09 | 4.222151 | 1.13E-08 |
| 305 | -0.82689 | 2.24E-09 | 4.225224 | 1.13E-08 |
| 306 | -0.82965 | 2.25E-09 | 4.228288 | 1.13E-08 |
| 307 | -0.83228 | 2.25E-09 | 4.231341 | 1.13E-08 |
| 308 | -0.83477 | 2.26E-09 | 4.234385 | 1.13E-08 |
| 309 | -0.83716 | 2.26E-09 | 4.237419 | 1.14E-08 |
| 310 | -0.83946 | 2.27E-09 | 4.240443 | 1.14E-08 |
| 311 | -0.84167 | 2.27E-09 | 4.243457 | 1.14E-08 |
| 312 | -0.84381 | 2.27E-09 | 4.246461 | 1.14E-08 |
| 313 | -0.84589 | 2.28E-09 | 4.249455 | 1.14E-08 |
| 314 | -0.84791 | 2.28E-09 | 4.25244 | 1.14E-08 |
| 315 | -0.84989 | 2.29E-09 | 4.255414 | 1.14E-08 |
| 316 | -0.85182 | 2.29E-09 | 4.258378 | 1.14E-08 |
| 317 | -0.85371 | 2.30E-09 | 4.261332 | 1.14E-08 |
| 318 | -0.85558 | 2.30E-09 | 4.264276 | 1.14E-08 |
| 319 | -0.85741 | 2.30E-09 | 4.267209 | 1.14E-08 |
| 320 | -0.85922 | 2.31E-09 | 4.270133 | 1.14E-08 |
| 321 | -0.86101 | 2.31E-09 | 4.273046 | 1.14E-08 |
| 322 | -0.86278 | 2.32E-09 | 4.275948 | 1.15E-08 |
| 323 | -0.86453 | 2.32E-09 | 4.27884 | 1.15E-08 |
| 324 | -0.86627 | 2.32E-09 | 4.281722 | 1.15E-08 |
| 325 | -0.86799 | 2.33E-09 | 4.284594 | 1.15E-08 |
| 326 | -0.8697 | 2.36E-09 | 4.287454 | 1.15E-08 |
| 327 | -0.8714 | 2.35E-09 | 4.290305 | 1.15E-08 |
| 328 | -0.87309 | 2.31E-09 | 4.293145 | 1.15E-08 |
| 329 | -0.87477 | 2.29E-09 | 4.295974 | 1.15E-08 |
| 330 | -0.87645 | 2.37E-09 | 4.298793 | 1.15E-08 |
| 331 | -0.87812 | 2.46E-09 | 4.301601 | 1.15E-08 |
| 332 | -0.87978 | 2.42E-09 | 4.304399 | 1.15E-08 |
| 333 | -0.88143 | 2.27E-09 | 4.307186 | 1.15E-08 |
| 334 | -0.88308 | 2.24E-09 | 4.309962 | 1.15E-08 |
| 335 | -0.88473 | 2.40E-09 | 4.312728 | 1.16E-08 |
| 336 | -0.88637 | 2.54E-09 | 4.315483 | 1.16E-08 |
| 337 | -0.88801 | 2.47E-09 | 4.318228 | 1.16E-08 |
| 338 | -0.88964 | 2.29E-09 | 4.320961 | 1.16E-08 |
| 339 | -0.89127 | 2.27E-09 | 4.323684 | 1.16E-08 |
| 340 | -0.89289 | 2.41E-09 | 4.326397 | 1.16E-08 |
| 341 | -0.89451 | 2.49E-09 | 4.329098 | 1.16E-08 |
| 342 | -0.89613 | 2.45E-09 | 4.331789 | 1.16E-08 |
| 343 | -0.89775 | 2.37E-09 | 4.334469 | 1.16E-08 |
| 344 | -0.89936 | 2.37E-09 | 4.337138 | 1.16E-08 |
| 345 | -0.90097 | 2.42E-09 | 4.339797 | 1.16E-08 |
| 346 | -0.90257 | 2.44E-09 | 4.342445 | 1.16E-08 |
| 347 | -0.90418 | 2.43E-09 | 4.345082 | 1.16E-08 |
| 348 | -0.90578 | 2.43E-09 | 4.347708 | 1.16E-08 |
| 349 | -0.90738 | 2.43E-09 | 4.350323 | 1.17E-08 |
| 350 | -0.90897 | 2.44E-09 | 4.352928 | 1.17E-08 |
| 351 | -0.91056 | 2.44E-09 | 4.355522 | 1.17E-08 |
| 352 | -0.91215 | 2.45E-09 | 4.358105 | 1.17E-08 |
| 353 | -0.91374 | 2.45E-09 | 4.360677 | 1.17E-08 |
| 354 | -0.91533 | 2.46E-09 | 4.363238 | 1.17E-08 |
| 355 | -0.91691 | 2.46E-09 | 4.365788 | 1.17E-08 |
| 356 | -0.91849 | 2.46E-09 | 4.368328 | 1.17E-08 |
| 357 | -0.92006 | 2.47E-09 | 4.370857 | 1.17E-08 |
| 358 | -0.92164 | 2.47E-09 | 4.373375 | 1.17E-08 |
| 359 | -0.92321 | 2.48E-09 | 4.375882 | 1.17E-08 |
| 360 | -0.92478 | 2.48E-09 | 4.378378 | 1.17E-08 |
| 361 | -0.92635 | 2.48E-09 | 4.380863 | 1.17E-08 |
| 362 | -0.92791 | 2.49E-09 | 4.383337 | 1.17E-08 |
| 363 | -0.92948 | 2.49E-09 | 4.385801 | 1.17E-08 |
| 364 | -0.93104 | 2.50E-09 | 4.388253 | 1.18E-08 |
| 365 | -0.93259 | 2.50E-09 | 4.390695 | 1.18E-08 |
| 366 | -0.93415 | 2.51E-09 | 4.393126 | 1.18E-08 |
| 367 | -0.9357 | 2.51E-09 | 4.395545 | 1.18E-08 |
| 368 | -0.93725 | 2.51E-09 | 4.397954 | 1.18E-08 |
| 369 | -0.9388 | 2.52E-09 | 4.400352 | 1.18E-08 |
| 370 | -0.94034 | 2.52E-09 | 4.402739 | 1.18E-08 |
| 371 | -0.94188 | 2.52E-09 | 4.405115 | 1.18E-08 |
| 372 | -0.94342 | 2.53E-09 | 4.407481 | 1.18E-08 |
| 373 | -0.94496 | 2.62E-09 | 4.409835 | 1.24E-08 |
| 374 | -0.9465 | 2.73E-09 | 4.412178 | 1.31E-08 |
| 375 | -0.94803 | 2.23E-09 | 4.41451 | 1.00E-08 |
| 376 | -0.94956 | 5.15E-10 | 4.416831 | 6.98E-09 |
| 377 | -0.95109 | 7.73E-09 | 4.419142 | 5.20E-08 |
| 378 | -0.95261 | 2.36E-08 | 4.421441 | 1.55E-07 |
| 379 | -0.95413 | 5.88E-08 | 4.42373 | 3.94E-07 |
| 380 | -0.95565 | 1.45E-07 | 4.426007 | 9.82E-07 |
| 381 | -0.95717 | 3.56E-07 | 4.428273 | 2.42E-06 |
| 382 | -0.95869 | 8.46E-07 | 4.430529 | 5.73E-06 |
| 383 | -0.9602 | 1.92E-06 | 4.432773 | 1.30E-05 |
| 384 | -0.96171 | 4.19E-06 | 4.435006 | 2.84E-05 |
| 385 | -0.96321 | 8.77E-06 | 4.437229 | 5.95E-05 |
| 386 | -0.96472 | 1.76E-05 | 4.43944 | 0.00012 |
| 387 | -0.96622 | 3.41E-05 | 4.44164 | 0.000231 |
| 388 | -0.96772 | 6.32E-05 | 4.44383 | 0.000429 |
| 389 | -0.96922 | 0.000112 | 4.446008 | 0.000764 |
| 390 | -0.97071 | 0.000192 | 4.448175 | 0.001308 |
| 391 | -0.9722 | 0.000315 | 4.450331 | 0.002148 |
| 392 | -0.97369 | 0.000496 | 4.452476 | 0.003389 |
| 393 | -0.97518 | 0.00075 | 4.454611 | 0.005132 |
| 394 | -0.97666 | 0.001088 | 4.456734 | 0.007461 |
| 395 | -0.97814 | 0.001515 | 4.458846 | 0.010411 |
| 396 | -0.97962 | 0.002023 | 4.460946 | 0.013945 |
| 397 | -0.9811 | 0.002592 | 4.463036 | 0.017926 |
| 398 | -0.98257 | 0.003186 | 4.465115 | 0.022116 |
| 399 | -0.98404 | 0.003755 | 4.467183 | 0.026183 |
| 400 | -0.98551 | 0.004245 | 4.469239 | 0.029745 |
| 401 | -0.98697 | 0.004601 | 4.471285 | 0.032423 |
| 402 | -0.98958 | 0.004781 | 4.492467 | 0.033912 |
| 403 | -1.05468 | 0.004764 | 4.916228 | 0.034031 |
| 404 | -1.09426 | 0.00455 | 5.193581 | 0.032766 |
| 405 | -1.11392 | 0.004165 | 5.307705 | 0.030269 |
| 406 | -1.12494 | 0.003655 | 5.366086 | 0.02683 |
| 407 | -1.13042 | 0.003074 | 5.40249 | 0.022819 |
| 408 | -1.13179 | 0.002478 | 5.426695 | 0.018623 |
| 409 | -1.13065 | 0.001915 | 5.443535 | 0.014585 |
| 410 | -1.12821 | 0.001419 | 5.455837 | 0.010963 |
| 411 | -1.1253 | 0.001008 | 5.465279 | 0.007909 |
| 412 | -1.12245 | 0.000687 | 5.472873 | 0.005477 |
| 413 | -1.11997 | 0.000449 | 5.479241 | 0.003642 |
| 414 | -1.11808 | 0.000282 | 5.484772 | 0.002325 |
| 415 | -1.11686 | 0.00017 | 5.489713 | 0.001425 |
| 416 | -1.11635 | 9.85E-05 | 5.494227 | 0.000839 |
| 417 | -1.11655 | 5.49E-05 | 5.498424 | 0.000475 |
| 418 | -1.11743 | 2.95E-05 | 5.502375 | 0.000258 |
| 419 | -1.11893 | 1.52E-05 | 5.506133 | 0.000135 |
| 420 | -1.12099 | 7.59E-06 | 5.509735 | 6.76E-05 |
| 421 | -1.12353 | 3.65E-06 | 5.513205 | 3.26E-05 |
| 422 | -1.12648 | 1.70E-06 | 5.516564 | 1.51E-05 |
| 423 | -1.12977 | 7.64E-07 | 5.519827 | 6.72E-06 |
| 424 | -1.13334 | 3.36E-07 | 5.523005 | 2.89E-06 |
| 425 | -1.13711 | 1.44E-07 | 5.526107 | 1.21E-06 |
| 426 | -1.14105 | 5.72E-08 | 5.529139 | 4.80E-07 |
| 427 | -1.14508 | 1.92E-08 | 5.532108 | 1.71E-07 |
| 428 | -1.14917 | 7.34E-09 | 5.535018 | 5.25E-08 |
| 429 | -1.15329 | 6.05E-09 | 5.537874 | 2.76E-08 |
| 430 | -1.15739 | 4.63E-09 | 5.540678 | 2.25E-08 |
| 431 | -1.16145 | 3.53E-09 | 5.543433 | 1.67E-08 |
| 432 | -1.16546 | 3.34E-09 | 5.546143 | 1.45E-08 |
| 433 | -1.16938 | 3.41E-09 | 5.548809 | 1.46E-08 |
| 434 | -1.17321 | 3.43E-09 | 5.551433 | 1.49E-08 |
| 435 | -1.17694 | 3.41E-09 | 5.554017 | 1.49E-08 |
| 436 | -1.18056 | 3.39E-09 | 5.556562 | 1.49E-08 |
| 437 | -1.18406 | 3.37E-09 | 5.55907 | 1.49E-08 |
| 438 | -1.18745 | 3.35E-09 | 5.561543 | 1.49E-08 |
| 439 | -1.19073 | 3.36E-09 | 5.56398 | 1.49E-08 |
| 440 | -1.19389 | 3.46E-09 | 5.566384 | 1.49E-08 |
| 441 | -1.19693 | 3.50E-09 | 5.568755 | 1.49E-08 |
| 442 | -1.19987 | 2.63E-09 | 5.571094 | 1.49E-08 |
| 443 | -1.2027 | 1.51E-09 | 5.573402 | 1.50E-08 |
| 444 | -1.20543 | 1.06E-08 | 5.57568 | 1.50E-08 |
| 445 | -1.20807 | 3.00E-08 | 5.577929 | 1.50E-08 |
| 446 | -1.21061 | 7.29E-08 | 5.580149 | 1.50E-08 |
| 447 | -1.21307 | 1.79E-07 | 5.582342 | 1.46E-08 |
| 448 | -1.21544 | 4.39E-07 | 5.584507 | 1.42E-08 |
| 449 | -1.21775 | 1.04E-06 | 5.586645 | 1.55E-08 |
| 450 | -1.21997 | 2.35E-06 | 5.588756 | 1.96E-08 |
| 451 | -1.22214 | 5.10E-06 | 5.590843 | 2.23E-08 |
| 452 | -1.22424 | 1.06E-05 | 5.592904 | 1.76E-08 |
| 453 | -1.22628 | 2.13E-05 | 5.59494 | 2.88E-08 |
| 454 | -1.22828 | 4.09E-05 | 5.596952 | 8.30E-08 |
| 455 | -1.23022 | 7.56E-05 | 5.59894 | 1.68E-07 |
| 456 | -1.23212 | 0.000134 | 5.600904 | 2.86E-07 |
| 457 | -1.23397 | 0.000229 | 5.602846 | 4.66E-07 |
| 458 | -1.23579 | 0.000374 | 5.604765 | 7.62E-07 |
| 459 | -1.23757 | 0.000588 | 5.606661 | 1.23E-06 |
| 460 | -1.23932 | 0.000888 | 5.608535 | 1.88E-06 |
| 461 | -1.24104 | 0.001286 | 5.610388 | 2.74E-06 |
| 462 | -1.24274 | 0.001788 | 5.612219 | 3.82E-06 |
| 463 | -1.2444 | 0.002387 | 5.614029 | 5.14E-06 |
| 464 | -1.24604 | 0.003058 | 5.615818 | 6.65E-06 |
| 465 | -1.24766 | 0.00376 | 5.617586 | 8.27E-06 |
| 466 | -1.24926 | 0.004437 | 5.619334 | 9.86E-06 |
| 467 | -1.25085 | 0.005023 | 5.621062 | 1.13E-05 |
| 468 | -1.25241 | 0.005457 | 5.62277 | 1.24E-05 |
| 469 | -1.26398 | 0.005688 | 5.624479 | 1.30E-05 |
| 470 | -1.3389 | 0.005688 | 5.626284 | 1.32E-05 |
| 471 | -1.38955 | 0.005456 | 5.627971 | 1.28E-05 |
| 472 | -1.41394 | 0.005022 | 5.629563 | 1.19E-05 |
| 473 | -1.42763 | 0.004434 | 5.63111 | 1.06E-05 |
| 474 | -1.43645 | 0.003756 | 5.63263 | 9.04E-06 |
| 475 | -1.44253 | 0.003053 | 5.63413 | 7.42E-06 |
| 476 | -1.44697 | 0.00238 | 5.635614 | 5.85E-06 |
| 477 | -1.45041 | 0.001781 | 5.637083 | 4.41E-06 |
| 478 | -1.45324 | 0.001279 | 5.638539 | 3.18E-06 |
| 479 | -1.45569 | 0.000881 | 5.639981 | 2.20E-06 |
| 480 | -1.45794 | 0.000583 | 5.64141 | 1.47E-06 |
| 481 | -1.46011 | 0.00037 | 5.642825 | 9.51E-07 |
| 482 | -1.46231 | 0.000225 | 5.644226 | 5.88E-07 |
| 483 | -1.46463 | 0.000132 | 5.645614 | 3.36E-07 |
| 484 | -1.46712 | 7.42E-05 | 5.646987 | 1.76E-07 |
| 485 | -1.46984 | 4.01E-05 | 5.648345 | 9.61E-08 |
| 486 | -1.47281 | 2.09E-05 | 5.649689 | 6.40E-08 |
| 487 | -1.47604 | 1.04E-05 | 5.651018 | 4.20E-08 |
| 488 | -1.47952 | 5.01E-06 | 5.652331 | 2.13E-08 |
| 489 | -1.48323 | 2.31E-06 | 5.653628 | 1.03E-08 |
| 490 | -1.48715 | 1.02E-06 | 5.654909 | 1.32E-08 |
| 491 | -1.49124 | 4.38E-07 | 5.656174 | 1.55E-08 |
| 492 | -1.49547 | 1.83E-07 | 5.657423 | 1.56E-08 |
| 493 | -1.49979 | 7.20E-08 | 5.658655 | 1.52E-08 |
| 494 | -1.50419 | 2.36E-08 | 5.659871 | 1.51E-08 |
| 495 | -1.50861 | 5.37E-09 | 5.66107 | 1.51E-08 |
| 496 | -1.51305 | 5.29E-09 | 5.662252 | 1.52E-08 |
| 497 | -1.51747 | 5.34E-09 | 5.663417 | 1.52E-08 |
| 498 | -1.52185 | 4.55E-09 | 5.664566 | 1.52E-08 |
| 499 | -1.52619 | 4.25E-09 | 5.665698 | 1.52E-08 |
| 500 | -1.53045 | 4.26E-09 | 5.666814 | 1.52E-08 |
