# Supplementary material for: A new method for identifying a fault in T-connected lines based on multiscale S-transform energy entropy and an extreme learning machine
Source: PLoS One. 2019 Aug 15;14(8):e0220870. doi: 10.1371/journal.pone.0220870 (PMC6695217; doi:10.1371/journal.pone.0220870)
Supplement: S15 Table — (DOCX) [file pone.0220870.s016.docx]

**S15 Table. The partial data obtained from Fig.9 is as follows.**

| AG phase to ground short circuit occurring on transmission line BE at a distance of 250 km from O point, fault resistance of 100 Ω (fault initial angle of 60°) | | | | |
| --- | --- | --- | --- | --- |
| N-th sampling point | Original current | original current s-transformed | Current reverse traveling wave | Current reverse traveling wave s-transformed |
| 301 | 0.421284 | 1.14E-09 | 4.098563 | 1.10E-08 |
| 302 | 0.419771 | 1.13E-09 | 4.101456 | 1.10E-08 |
| 303 | 0.418258 | 1.13E-09 | 4.104339 | 1.10E-08 |
| 304 | 0.416743 | 1.12E-09 | 4.107211 | 1.10E-08 |
| 305 | 0.415228 | 1.12E-09 | 4.110073 | 1.10E-08 |
| 306 | 0.413712 | 1.12E-09 | 4.112925 | 1.10E-08 |
| 307 | 0.412195 | 1.11E-09 | 4.115766 | 1.10E-08 |
| 308 | 0.410677 | 1.11E-09 | 4.118598 | 1.10E-08 |
| 309 | 0.409158 | 1.10E-09 | 4.121419 | 1.10E-08 |
| 310 | 0.407638 | 1.10E-09 | 4.124229 | 1.10E-08 |
| 311 | 0.406117 | 1.10E-09 | 4.12703 | 1.11E-08 |
| 312 | 0.404595 | 1.09E-09 | 4.12982 | 1.11E-08 |
| 313 | 0.403072 | 1.09E-09 | 4.1326 | 1.11E-08 |
| 314 | 0.401549 | 1.08E-09 | 4.135369 | 1.11E-08 |
| 315 | 0.400024 | 1.08E-09 | 4.138129 | 1.11E-08 |
| 316 | 0.398499 | 1.07E-09 | 4.140877 | 1.11E-08 |
| 317 | 0.396972 | 1.07E-09 | 4.143616 | 1.11E-08 |
| 318 | 0.395445 | 1.07E-09 | 4.146344 | 1.11E-08 |
| 319 | 0.393917 | 1.06E-09 | 4.149062 | 1.11E-08 |
| 320 | 0.392388 | 1.06E-09 | 4.151769 | 1.11E-08 |
| 321 | 0.390858 | 1.05E-09 | 4.154467 | 1.11E-08 |
| 322 | 0.389328 | 1.05E-09 | 4.157153 | 1.11E-08 |
| 323 | 0.387796 | 1.05E-09 | 4.15983 | 1.11E-08 |
| 324 | 0.386264 | 1.04E-09 | 4.162496 | 1.12E-08 |
| 325 | 0.38473 | 1.04E-09 | 4.165151 | 1.12E-08 |
| 326 | 0.383196 | 1.03E-09 | 4.167797 | 1.12E-08 |
| 327 | 0.381661 | 1.03E-09 | 4.170432 | 1.12E-08 |
| 328 | 0.380125 | 1.03E-09 | 4.173056 | 1.12E-08 |
| 329 | 0.378589 | 1.02E-09 | 4.17567 | 1.12E-08 |
| 330 | 0.377051 | 1.02E-09 | 4.178274 | 1.12E-08 |
| 331 | 0.375513 | 1.01E-09 | 4.180867 | 1.12E-08 |
| 332 | 0.373974 | 1.01E-09 | 4.18345 | 1.12E-08 |
| 333 | 0.372434 | 1.01E-09 | 4.186022 | 1.12E-08 |
| 334 | 0.370893 | 1.00E-09 | 4.188584 | 1.12E-08 |
| 335 | 0.369351 | 9.99E-10 | 4.191136 | 1.12E-08 |
| 336 | 0.367809 | 9.94E-10 | 4.193677 | 1.12E-08 |
| 337 | 0.366265 | 9.65E-10 | 4.196207 | 1.13E-08 |
| 338 | 0.364721 | 9.30E-10 | 4.198728 | 1.13E-08 |
| 339 | 0.363176 | 1.17E-09 | 4.201237 | 1.09E-08 |
| 340 | 0.361631 | 2.01E-09 | 4.203737 | 9.32E-09 |
| 341 | 0.360084 | 3.60E-09 | 4.206225 | 1.31E-08 |
| 342 | 0.358537 | 9.32E-09 | 4.208704 | 3.11E-08 |
| 343 | 0.356989 | 2.91E-08 | 4.211171 | 6.74E-08 |
| 344 | 0.35544 | 8.47E-08 | 4.213629 | 1.63E-07 |
| 345 | 0.35389 | 2.30E-07 | 4.216075 | 4.46E-07 |
| 346 | 0.35234 | 5.98E-07 | 4.218512 | 1.19E-06 |
| 347 | 0.350788 | 1.50E-06 | 4.220938 | 3.01E-06 |
| 348 | 0.349236 | 3.61E-06 | 4.223353 | 7.23E-06 |
| 349 | 0.347684 | 8.38E-06 | 4.225758 | 1.67E-05 |
| 350 | 0.34613 | 1.87E-05 | 4.228152 | 3.73E-05 |
| 351 | 0.344576 | 3.99E-05 | 4.230536 | 7.97E-05 |
| 352 | 0.343021 | 8.21E-05 | 4.232909 | 1.64E-04 |
| 353 | 0.341465 | 1.62E-04 | 4.235271 | 3.24E-04 |
| 354 | 0.339908 | 3.08E-04 | 4.237624 | 6.15E-04 |
| 355 | 0.338351 | 5.61E-04 | 4.239965 | 1.12E-03 |
| 356 | 0.336793 | 9.83E-04 | 4.242296 | 1.97E-03 |
| 357 | 0.335234 | 1.66E-03 | 4.244617 | 3.31E-03 |
| 358 | 0.333675 | 2.68E-03 | 4.246926 | 5.35E-03 |
| 359 | 0.332115 | 4.16E-03 | 4.249226 | 8.30E-03 |
| 360 | 0.330554 | 6.20E-03 | 4.251514 | 1.24E-02 |
| 361 | 0.328992 | 8.87E-03 | 4.253793 | 1.77E-02 |
| 362 | 0.32743 | 1.22E-02 | 4.25606 | 2.44E-02 |
| 363 | 0.325867 | 1.61E-02 | 4.258317 | 3.22E-02 |
| 364 | 0.324303 | 2.04E-02 | 4.260563 | 4.08E-02 |
| 365 | 0.322739 | 2.48E-02 | 4.262799 | 4.96E-02 |
| 366 | 0.321174 | 2.90E-02 | 4.265024 | 5.79E-02 |
| 367 | 0.319608 | 3.25E-02 | 4.267239 | 6.49E-02 |
| 368 | 0.318063 | 3.49E-02 | 4.269398 | 6.98E-02 |
| 369 | 0.487943 | 3.61E-02 | 3.929062 | 7.21E-02 |
| 370 | 0.956119 | 3.57E-02 | 2.992527 | 7.14E-02 |
| 371 | 1.129202 | 3.40E-02 | 2.645312 | 6.79E-02 |
| 372 | 1.198923 | 3.10E-02 | 2.504547 | 6.19E-02 |
| 373 | 1.236029 | 2.71E-02 | 2.428943 | 5.42E-02 |
| 374 | 1.258518 | 2.28E-02 | 2.382558 | 4.55E-02 |
| 375 | 1.272909 | 1.84E-02 | 2.352375 | 3.67E-02 |
| 376 | 1.282445 | 1.42E-02 | 2.33191 | 2.84E-02 |
| 377 | 1.288918 | 1.05E-02 | 2.317585 | 2.11E-02 |
| 378 | 1.293372 | 7.51E-03 | 2.307312 | 1.50E-02 |
| 379 | 1.296442 | 5.14E-03 | 2.29982 | 1.03E-02 |
| 380 | 1.298534 | 3.37E-03 | 2.294293 | 6.74E-03 |
| 381 | 1.29992 | 2.13E-03 | 2.290189 | 4.25E-03 |
| 382 | 1.300785 | 1.29E-03 | 2.287134 | 2.57E-03 |
| 383 | 1.301261 | 7.47E-04 | 2.284861 | 1.49E-03 |
| 384 | 1.301442 | 4.17E-04 | 2.283181 | 8.33E-04 |
| 385 | 1.301399 | 2.23E-04 | 2.281952 | 4.46E-04 |
| 386 | 1.301183 | 1.15E-04 | 2.28107 | 2.30E-04 |
| 387 | 1.300832 | 5.68E-05 | 2.280456 | 1.13E-04 |
| 388 | 1.300375 | 2.70E-05 | 2.280051 | 5.39E-05 |
| 389 | 1.299835 | 1.23E-05 | 2.279811 | 2.46E-05 |
| 390 | 1.299227 | 5.39E-06 | 2.279701 | 1.08E-05 |
| 391 | 1.298563 | 2.27E-06 | 2.279697 | 4.53E-06 |
| 392 | 1.297855 | 9.21E-07 | 2.279777 | 1.83E-06 |
| 393 | 1.297108 | 3.57E-07 | 2.279927 | 7.14E-07 |
| 394 | 1.296328 | 1.30E-07 | 2.280136 | 2.73E-07 |
| 395 | 1.29552 | 4.59E-08 | 2.280394 | 9.95E-08 |
| 396 | 1.294686 | 1.87E-08 | 2.280694 | 3.02E-08 |
| 397 | 1.29383 | 8.94E-09 | 2.281031 | 5.28E-09 |
| 398 | 1.292953 | 4.13E-09 | 2.2814 | 6.64E-09 |
| 399 | 1.292057 | 3.02E-09 | 2.281799 | 7.05E-09 |
| 400 | 1.291142 | 3.36E-09 | 2.282225 | 6.31E-09 |
| 401 | 1.29021 | 3.49E-09 | 2.282676 | 6.05E-09 |
| 402 | 1.289262 | 3.48E-09 | 2.283149 | 6.06E-09 |
| 403 | 1.288298 | 3.47E-09 | 2.283644 | 6.08E-09 |
| 404 | 1.287318 | 3.46E-09 | 2.284158 | 6.09E-09 |
| 405 | 1.286324 | 3.46E-09 | 2.284692 | 6.09E-09 |
| 406 | 1.285315 | 3.46E-09 | 2.285245 | 6.09E-09 |
| 407 | 1.284292 | 3.45E-09 | 2.285814 | 6.10E-09 |
| 408 | 1.283256 | 3.45E-09 | 2.2864 | 6.10E-09 |
| 409 | 1.282206 | 3.45E-09 | 2.287002 | 6.10E-09 |
| 410 | 1.281143 | 3.45E-09 | 2.287619 | 6.09E-09 |
| 411 | 1.280067 | 3.45E-09 | 2.28825 | 6.09E-09 |
| 412 | 1.278978 | 3.44E-09 | 2.288895 | 6.12E-09 |
| 413 | 1.277878 | 3.41E-09 | 2.289554 | 6.15E-09 |
| 414 | 1.276765 | 3.42E-09 | 2.290224 | 6.14E-09 |
| 415 | 1.275641 | 3.46E-09 | 2.290907 | 6.05E-09 |
| 416 | 1.274505 | 3.48E-09 | 2.291601 | 6.01E-09 |
| 417 | 1.273359 | 3.41E-09 | 2.292306 | 6.15E-09 |
| 418 | 1.272201 | 3.32E-09 | 2.293022 | 6.32E-09 |
| 419 | 1.271033 | 3.36E-09 | 2.293747 | 6.24E-09 |
| 420 | 1.269854 | 3.50E-09 | 2.294481 | 5.95E-09 |
| 421 | 1.268666 | 3.55E-09 | 2.295224 | 5.86E-09 |
| 422 | 1.267467 | 3.41E-09 | 2.295975 | 6.16E-09 |
| 423 | 1.266259 | 3.25E-09 | 2.296734 | 6.45E-09 |
| 424 | 1.265042 | 3.30E-09 | 2.2975 | 6.34E-09 |
| 425 | 1.263815 | 3.48E-09 | 2.298273 | 5.98E-09 |
| 426 | 1.262579 | 3.54E-09 | 2.299053 | 5.85E-09 |
| 427 | 1.261335 | 3.42E-09 | 2.299838 | 6.10E-09 |
| 428 | 1.260082 | 3.27E-09 | 2.300629 | 6.38E-09 |
| 429 | 1.258821 | 3.26E-09 | 2.301425 | 6.38E-09 |
| 430 | 1.257551 | 3.39E-09 | 2.302226 | 6.13E-09 |
| 431 | 1.256274 | 3.51E-09 | 2.303031 | 5.88E-09 |
| 432 | 1.254988 | 3.47E-09 | 2.30384 | 5.97E-09 |
| 433 | 1.253695 | 3.30E-09 | 2.304653 | 6.33E-09 |
| 434 | 1.252395 | 3.19E-09 | 2.30547 | 6.52E-09 |
| 435 | 1.251087 | 3.31E-09 | 2.306289 | 6.26E-09 |
| 436 | 1.249772 | 3.50E-09 | 2.307111 | 5.86E-09 |
| 437 | 1.248451 | 3.49E-09 | 2.307936 | 5.89E-09 |
| 438 | 1.247122 | 3.37E-09 | 2.308763 | 6.29E-09 |
| 439 | 1.245786 | 3.38E-09 | 2.309592 | 6.48E-09 |
| 440 | 1.244444 | 3.20E-09 | 2.310422 | 6.28E-09 |
| 441 | 1.243096 | 2.06E-09 | 2.311254 | 6.02E-09 |
| 442 | 1.241741 | 3.38E-09 | 2.312087 | 6.03E-09 |
| 443 | 1.24038 | 9.88E-09 | 2.312921 | 6.20E-09 |
| 444 | 1.239012 | 1.93E-08 | 2.313756 | 6.27E-09 |
| 445 | 1.237639 | 3.42E-08 | 2.314591 | 6.25E-09 |
| 446 | 1.23626 | 6.59E-08 | 2.315427 | 6.30E-09 |
| 447 | 1.234875 | 1.32E-07 | 2.316262 | 6.29E-09 |
| 448 | 1.233484 | 2.52E-07 | 2.317098 | 5.85E-09 |
| 449 | 1.232088 | 4.51E-07 | 2.317933 | 5.38E-09 |
| 450 | 1.230686 | 7.71E-07 | 2.318768 | 6.52E-09 |
| 451 | 1.229278 | 1.27E-06 | 2.319602 | 8.79E-09 |
| 452 | 1.227866 | 2.02E-06 | 2.320436 | 9.40E-09 |
| 453 | 1.226448 | 3.08E-06 | 2.321268 | 6.13E-09 |
| 454 | 1.225024 | 4.50E-06 | 2.3221 | 3.76E-09 |
| 455 | 1.223596 | 6.32E-06 | 2.32293 | 1.41E-08 |
| 456 | 1.222163 | 8.52E-06 | 2.323759 | 2.41E-08 |
| 457 | 1.220724 | 1.10E-05 | 2.324587 | 2.93E-08 |
| 458 | 1.219281 | 1.37E-05 | 2.325413 | 2.90E-08 |
| 459 | 1.217832 | 1.64E-05 | 2.326237 | 3.03E-08 |
| 460 | 1.216379 | 1.88E-05 | 2.32706 | 4.01E-08 |
| 461 | 1.214921 | 2.06E-05 | 2.32788 | 5.15E-08 |
| 462 | 1.213467 | 2.17E-05 | 2.328699 | 5.57E-08 |
| 463 | 1.212225 | 2.20E-05 | 2.329514 | 5.13E-08 |
| 464 | 1.210995 | 2.14E-05 | 2.330328 | 4.43E-08 |
| 465 | 1.209629 | 1.99E-05 | 2.33114 | 4.33E-08 |
| 466 | 1.208204 | 1.78E-05 | 2.331949 | 4.58E-08 |
| 467 | 1.206753 | 1.53E-05 | 2.332756 | 4.32E-08 |
| 468 | 1.205287 | 1.26E-05 | 2.333561 | 3.30E-08 |
| 469 | 1.203809 | 1.00E-05 | 2.334363 | 2.00E-08 |
| 470 | 1.202323 | 7.60E-06 | 2.335162 | 1.37E-08 |
| 471 | 1.200829 | 5.54E-06 | 2.335958 | 1.59E-08 |
| 472 | 1.199329 | 3.88E-06 | 2.336751 | 1.58E-08 |
| 473 | 1.197824 | 2.61E-06 | 2.337541 | 1.16E-08 |
| 474 | 1.196312 | 1.69E-06 | 2.338328 | 6.60E-09 |
| 475 | 1.194796 | 1.05E-06 | 2.339112 | 4.22E-09 |
| 476 | 1.193275 | 6.26E-07 | 2.339892 | 6.07E-09 |
| 477 | 1.19175 | 3.61E-07 | 2.340669 | 3.32E-08 |
| 478 | 1.19022 | 2.16E-07 | 2.341443 | 1.02E-07 |
| 479 | 1.188686 | 1.95E-07 | 2.342214 | 2.68E-07 |
| 480 | 1.187148 | 3.64E-07 | 2.342981 | 6.76E-07 |
| 481 | 1.185606 | 8.54E-07 | 2.343745 | 1.67E-06 |
| 482 | 1.18406 | 2.01E-06 | 2.344505 | 3.99E-06 |
| 483 | 1.182509 | 4.58E-06 | 2.345261 | 9.15E-06 |
| 484 | 1.180955 | 1.01E-05 | 2.346014 | 2.01E-05 |
| 485 | 1.179397 | 2.13E-05 | 2.346763 | 4.25E-05 |
| 486 | 1.177835 | 4.32E-05 | 2.347508 | 8.63E-05 |
| 487 | 1.17627 | 8.43E-05 | 2.348249 | 1.68E-04 |
| 488 | 1.1747 | 1.58E-04 | 2.348987 | 3.15E-04 |
| 489 | 1.173127 | 2.84E-04 | 2.34972 | 5.68E-04 |
| 490 | 1.17155 | 4.91E-04 | 2.35045 | 9.82E-04 |
| 491 | 1.169969 | 8.16E-04 | 2.351176 | 1.63E-03 |
| 492 | 1.168385 | 1.30E-03 | 2.351897 | 2.60E-03 |
| 493 | 1.166797 | 1.99E-03 | 2.352615 | 3.98E-03 |
| 494 | 1.165206 | 2.93E-03 | 2.353328 | 5.86E-03 |
| 495 | 1.163611 | 4.14E-03 | 2.354037 | 8.28E-03 |
| 496 | 1.162012 | 5.62E-03 | 2.354742 | 1.12E-02 |
| 497 | 1.16041 | 7.32E-03 | 2.355443 | 1.46E-02 |
| 498 | 1.158804 | 9.15E-03 | 2.356139 | 1.83E-02 |
| 499 | 1.157195 | 1.10E-02 | 2.356831 | 2.19E-02 |
| 500 | 1.155582 | 1.26E-02 | 2.357519 | 2.53E-02 |
