# Supplementary material for: A new method for identifying a fault in T-connected lines based on multiscale S-transform energy entropy and an extreme learning machine
Source: PLoS One. 2019 Aug 15;14(8):e0220870. doi: 10.1371/journal.pone.0220870 (PMC6695217; doi:10.1371/journal.pone.0220870)
Supplement: S16 Table — (DOCX) [file pone.0220870.s017.docx]

**S16 Table. The data obtained from Fig.21 is as follows.**

| BCG phase to ground short circuit occurring on transmission line AO at a distance of 150 km from O point, fault resistance of 100 Ω (fault initial angle of 45°) | | |
| --- | --- | --- |
| N-th sampling point | Not data lost | Lost 30 data near the wave head |
| 1 | 0.002017 | 0.002017 |
| 2 | 0.002085 | 2.09E-03 |
| 3 | 0.002154 | 2.15E-03 |
| 4 | 0.002224 | 2.22E-03 |
| 5 | 0.002295 | 2.30E-03 |
| 6 | 0.002367 | 2.37E-03 |
| 7 | 0.002439 | 2.44E-03 |
| 8 | 0.002512 | 2.51E-03 |
| 9 | 0.002585 | 2.58E-03 |
| 10 | 0.002658 | 2.66E-03 |
| 11 | 0.002732 | 2.73E-03 |
| 12 | 0.002807 | 2.81E-03 |
| 13 | 0.002881 | 2.88E-03 |
| 14 | 0.002955 | 2.96E-03 |
| 15 | 0.00303 | 3.03E-03 |
| 16 | 0.003104 | 3.10E-03 |
| 17 | 0.003178 | 3.18E-03 |
| 18 | 0.003251 | 3.25E-03 |
| 19 | 0.003324 | 3.32E-03 |
| 20 | 0.003397 | 3.40E-03 |
| 21 | 0.003469 | 3.47E-03 |
| 22 | 0.00354 | 3.54E-03 |
| 23 | 0.00361 | 3.61E-03 |
| 24 | 0.003679 | 3.68E-03 |
| 25 | 0.003747 | 3.75E-03 |
| 26 | 0.003814 | 3.81E-03 |
| 27 | 0.003879 | 3.88E-03 |
| 28 | 0.003943 | 3.94E-03 |
| 29 | 0.004006 | 4.01E-03 |
| 30 | 0.004066 | 4.07E-03 |
| 31 | 0.004125 | 4.13E-03 |
| 32 | 0.004183 | 4.18E-03 |
| 33 | 0.004238 | 4.24E-03 |
| 34 | 0.004291 | 4.29E-03 |
| 35 | 0.004342 | 0 |
| 36 | 0.00439 | 0 |
| 37 | 0.004436 | 0 |
| 38 | 0.00448 | 0 |
| 39 | 0.004522 | 0 |
| 40 | 0.00456 | 0 |
| 41 | 0.004596 | 0 |
| 42 | 0.004629 | 0 |
| 43 | 0.00466 | 0 |
| 44 | 0.004687 | 0 |
| 45 | 0.004712 | 0 |
| 46 | 0.004734 | 0 |
| 47 | 0.004752 | 0 |
| 48 | 0.004768 | 0 |
| 49 | 0.00478 | 0 |
| 50 | 0.00479 | 0 |
| 51 | 0.004796 | 0 |
| 52 | 0.004799 | 0 |
| 53 | 0.004799 | 0 |
| 54 | 0.004796 | 0 |
| 55 | 0.004789 | 0 |
| 56 | 0.00478 | 0 |
| 57 | 0.004767 | 0 |
| 58 | 0.004751 | 0 |
| 59 | 0.004732 | 0 |
| 60 | 0.004711 | 0 |
| 61 | 0.004686 | 0 |
| 62 | 0.004658 | 0 |
| 63 | 0.004627 | 0 |
| 64 | 0.004594 | 0 |
| 65 | 0.004557 | 4.56E-03 |
| 66 | 0.004518 | 4.52E-03 |
| 67 | 0.004477 | 4.48E-03 |
| 68 | 0.004433 | 4.43E-03 |
| 69 | 0.004386 | 4.39E-03 |
| 70 | 0.004337 | 4.34E-03 |
| 71 | 0.004286 | 4.29E-03 |
| 72 | 0.004232 | 4.23E-03 |
| 73 | 0.004177 | 4.18E-03 |
| 74 | 0.004119 | 4.12E-03 |
| 75 | 0.004059 | 4.06E-03 |
| 76 | 0.003998 | 4.00E-03 |
| 77 | 0.003935 | 3.94E-03 |
| 78 | 0.00387 | 3.87E-03 |
| 79 | 0.003804 | 3.80E-03 |
| 80 | 0.003737 | 3.74E-03 |
| 81 | 0.003668 | 3.67E-03 |
| 82 | 0.003598 | 3.60E-03 |
| 83 | 0.003528 | 3.53E-03 |
| 84 | 0.003456 | 3.46E-03 |
| 85 | 0.003383 | 3.38E-03 |
| 86 | 0.00331 | 3.31E-03 |
| 87 | 0.003236 | 3.24E-03 |
| 88 | 0.003162 | 3.16E-03 |
| 89 | 0.003087 | 3.09E-03 |
| 90 | 0.003012 | 3.01E-03 |
| 91 | 0.002937 | 2.94E-03 |
| 92 | 0.002862 | 2.86E-03 |
| 93 | 0.002787 | 2.79E-03 |
| 94 | 0.002712 | 2.71E-03 |
| 95 | 0.002638 | 2.64E-03 |
| 96 | 0.002563 | 2.56E-03 |
| 97 | 0.002489 | 2.49E-03 |
| 98 | 0.002416 | 2.42E-03 |
| 99 | 0.002343 | 2.34E-03 |
| 100 | 0.002271 | 2.27E-03 |
