# Supplementary material for: A new method for identifying a fault in T-connected lines based on multiscale S-transform energy entropy and an extreme learning machine
Source: PLoS One. 2019 Aug 15;14(8):e0220870. doi: 10.1371/journal.pone.0220870 (PMC6695217; doi:10.1371/journal.pone.0220870)
Supplement: S17 Table — (DOCX) [file pone.0220870.s018.docx]

**S17 Table. The data obtained from Fig.22 is as follows.**

| CG phase to ground short circuit occurring on transmission line BE at a distance of 280 km from O point, fault resistance of 200 Ω (fault initial angle of 25°) | | |
| --- | --- | --- |
| N-th sampling point | Not data lost | Lost 30 data near the wave head |
| 1 | 0.009604 | 9.60E-03 |
| 2 | 0.009925 | 9.93E-03 |
| 3 | 0.010251 | 1.03E-02 |
| 4 | 0.010581 | 1.06E-02 |
| 5 | 0.010914 | 1.09E-02 |
| 6 | 0.011251 | 1.13E-02 |
| 7 | 0.01159 | 1.16E-02 |
| 8 | 0.011932 | 1.19E-02 |
| 9 | 0.012277 | 1.23E-02 |
| 10 | 0.012623 | 1.26E-02 |
| 11 | 0.012971 | 1.30E-02 |
| 12 | 0.01332 | 1.33E-02 |
| 13 | 0.013669 | 1.37E-02 |
| 14 | 0.014019 | 1.40E-02 |
| 15 | 0.014368 | 1.44E-02 |
| 16 | 0.014717 | 1.47E-02 |
| 17 | 0.015065 | 1.51E-02 |
| 18 | 0.015411 | 1.54E-02 |
| 19 | 0.015754 | 1.58E-02 |
| 20 | 0.016095 | 1.61E-02 |
| 21 | 0.016433 | 1.64E-02 |
| 22 | 0.016768 | 1.68E-02 |
| 23 | 0.017098 | 1.71E-02 |
| 24 | 0.017423 | 1.74E-02 |
| 25 | 0.017743 | 1.77E-02 |
| 26 | 0.018058 | 1.81E-02 |
| 27 | 0.018366 | 1.84E-02 |
| 28 | 0.018668 | 1.87E-02 |
| 29 | 0.018963 | 1.90E-02 |
| 30 | 0.01925 | 1.92E-02 |
| 31 | 0.019528 | 1.95E-02 |
| 32 | 0.019798 | 1.98E-02 |
| 33 | 0.020059 | 2.01E-02 |
| 34 | 0.020311 | 2.03E-02 |
| 35 | 0.020552 | 0 |
| 36 | 0.020783 | 0 |
| 37 | 0.021003 | 0 |
| 38 | 0.021212 | 0 |
| 39 | 0.02141 | 0 |
| 40 | 0.021595 | 0 |
| 41 | 0.021768 | 0 |
| 42 | 0.021929 | 0 |
| 43 | 0.022076 | 0 |
| 44 | 0.022211 | 0 |
| 45 | 0.022332 | 0 |
| 46 | 0.022439 | 0 |
| 47 | 0.022533 | 0 |
| 48 | 0.022612 | 0 |
| 49 | 0.022678 | 0 |
| 50 | 0.022729 | 0 |
| 51 | 0.022766 | 0 |
| 52 | 0.022789 | 0 |
| 53 | 0.022797 | 0 |
| 54 | 0.022791 | 0 |
| 55 | 0.022771 | 0 |
| 56 | 0.022737 | 0 |
| 57 | 0.022688 | 0 |
| 58 | 0.022625 | 0 |
| 59 | 0.022549 | 0 |
| 60 | 0.022459 | 0 |
| 61 | 0.022355 | 0 |
| 62 | 0.022239 | 0 |
| 63 | 0.022109 | 0 |
| 64 | 0.021967 | 0 |
| 65 | 0.021813 | 2.18E-02 |
| 66 | 0.021647 | 2.16E-02 |
| 67 | 0.02147 | 2.15E-02 |
| 68 | 0.021281 | 2.13E-02 |
| 69 | 0.021083 | 2.11E-02 |
| 70 | 0.020874 | 2.09E-02 |
| 71 | 0.020655 | 2.07E-02 |
| 72 | 0.020427 | 2.04E-02 |
| 73 | 0.020191 | 2.02E-02 |
| 74 | 0.019947 | 1.99E-02 |
| 75 | 0.019696 | 1.97E-02 |
| 76 | 0.019438 | 1.94E-02 |
| 77 | 0.019174 | 1.92E-02 |
| 78 | 0.018905 | 1.89E-02 |
| 79 | 0.018631 | 1.86E-02 |
| 80 | 0.018354 | 1.84E-02 |
| 81 | 0.018073 | 1.81E-02 |
| 82 | 0.01779 | 1.78E-02 |
| 83 | 0.017505 | 1.75E-02 |
| 84 | 0.017219 | 1.72E-02 |
| 85 | 0.016933 | 1.69E-02 |
| 86 | 0.016648 | 1.66E-02 |
| 87 | 0.016364 | 1.64E-02 |
| 88 | 0.016083 | 1.61E-02 |
| 89 | 0.015805 | 1.58E-02 |
| 90 | 0.015531 | 1.55E-02 |
| 91 | 0.015262 | 1.53E-02 |
| 92 | 0.014999 | 1.50E-02 |
| 93 | 0.014743 | 1.47E-02 |
| 94 | 0.014494 | 1.45E-02 |
| 95 | 0.014253 | 1.43E-02 |
| 96 | 0.014022 | 1.40E-02 |
| 97 | 0.013801 | 1.38E-02 |
| 98 | 0.01359 | 1.36E-02 |
| 99 | 0.01339 | 1.34E-02 |
| 100 | 0.013203 | 1.32E-02 |
