# Supplementary material for: A new method for identifying a fault in T-connected lines based on multiscale S-transform energy entropy and an extreme learning machine
Source: PLoS One. 2019 Aug 15;14(8):e0220870. doi: 10.1371/journal.pone.0220870 (PMC6695217; doi:10.1371/journal.pone.0220870)
Supplement: S18 Table — (DOCX) [file pone.0220870.s019.docx]

**S18 Table. The data obtained from Fig.24 is as follows.**

| AG phase to ground short circuit occurring on transmission line BO at a distance of 110 km from O point, fault resistance of 100 Ω (fault initial angle of 5°) | | | | |
| --- | --- | --- | --- | --- |
| N-th sampling point | Not data lost | Data randomly lost 10 | Data randomly lost 20 | Data randomly lost 30 |
| 1 | 0.018965 | 0.018965 | 0.018965 | 0.018965 |
| 2 | 0.019594 | 0.019594 | 0.019594 | 0.019594 |
| 3 | 0.02023 | 0.02023 | 0.02023 | 0.02023 |
| 4 | 0.020873 | 0.020873 | 0.020873 | 0.020873 |
| 5 | 0.021523 | 0.021523 | 0.021523 | 0 |
| 6 | 0.022179 | 0.022179 | 0 | 0 |
| 7 | 0.02284 | 0.02284 | 0.02284 | 0.02284 |
| 8 | 0.023506 | 0.023506 | 0.023506 | 0.023506 |
| 9 | 0.024176 | 0.024176 | 0.024176 | 0.024176 |
| 10 | 0.024849 | 0.024849 | 0.024849 | 0 |
| 11 | 0.025524 | 0.025524 | 0.025524 | 0.025524 |
| 12 | 0.026201 | 0.026201 | 0.026201 | 0.026201 |
| 13 | 0.026879 | 0.026879 | 0.026879 | 0.026879 |
| 14 | 0.027556 | 0.027556 | 0.027556 | 0.027556 |
| 15 | 0.028232 | 0.028232 | 0.028232 | 0.028232 |
| 16 | 0.028907 | 0.028907 | 0.028907 | 0.028907 |
| 17 | 0.029578 | 0.029578 | 0.029578 | 0.029578 |
| 18 | 0.030245 | 0.030245 | 0 | 0.030245 |
| 19 | 0.030908 | 0.030908 | 0 | 0.030908 |
| 20 | 0.031565 | 0.031565 | 0 | 0 |
| 21 | 0.032215 | 0.032215 | 0.032215 | 0 |
| 22 | 0.032858 | 0.032858 | 0.032858 | 0.032858 |
| 23 | 0.033491 | 0.033491 | 0.033491 | 0 |
| 24 | 0.034115 | 0.034115 | 0.034115 | 0.034115 |
| 25 | 0.034728 | 0.034728 | 0.034728 | 0.034728 |
| 26 | 0.03533 | 0.03533 | 0.03533 | 0 |
| 27 | 0.035919 | 0.035919 | 0.035919 | 0 |
| 28 | 0.036494 | 0 | 0.036494 | 0.036494 |
| 29 | 0.037054 | 0.037054 | 0 | 0.037054 |
| 30 | 0.037599 | 0.037599 | 0 | 0.037599 |
| 31 | 0.038127 | 0.038127 | 0.038127 | 0.038127 |
| 32 | 0.038638 | 0.038638 | 0 | 0 |
| 33 | 0.039131 | 0.039131 | 0.039131 | 0.039131 |
| 34 | 0.039604 | 0.039604 | 0.039604 | 0.039604 |
| 35 | 0.040057 | 0.040057 | 0.040057 | 0.040057 |
| 36 | 0.04049 | 0 | 0.04049 | 0 |
| 37 | 0.0409 | 0.0409 | 0.0409 | 0.0409 |
| 38 | 0.041289 | 0.041289 | 0.041289 | 0.041289 |
| 39 | 0.041654 | 0.041654 | 0.041654 | 0 |
| 40 | 0.041995 | 0.041995 | 0.041995 | 0.041995 |
| 41 | 0.042312 | 0 | 0.042312 | 0.042312 |
| 42 | 0.042603 | 0.042603 | 0 | 0 |
| 43 | 0.042869 | 0.042869 | 0.042869 | 0.042869 |
| 44 | 0.043109 | 0 | 0.043109 | 0 |
| 45 | 0.043323 | 0.043323 | 0 | 0.043323 |
| 46 | 0.043509 | 0.043509 | 0.043509 | 0 |
| 47 | 0.043668 | 0.043668 | 0.043668 | 0.043668 |
| 48 | 0.0438 | 0.0438 | 0.0438 | 0 |
| 49 | 0.043903 | 0.043903 | 0.043903 | 0 |
| 50 | 0.043979 | 0.043979 | 0.043979 | 0.043979 |
| 51 | 0.044026 | 0.044026 | 0.044026 | 0.044026 |
| 52 | 0.044045 | 0.044045 | 0.044045 | 0 |
| 53 | 0.044035 | 0.044035 | 0.044035 | 0.044035 |
| 54 | 0.043997 | 0.043997 | 0.043997 | 0.043997 |
| 55 | 0.043931 | 0.043931 | 0.043931 | 0.043931 |
| 56 | 0.043837 | 0.043837 | 0 | 0 |
| 57 | 0.043714 | 0.043714 | 0.043714 | 0.043714 |
| 58 | 0.043564 | 0.043564 | 0.043564 | 0 |
| 59 | 0.043387 | 0.043387 | 0.043387 | 0.043387 |
| 60 | 0.043182 | 0.043182 | 0 | 0.043182 |
| 61 | 0.04295 | 0 | 0.04295 | 0.04295 |
| 62 | 0.042692 | 0.042692 | 0.042692 | 0 |
| 63 | 0.042409 | 0.042409 | 0.042409 | 0 |
| 64 | 0.0421 | 0.0421 | 0.0421 | 0.0421 |
| 65 | 0.041766 | 0 | 0.041766 | 0.041766 |
| 66 | 0.041408 | 0.041408 | 0.041408 | 0.041408 |
| 67 | 0.041027 | 0.041027 | 0.041027 | 0 |
| 68 | 0.040623 | 0.040623 | 0.040623 | 0.040623 |
| 69 | 0.040197 | 0 | 0.040197 | 0.040197 |
| 70 | 0.03975 | 0.03975 | 0.03975 | 0.03975 |
| 71 | 0.039282 | 0 | 0.039282 | 0.039282 |
| 72 | 0.038795 | 0.038795 | 0.038795 | 0 |
| 73 | 0.038288 | 0.038288 | 0.038288 | 0 |
| 74 | 0.037764 | 0.037764 | 0.037764 | 0.037764 |
| 75 | 0.037223 | 0.037223 | 0.037223 | 0.037223 |
| 76 | 0.036666 | 0.036666 | 0.036666 | 0.036666 |
| 77 | 0.036094 | 0.036094 | 0.036094 | 0.036094 |
| 78 | 0.035508 | 0.035508 | 0 | 0.035508 |
| 79 | 0.034908 | 0.034908 | 0.034908 | 0.034908 |
| 80 | 0.034296 | 0.034296 | 0.034296 | 0.034296 |
| 81 | 0.033673 | 0 | 0.033673 | 0.033673 |
| 82 | 0.03304 | 0.03304 | 0.03304 | 0.03304 |
| 83 | 0.032397 | 0.032397 | 0.032397 | 0.032397 |
| 84 | 0.031746 | 0.031746 | 0 | 0 |
| 85 | 0.031088 | 0.031088 | 0 | 0.031088 |
| 86 | 0.030423 | 0.030423 | 0.030423 | 0.030423 |
| 87 | 0.029753 | 0.029753 | 0.029753 | 0.029753 |
| 88 | 0.029078 | 0.029078 | 0.029078 | 0.029078 |
| 89 | 0.028399 | 0.028399 | 0.028399 | 0 |
| 90 | 0.027718 | 0.027718 | 0.027718 | 0 |
| 91 | 0.027036 | 0.027036 | 0.027036 | 0.027036 |
| 92 | 0.026352 | 0.026352 | 0 | 0.026352 |
| 93 | 0.025668 | 0.025668 | 0 | 0.025668 |
| 94 | 0.024986 | 0 | 0 | 0.024986 |
| 95 | 0.024304 | 0.024304 | 0.024304 | 0.024304 |
| 96 | 0.023626 | 0.023626 | 0.023626 | 0 |
| 97 | 0.02295 | 0.02295 | 0 | 0 |
| 98 | 0.022278 | 0.022278 | 0.022278 | 0 |
| 99 | 0.021611 | 0.021611 | 0 | 0.021611 |
| 100 | 0.020949 | 0.020949 | 0 | 0.020949 |
