# Supplementary material for: A new method for identifying a fault in T-connected lines based on multiscale S-transform energy entropy and an extreme learning machine
Source: PLoS One. 2019 Aug 15;14(8):e0220870. doi: 10.1371/journal.pone.0220870 (PMC6695217; doi:10.1371/journal.pone.0220870)
Supplement: S19 Table — (DOCX) [file pone.0220870.s020.docx]

**S19 Table.** **The data obtained from Fig.25 is as follows.**

| ACG phase to ground short circuit occurring on transmission line CF at a distance of 230 km from O point, fault resistance of 50 Ω (fault initial angle of 45°) | | | | |
| --- | --- | --- | --- | --- |
| N-th sampling point | Not data lost | Data randomly lost 10 | Data randomly lost 20 | Data randomly lost 30 |
| 1 | 0.072572 | 0.072572 | 0.072572 | 0 |
| 2 | 0.074974 | 0.074974 | 0.074974 | 0.074974 |
| 3 | 0.077406 | 0 | 0.077406 | 0.077406 |
| 4 | 0.079865 | 0.079865 | 0 | 0.079865 |
| 5 | 0.08235 | 0.08235 | 0.08235 | 0 |
| 6 | 0.084858 | 0.084858 | 0.084858 | 0.084858 |
| 7 | 0.087386 | 0.087386 | 0.087386 | 0.087386 |
| 8 | 0.089931 | 0.089931 | 0 | 0.089931 |
| 9 | 0.092492 | 0.092492 | 0.092492 | 0.092492 |
| 10 | 0.095064 | 0.095064 | 0.095064 | 0 |
| 11 | 0.097645 | 0.097645 | 0.097645 | 0.097645 |
| 12 | 0.100232 | 0.100232 | 0.100232 | 0.100232 |
| 13 | 0.102821 | 0 | 0 | 0.102821 |
| 14 | 0.105409 | 0.105409 | 0.105409 | 0.105409 |
| 15 | 0.107993 | 0 | 0 | 0.107993 |
| 16 | 0.110569 | 0.110569 | 0.110569 | 0 |
| 17 | 0.113135 | 0 | 0.113135 | 0.113135 |
| 18 | 0.115685 | 0.115685 | 0 | 0.115685 |
| 19 | 0.118217 | 0.118217 | 0 | 0.118217 |
| 20 | 0.120726 | 0.120726 | 0.120726 | 0.120726 |
| 21 | 0.12321 | 0.12321 | 0.12321 | 0.12321 |
| 22 | 0.125664 | 0.125664 | 0.125664 | 0.125664 |
| 23 | 0.128084 | 0.128084 | 0 | 0.128084 |
| 24 | 0.130467 | 0.130467 | 0 | 0.130467 |
| 25 | 0.132809 | 0.132809 | 0.132809 | 0 |
| 26 | 0.135106 | 0.135106 | 0.135106 | 0.135106 |
| 27 | 0.137354 | 0.137354 | 0.137354 | 0.137354 |
| 28 | 0.13955 | 0.13955 | 0.13955 | 0 |
| 29 | 0.14169 | 0.14169 | 0.14169 | 0.14169 |
| 30 | 0.14377 | 0.14377 | 0.14377 | 0 |
| 31 | 0.145787 | 0 | 0.145787 | 0 |
| 32 | 0.147737 | 0.147737 | 0.147737 | 0.147737 |
| 33 | 0.149616 | 0.149616 | 0.149616 | 0 |
| 34 | 0.151422 | 0 | 0.151422 | 0 |
| 35 | 0.153151 | 0.153151 | 0.153151 | 0.153151 |
| 36 | 0.154801 | 0.154801 | 0 | 0.154801 |
| 37 | 0.156367 | 0.156367 | 0.156367 | 0.156367 |
| 38 | 0.157847 | 0.157847 | 0.157847 | 0.157847 |
| 39 | 0.159239 | 0 | 0.159239 | 0 |
| 40 | 0.16054 | 0.16054 | 0.16054 | 0 |
| 41 | 0.161747 | 0.161747 | 0.161747 | 0 |
| 42 | 0.162858 | 0.162858 | 0.162858 | 0 |
| 43 | 0.163872 | 0.163872 | 0 | 0.163872 |
| 44 | 0.164785 | 0.164785 | 0.164785 | 0 |
| 45 | 0.165597 | 0.165597 | 0 | 0.165597 |
| 46 | 0.166305 | 0.166305 | 0.166305 | 0.166305 |
| 47 | 0.16691 | 0.16691 | 0.16691 | 0.16691 |
| 48 | 0.167408 | 0.167408 | 0.167408 | 0.167408 |
| 49 | 0.1678 | 0.1678 | 0.1678 | 0 |
| 50 | 0.168084 | 0.168084 | 0.168084 | 0 |
| 51 | 0.16826 | 0.16826 | 0.16826 | 0.16826 |
| 52 | 0.168328 | 0.168328 | 0.168328 | 0 |
| 53 | 0.168288 | 0.168288 | 0 | 0.168288 |
| 54 | 0.168139 | 0.168139 | 0.168139 | 0.168139 |
| 55 | 0.167882 | 0.167882 | 0.167882 | 0.167882 |
| 56 | 0.167517 | 0.167517 | 0 | 0 |
| 57 | 0.167046 | 0.167046 | 0.167046 | 0.167046 |
| 58 | 0.166468 | 0.166468 | 0.166468 | 0 |
| 59 | 0.165786 | 0.165786 | 0 | 0.165786 |
| 60 | 0.165 | 0 | 0.165 | 0.165 |
| 61 | 0.164112 | 0.164112 | 0.164112 | 0.164112 |
| 62 | 0.163124 | 0.163124 | 0.163124 | 0.163124 |
| 63 | 0.162037 | 0.162037 | 0 | 0.162037 |
| 64 | 0.160853 | 0.160853 | 0.160853 | 0.160853 |
| 65 | 0.159576 | 0.159576 | 0.159576 | 0 |
| 66 | 0.158206 | 0.158206 | 0.158206 | 0.158206 |
| 67 | 0.156747 | 0.156747 | 0.156747 | 0 |
| 68 | 0.155202 | 0.155202 | 0.155202 | 0.155202 |
| 69 | 0.153572 | 0 | 0.153572 | 0.153572 |
| 70 | 0.151862 | 0.151862 | 0.151862 | 0.151862 |
| 71 | 0.150074 | 0.150074 | 0.150074 | 0.150074 |
| 72 | 0.148212 | 0.148212 | 0.148212 | 0.148212 |
| 73 | 0.146278 | 0.146278 | 0.146278 | 0.146278 |
| 74 | 0.144276 | 0.144276 | 0.144276 | 0 |
| 75 | 0.14221 | 0.14221 | 0.14221 | 0.14221 |
| 76 | 0.140084 | 0.140084 | 0.140084 | 0 |
| 77 | 0.1379 | 0.1379 | 0.1379 | 0 |
| 78 | 0.135662 | 0.135662 | 0.135662 | 0 |
| 79 | 0.133375 | 0.133375 | 0.133375 | 0 |
| 80 | 0.131041 | 0.131041 | 0.131041 | 0.131041 |
| 81 | 0.128665 | 0.128665 | 0.128665 | 0.128665 |
| 82 | 0.126251 | 0 | 0 | 0.126251 |
| 83 | 0.123802 | 0.123802 | 0 | 0 |
| 84 | 0.121321 | 0.121321 | 0.121321 | 0.121321 |
| 85 | 0.118814 | 0.118814 | 0.118814 | 0.118814 |
| 86 | 0.116283 | 0.116283 | 0.116283 | 0.116283 |
| 87 | 0.113733 | 0.113733 | 0.113733 | 0.113733 |
| 88 | 0.111166 | 0.111166 | 0.111166 | 0.111166 |
| 89 | 0.108586 | 0.108586 | 0.108586 | 0.108586 |
| 90 | 0.105998 | 0.105998 | 0.105998 | 0.105998 |
| 91 | 0.103404 | 0.103404 | 0.103404 | 0.103404 |
| 92 | 0.100808 | 0.100808 | 0 | 0.100808 |
| 93 | 0.098214 | 0.098214 | 0.098214 | 0.098214 |
| 94 | 0.095623 | 0.095623 | 0 | 0 |
| 95 | 0.093041 | 0.093041 | 0.093041 | 0.093041 |
| 96 | 0.090469 | 0.090469 | 0.090469 | 0 |
| 97 | 0.08791 | 0.08791 | 0.08791 | 0.08791 |
| 98 | 0.085368 | 0.085368 | 0.085368 | 0.085368 |
| 99 | 0.082845 | 0.082845 | 0 | 0.082845 |
| 100 | 0.080343 | 0.080343 | 0.080343 | 0.080343 |
