# Supplementary material for: A new method for identifying a fault in T-connected lines based on multiscale S-transform energy entropy and an extreme learning machine
Source: PLoS One. 2019 Aug 15;14(8):e0220870. doi: 10.1371/journal.pone.0220870 (PMC6695217; doi:10.1371/journal.pone.0220870)
Supplement: S20 Table — (DOCX) [file pone.0220870.s021.docx]

**S20 Table.** **The partial data obtained from Fig.28 is as follows.**

| ABG phase to ground short circuit occurring on transmission line BO at a distance of 130 km from O point, fault resistance of 50 Ω (fault initial angle of 45°) | | | | |
| --- | --- | --- | --- | --- |
| N-th sampling point | Original current | original current s-transformed | Original current signal-to-noise ratio is 30db | Original current signal-to-noise ratio is 30db s-transformed |
| 501 | -0.8697 | 2.33E-09 | -0.84225 | 4.90E-03 |
| 502 | -0.87068 | 2.33E-09 | -0.86973 | 4.97E-03 |
| 503 | -0.87166 | 2.34E-09 | -0.88421 | 4.96E-03 |
| 504 | -0.87264 | 2.34E-09 | -0.87301 | 4.89E-03 |
| 505 | -0.87361 | 2.34E-09 | -0.8762 | 4.77E-03 |
| 506 | -0.87458 | 2.34E-09 | -0.85922 | 4.66E-03 |
| 507 | -0.87555 | 2.35E-09 | -0.88876 | 4.61E-03 |
| 508 | -0.87652 | 2.35E-09 | -0.8817 | 4.65E-03 |
| 509 | -0.87748 | 2.35E-09 | -0.85132 | 4.75E-03 |
| 510 | -0.87844 | 2.35E-09 | -0.8885 | 4.88E-03 |
| 511 | -0.8794 | 2.36E-09 | -0.83733 | 4.98E-03 |
| 512 | -0.88036 | 2.36E-09 | -0.82222 | 4.96E-03 |
| 513 | -0.88132 | 2.36E-09 | -0.87292 | 4.78E-03 |
| 514 | -0.88227 | 2.36E-09 | -0.86685 | 4.43E-03 |
| 515 | -0.88323 | 2.37E-09 | -0.8854 | 3.92E-03 |
| 516 | -0.88418 | 2.37E-09 | -0.85129 | 3.30E-03 |
| 517 | -0.88512 | 2.37E-09 | -0.89917 | 2.68E-03 |
| 518 | -0.88607 | 2.37E-09 | -0.8909 | 2.30E-03 |
| 519 | -0.88701 | 2.38E-09 | -0.88262 | 2.49E-03 |
| 520 | -0.88796 | 2.38E-09 | -0.88933 | 3.24E-03 |
| 521 | -0.8889 | 2.38E-09 | -0.8546 | 4.30E-03 |
| 522 | -0.88983 | 2.38E-09 | -0.91534 | 5.47E-03 |
| 523 | -0.89077 | 2.39E-09 | -0.86609 | 6.63E-03 |
| 524 | -0.8917 | 2.39E-09 | -0.87049 | 7.70E-03 |
| 525 | -0.89263 | 2.39E-09 | -0.87947 | 8.62E-03 |
| 526 | -0.89356 | 2.39E-09 | -0.95588 | 9.36E-03 |
| 527 | -0.89449 | 2.40E-09 | -0.8994 | 9.90E-03 |
| 528 | -0.89542 | 2.40E-09 | -0.93698 | 1.02E-02 |
| 529 | -0.89634 | 2.40E-09 | -0.88149 | 1.03E-02 |
| 530 | -0.89726 | 2.40E-09 | -0.87856 | 1.03E-02 |
| 531 | -0.89818 | 2.41E-09 | -0.93437 | 9.99E-03 |
| 532 | -0.89909 | 2.41E-09 | -0.8952 | 9.52E-03 |
| 533 | -0.90001 | 2.41E-09 | -0.84635 | 8.87E-03 |
| 534 | -0.90092 | 2.41E-09 | -0.8625 | 8.08E-03 |
| 535 | -0.90183 | 2.41E-09 | -0.93277 | 7.19E-03 |
| 536 | -0.90274 | 2.42E-09 | -0.88403 | 6.28E-03 |
| 537 | -0.90365 | 2.46E-09 | -0.92341 | 5.45E-03 |
| 538 | -0.90455 | 2.44E-09 | -0.9375 | 4.80E-03 |
| 539 | -0.90545 | 2.06E-09 | -0.87691 | 4.42E-03 |
| 540 | -0.90635 | 1.71E-09 | -0.88009 | 4.38E-03 |
| 541 | -0.90725 | 5.75E-09 | -0.90241 | 4.72E-03 |
| 542 | -0.90814 | 1.54E-08 | -0.84648 | 5.35E-03 |
| 543 | -0.90904 | 3.84E-08 | -0.90014 | 6.19E-03 |
| 544 | -0.90993 | 1.03E-07 | -0.91673 | 7.10E-03 |
| 545 | -0.91081 | 2.78E-07 | -0.93067 | 7.96E-03 |
| 546 | -0.9117 | 7.21E-07 | -0.98463 | 8.67E-03 |
| 547 | -0.91258 | 1.78E-06 | -0.96276 | 9.16E-03 |
| 548 | -0.91347 | 4.21E-06 | -0.91371 | 9.40E-03 |
| 549 | -0.91435 | 9.59E-06 | -0.89896 | 9.41E-03 |
| 550 | -0.91522 | 2.10E-05 | -0.9597 | 9.23E-03 |
| 551 | -0.9161 | 4.41E-05 | -0.91757 | 8.92E-03 |
| 552 | -0.91697 | 8.89E-05 | -0.92611 | 8.56E-03 |
| 553 | -0.91784 | 1.72E-04 | -0.91045 | 8.22E-03 |
| 554 | -0.91871 | 3.21E-04 | -0.91333 | 7.92E-03 |
| 555 | -0.91958 | 5.74E-04 | -0.89928 | 7.62E-03 |
| 556 | -0.92044 | 9.85E-04 | -0.94508 | 7.25E-03 |
| 557 | -0.92131 | 1.62E-03 | -0.87375 | 6.69E-03 |
| 558 | -0.92217 | 2.57E-03 | -0.89015 | 5.76E-03 |
| 559 | -0.92303 | 3.91E-03 | -0.92322 | 4.34E-03 |
| 560 | -0.92388 | 5.70E-03 | -0.91018 | 2.29E-03 |
| 561 | -0.92473 | 7.99E-03 | -0.94998 | 7.01E-04 |
| 562 | -0.92559 | 1.07E-02 | -0.90666 | 4.06E-03 |
| 563 | -0.92643 | 1.39E-02 | -0.8774 | 8.14E-03 |
| 564 | -0.92728 | 1.72E-02 | -0.90896 | 1.26E-02 |
| 565 | -0.92813 | 2.04E-02 | -0.917 | 1.71E-02 |
| 566 | -0.92897 | 2.33E-02 | -0.85939 | 2.13E-02 |
| 567 | -0.92981 | 2.56E-02 | -0.8774 | 2.47E-02 |
| 568 | -0.93015 | 2.69E-02 | -0.92639 | 2.70E-02 |
| 569 | -0.6268 | 2.71E-02 | -0.7004 | 2.79E-02 |
| 570 | -0.38826 | 2.62E-02 | -0.38612 | 2.74E-02 |
| 571 | -0.30213 | 2.44E-02 | -0.29415 | 2.55E-02 |
| 572 | -0.26361 | 2.18E-02 | -0.32339 | 2.27E-02 |
| 573 | -0.24152 | 1.86E-02 | -0.24152 | 1.91E-02 |
| 574 | -0.22717 | 1.53E-02 | -0.21934 | 1.53E-02 |
| 575 | -0.21749 | 1.21E-02 | -0.27035 | 1.18E-02 |
| 576 | -0.21086 | 9.14E-03 | -0.22668 | 8.74E-03 |
| 577 | -0.20625 | 6.64E-03 | -0.23034 | 6.50E-03 |
| 578 | -0.20301 | 4.64E-03 | -0.19895 | 5.13E-03 |
| 579 | -0.20072 | 3.11E-03 | -0.18431 | 4.50E-03 |
| 580 | -0.19909 | 2.00E-03 | -0.15454 | 4.28E-03 |
| 581 | -0.19793 | 1.24E-03 | -0.19147 | 4.19E-03 |
| 582 | -0.19712 | 7.35E-04 | -0.16384 | 4.11E-03 |
| 583 | -0.19656 | 4.20E-04 | -0.16739 | 3.98E-03 |
| 584 | -0.19619 | 2.30E-04 | -0.17103 | 3.79E-03 |
| 585 | -0.19596 | 1.21E-04 | -0.12901 | 3.52E-03 |
| 586 | -0.19584 | 6.14E-05 | -0.15106 | 3.16E-03 |
| 587 | -0.19581 | 2.99E-05 | -0.18512 | 2.68E-03 |
| 588 | -0.19583 | 1.40E-05 | -0.13995 | 2.10E-03 |
| 589 | -0.19591 | 6.28E-06 | -0.21133 | 1.48E-03 |
| 590 | -0.19602 | 2.72E-06 | -0.1609 | 1.06E-03 |
| 591 | -0.19617 | 1.13E-06 | -0.19021 | 1.33E-03 |
| 592 | -0.19633 | 4.51E-07 | -0.21559 | 2.12E-03 |
| 593 | -0.19652 | 1.73E-07 | -0.20479 | 3.04E-03 |
| 594 | -0.19673 | 6.44E-08 | -0.21403 | 3.96E-03 |
| 595 | -0.19694 | 2.28E-08 | -0.24717 | 4.80E-03 |
| 596 | -0.19717 | 7.32E-09 | -0.21693 | 5.51E-03 |
| 597 | -0.19741 | 2.14E-09 | -0.16377 | 6.06E-03 |
| 598 | -0.19766 | 1.05E-09 | -0.21462 | 6.46E-03 |
| 599 | -0.19792 | 7.60E-10 | -0.18418 | 6.69E-03 |
| 600 | -0.19818 | 5.57E-10 | -0.20872 | 6.78E-03 |
