# Supplementary material for: A new method for identifying a fault in T-connected lines based on multiscale S-transform energy entropy and an extreme learning machine
Source: PLoS One. 2019 Aug 15;14(8):e0220870. doi: 10.1371/journal.pone.0220870 (PMC6695217; doi:10.1371/journal.pone.0220870)
Supplement: S21 Table — (DOCX) [file pone.0220870.s022.docx]

**S21 Table.** **The partial data obtained from Fig.29 is as follows.**

| ABG phase to ground short circuit occurring on transmission line AD at a distance of 430 km from O point, fault resistance of 50 Ω (fault initial angle of 25°) | | | | |
| --- | --- | --- | --- | --- |
| N-th sampling point | Original current | original current s-transformed | Original current signal-to-noise ratio is 30db | Original current signal-to-noise ratio is 30db s-transformed |
| 301 | -0.63824 | 1.71E-09 | -0.60747 | 1.25E-02 |
| 302 | -0.63957 | 1.72E-09 | -0.62479 | 1.18E-02 |
| 303 | -0.6409 | 1.72E-09 | -0.62477 | 1.10E-02 |
| 304 | -0.64223 | 1.72E-09 | -0.69691 | 1.01E-02 |
| 305 | -0.64356 | 1.73E-09 | -0.6209 | 9.34E-03 |
| 306 | -0.64489 | 1.73E-09 | -0.66341 | 8.75E-03 |
| 307 | -0.64622 | 1.73E-09 | -0.65859 | 8.43E-03 |
| 308 | -0.64754 | 1.74E-09 | -0.60198 | 8.41E-03 |
| 309 | -0.64886 | 1.74E-09 | -0.67453 | 8.67E-03 |
| 310 | -0.65019 | 1.74E-09 | -0.64613 | 9.14E-03 |
| 311 | -0.65151 | 1.75E-09 | -0.67351 | 9.70E-03 |
| 312 | -0.65282 | 1.75E-09 | -0.62588 | 1.03E-02 |
| 313 | -0.65414 | 1.76E-09 | -0.6389 | 1.07E-02 |
| 314 | -0.65546 | 1.76E-09 | -0.63594 | 1.11E-02 |
| 315 | -0.65677 | 1.76E-09 | -0.6671 | 1.12E-02 |
| 316 | -0.65808 | 1.77E-09 | -0.7123 | 1.10E-02 |
| 317 | -0.65939 | 1.77E-09 | -0.64683 | 1.06E-02 |
| 318 | -0.6607 | 1.77E-09 | -0.63086 | 9.98E-03 |
| 319 | -0.66201 | 1.78E-09 | -0.69197 | 9.13E-03 |
| 320 | -0.66332 | 1.78E-09 | -0.68182 | 8.14E-03 |
| 321 | -0.66462 | 1.78E-09 | -0.69833 | 7.07E-03 |
| 322 | -0.66593 | 1.79E-09 | -0.67745 | 5.99E-03 |
| 323 | -0.66723 | 1.79E-09 | -0.68702 | 4.99E-03 |
| 324 | -0.66853 | 1.80E-09 | -0.65327 | 4.12E-03 |
| 325 | -0.66983 | 1.80E-09 | -0.65809 | 3.46E-03 |
| 326 | -0.67112 | 1.77E-09 | -0.68042 | 3.04E-03 |
| 327 | -0.67242 | 1.74E-09 | -0.62893 | 2.86E-03 |
| 328 | -0.67371 | 2.10E-09 | -0.66856 | 2.88E-03 |
| 329 | -0.675 | 3.24E-09 | -0.70504 | 3.07E-03 |
| 330 | -0.6763 | 5.08E-09 | -0.66158 | 3.40E-03 |
| 331 | -0.67758 | 1.27E-08 | -0.69589 | 3.84E-03 |
| 332 | -0.67887 | 4.15E-08 | -0.70419 | 4.37E-03 |
| 333 | -0.68016 | 1.22E-07 | -0.66979 | 4.94E-03 |
| 334 | -0.68144 | 3.33E-07 | -0.68773 | 5.49E-03 |
| 335 | -0.68272 | 8.70E-07 | -0.68713 | 5.92E-03 |
| 336 | -0.68401 | 2.19E-06 | -0.67617 | 6.16E-03 |
| 337 | -0.68529 | 5.32E-06 | -0.64937 | 6.12E-03 |
| 338 | -0.68656 | 1.24E-05 | -0.62879 | 5.80E-03 |
| 339 | -0.68784 | 2.78E-05 | -0.69937 | 5.23E-03 |
| 340 | -0.68911 | 5.98E-05 | -0.72021 | 4.55E-03 |
| 341 | -0.69039 | 1.24E-04 | -0.67217 | 4.01E-03 |
| 342 | -0.69166 | 2.45E-04 | -0.70538 | 3.87E-03 |
| 343 | -0.69293 | 4.68E-04 | -0.71478 | 4.13E-03 |
| 344 | -0.6942 | 8.58E-04 | -0.64024 | 4.47E-03 |
| 345 | -0.69546 | 1.51E-03 | -0.69308 | 4.53E-03 |
| 346 | -0.69673 | 2.56E-03 | -0.72176 | 4.06E-03 |
| 347 | -0.69799 | 4.15E-03 | -0.71286 | 3.11E-03 |
| 348 | -0.69925 | 6.47E-03 | -0.75025 | 3.12E-03 |
| 349 | -0.70051 | 9.70E-03 | -0.679 | 6.09E-03 |
| 350 | -0.70177 | 1.39E-02 | -0.7057 | 1.12E-02 |
| 351 | -0.70303 | 1.93E-02 | -0.7324 | 1.77E-02 |
| 352 | -0.70428 | 2.55E-02 | -0.69197 | 2.55E-02 |
| 353 | -0.70554 | 3.25E-02 | -0.70383 | 3.41E-02 |
| 354 | -0.70679 | 3.97E-02 | -0.69187 | 4.29E-02 |
| 355 | -0.70804 | 4.65E-02 | -0.78153 | 5.12E-02 |
| 356 | -0.70929 | 5.23E-02 | -0.71458 | 5.84E-02 |
| 357 | -0.71054 | 5.65E-02 | -0.74634 | 6.37E-02 |
| 358 | -0.90902 | 5.85E-02 | -0.85177 | 6.66E-02 |
| 359 | -1.67551 | 5.82E-02 | -1.68516 | 6.69E-02 |
| 360 | -1.71817 | 5.55E-02 | -1.76077 | 6.45E-02 |
| 361 | -1.73376 | 5.09E-02 | -1.75573 | 5.98E-02 |
| 362 | -1.74349 | 4.47E-02 | -1.72427 | 5.34E-02 |
| 363 | -1.74986 | 3.77E-02 | -1.72792 | 4.59E-02 |
| 364 | -1.75418 | 3.05E-02 | -1.75316 | 3.80E-02 |
| 365 | -1.75732 | 2.37E-02 | -1.74857 | 3.04E-02 |
| 366 | -1.75983 | 1.77E-02 | -1.78216 | 2.36E-02 |
| 367 | -1.76202 | 1.27E-02 | -1.74353 | 1.78E-02 |
| 368 | -1.76404 | 8.70E-03 | -1.75985 | 1.32E-02 |
| 369 | -1.766 | 5.74E-03 | -1.76165 | 9.82E-03 |
| 370 | -1.76792 | 3.63E-03 | -1.76376 | 7.53E-03 |
| 371 | -1.76984 | 2.21E-03 | -1.76515 | 6.17E-03 |
| 372 | -1.77176 | 1.29E-03 | -1.77783 | 5.58E-03 |
| 373 | -1.77368 | 7.24E-04 | -1.80303 | 5.54E-03 |
| 374 | -1.77562 | 3.90E-04 | -1.77084 | 5.88E-03 |
| 375 | -1.77756 | 2.02E-04 | -1.84288 | 6.43E-03 |
| 376 | -1.77951 | 1.00E-04 | -1.79623 | 7.07E-03 |
| 377 | -1.78147 | 4.78E-05 | -1.80503 | 7.68E-03 |
| 378 | -1.78343 | 2.19E-05 | -1.7778 | 8.21E-03 |
| 379 | -1.78539 | 9.66E-06 | -1.77385 | 8.60E-03 |
| 380 | -1.78735 | 4.09E-06 | -1.84073 | 8.87E-03 |
| 381 | -1.78931 | 1.66E-06 | -1.81419 | 9.02E-03 |
| 382 | -1.79127 | 6.46E-07 | -1.8183 | 9.09E-03 |
| 383 | -1.79322 | 2.39E-07 | -1.80083 | 9.11E-03 |
| 384 | -1.79517 | 8.82E-08 | -1.75702 | 9.12E-03 |
| 385 | -1.79711 | 3.51E-08 | -1.80701 | 9.12E-03 |
| 386 | -1.79905 | 1.35E-08 | -1.7939 | 9.12E-03 |
| 387 | -1.80099 | 3.91E-09 | -1.77582 | 9.09E-03 |
| 388 | -1.80291 | 3.90E-09 | -1.82098 | 8.99E-03 |
| 389 | -1.80483 | 4.84E-09 | -1.73275 | 8.79E-03 |
| 390 | -1.80674 | 4.93E-09 | -1.80445 | 8.45E-03 |
| 391 | -1.80865 | 4.87E-09 | -1.87235 | 7.96E-03 |
| 392 | -1.81054 | 4.86E-09 | -1.82502 | 7.30E-03 |
| 393 | -1.81243 | 4.86E-09 | -1.87076 | 6.47E-03 |
| 394 | -1.81431 | 4.87E-09 | -1.86181 | 5.50E-03 |
| 395 | -1.81618 | 4.87E-09 | -1.76864 | 4.46E-03 |
| 396 | -1.81805 | 4.88E-09 | -1.84602 | 3.49E-03 |
| 397 | -1.8199 | 4.88E-09 | -1.82407 | 2.82E-03 |
| 398 | -1.82175 | 4.88E-09 | -1.82207 | 2.72E-03 |
| 399 | -1.82359 | 4.89E-09 | -1.83284 | 3.12E-03 |
| 400 | -1.82542 | 4.91E-09 | -1.88074 | 3.69E-03 |
