# Supplementary material for: A new method for identifying a fault in T-connected lines based on multiscale S-transform energy entropy and an extreme learning machine
Source: PLoS One. 2019 Aug 15;14(8):e0220870. doi: 10.1371/journal.pone.0220870 (PMC6695217; doi:10.1371/journal.pone.0220870)
Supplement: Attachment — Submitted filename: Reviewers comments.pdf [file pone.0220870.s023.pdf]

The paper is not written with technical English and is really hard to follow. It is just like translated from some sorts of software. Please proofread the whole paper carefully to correct all grammar mistakes. Though the paper is with good novelty, I cannot recommend the paper for publication unless the English is improved.

Introduction part is poor at criticizing literature works. It is not clear why the authors choose the current back wave multiscale S-transform energy entropy and an extreme learning machine

Inductance and capacitance of the unit length line are not reflected in Figure 1 and the resistance is ignored

Figure 4. Figure 5. Figure 6 and so on without the abscissa.

The author proposed the single-hidden layer feedforward neural network (SLFN) learning algorithm，but the following paper is about ELM, What is the connection between SLFN and ELM?
